# Supplementary material for: AP39, a novel mitochondria-targeted hydrogen sulfide donor ameliorates doxorubicin-induced cardiotoxicity by regulating the AMPK/UCP2 pathway
Source: PLoS One. 2024 Apr 3;19(4):e0300261. doi: 10.1371/journal.pone.0300261 (PMC10990198; doi:10.1371/journal.pone.0300261)

FIG.2

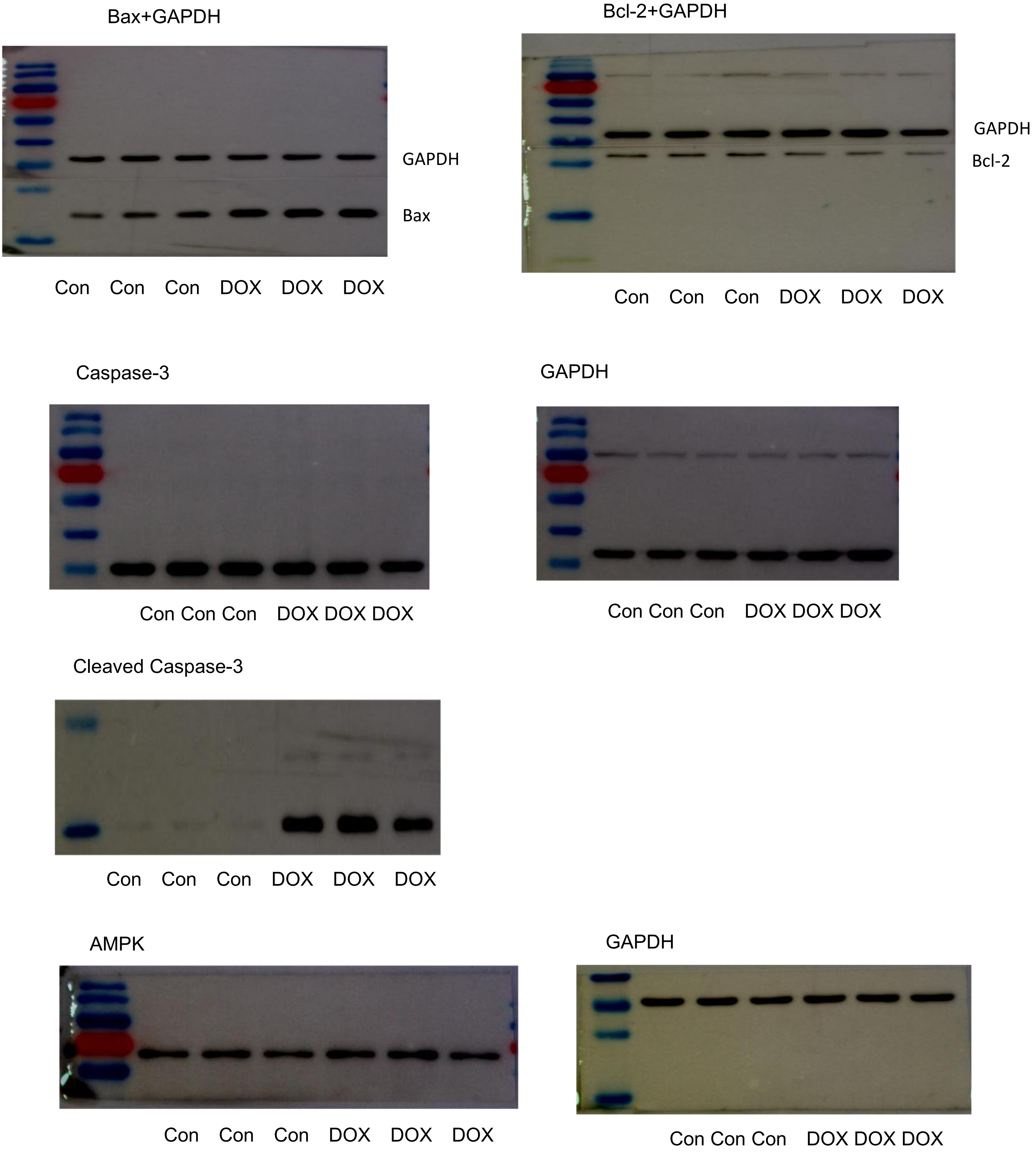
Bax+GAPDH
GAPDH
Bax
Con Con Con DOX DOX DOX
Bcl-2+GAPDH
GAPDH
Bcl-2
Con Con Con DOX DOX DOX
Caspase-3
Con Con Con DOX DOX DOX
GAPDH
Con Con Con DOX DOX DOX
Cleaved Caspase-3
Con Con Con DOX DOX DOX
AMPK
Con Con Con DOX DOX DOX
GAPDH
Con Con Con DOX DOX DOX

AMPK+GAPDH

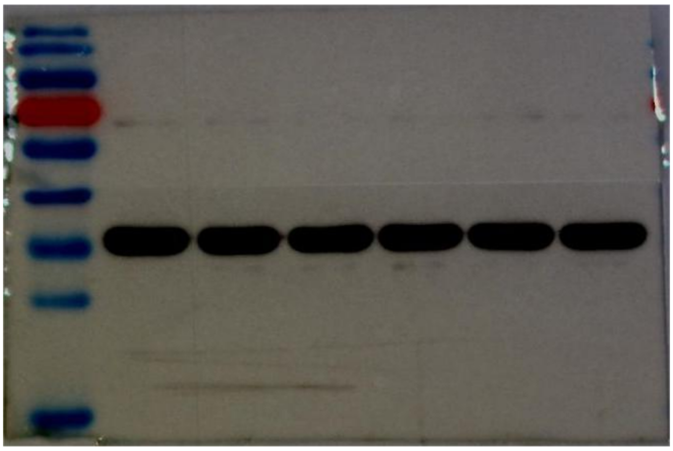

AMPK

GAPDH

Con Con Con DOX DOX DOX

P-AMPK

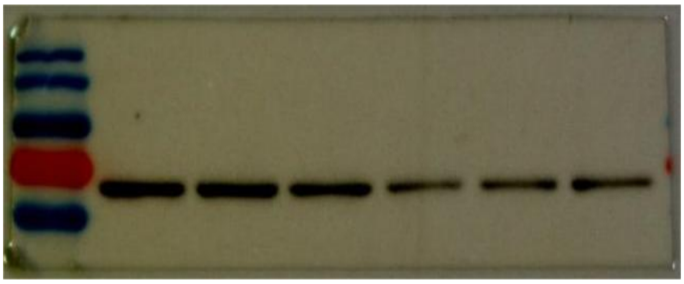

Con Con Con DOX DOX DOX

P-AMPK+GAPDH

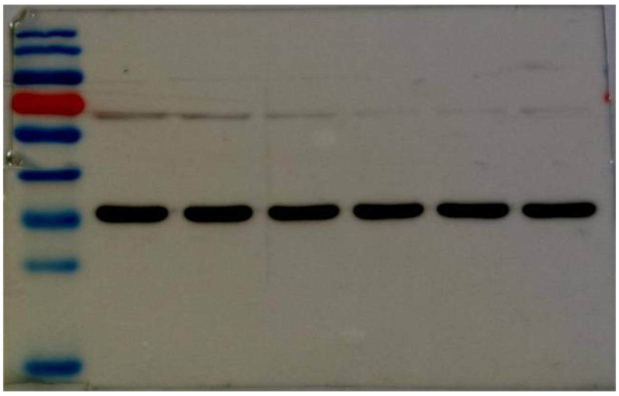

P-AMPK

GAPDH

Con Con Con DOX DOX DOX

UCP2

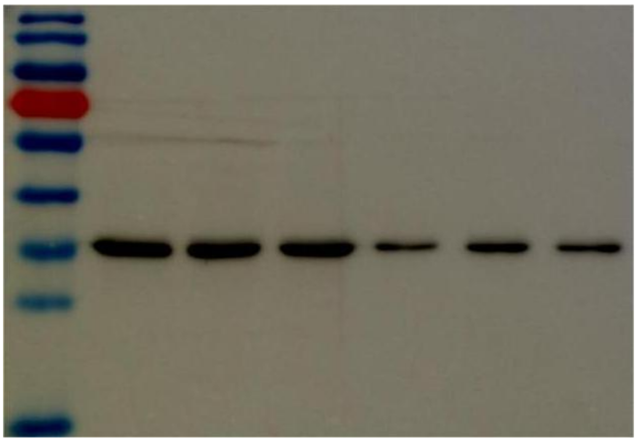

Con Con Con DOX DOX DOX

GAPDH

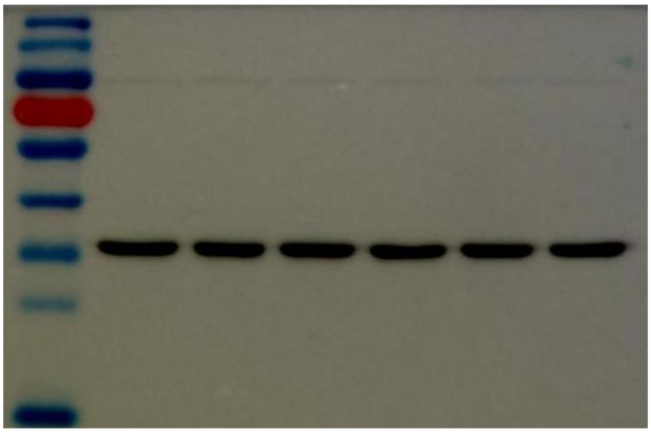

Con Con Con DOX DOX DOX

FIG.4

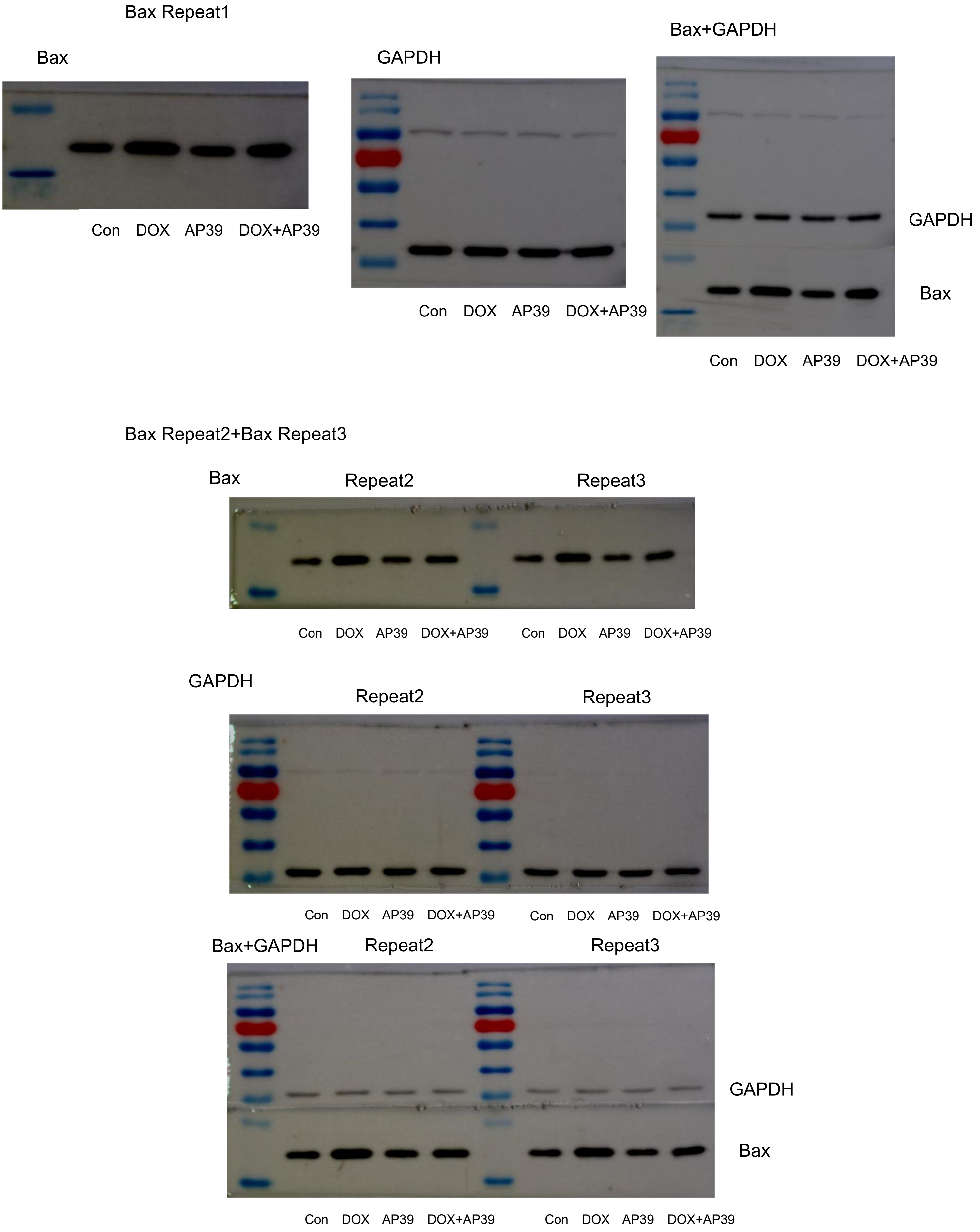
Bax Repeat1
Bax
Con DOX AP39 DOX+AP39
GAPDH
Con DOX AP39 DOX+AP39
Bax+GAPDH
GAPDH
Bax
Con DOX AP39 DOX+AP39
Bax Repeat2+Bax Repeat3
Bax
Repeat2
Repeat3
Con DOX AP39 DOX+AP39 Con DOX AP39 DOX+AP39
GAPDH
Repeat2
Repeat3
Con DOX AP39 DOX+AP39 Con DOX AP39 DOX+AP39
Bax+GAPDH
Repeat2
Repeat3
GAPDH
Bax
Con DOX AP39 DOX+AP39 Con DOX AP39 DOX+AP39

Bcl-2 Repeat1+Bcl-2 Repeat2

Bcl-2 Repeat1 Repeat2

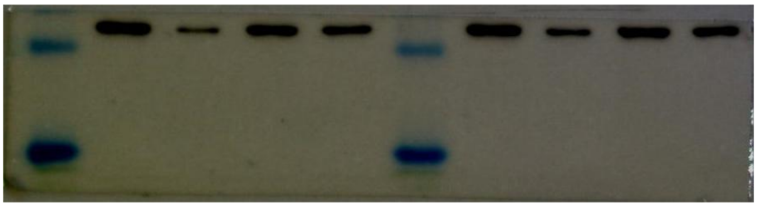

Con DOX AP39 DOX+AP39 Con DOX AP39 DOX+AP39

GAPDH Repeat1 Repeat2

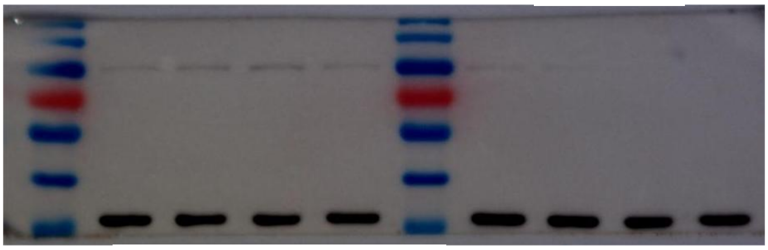

Con DOX AP39 DOX+AP39 Con DOX AP39 DOX+AP39

Bcl-2+GAPDH Repeat1 Repeat2

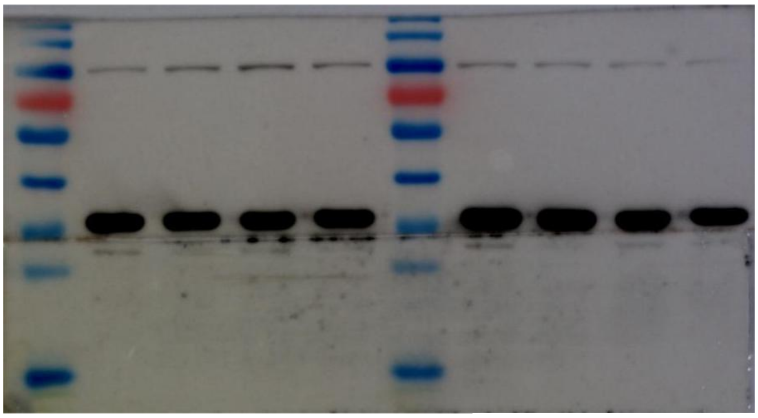

GAPDH

Bcl-2

Con DOX AP39 DOX+AP39 Con DOX AP39 DOX+AP39

Bcl-2 Repeat3

Bcl-2

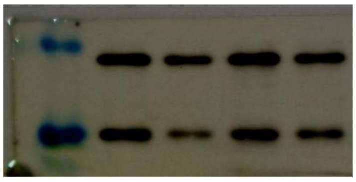

Con DOX AP39 DOX+AP39

GAPDH

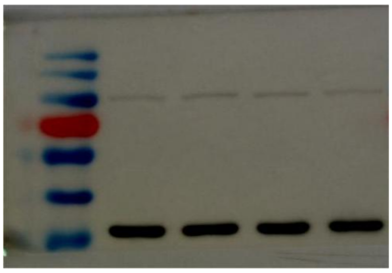

Con DOX AP39 DOX+AP39

Bcl-2+GAPDH

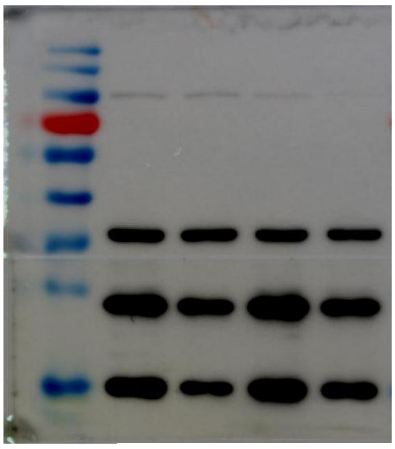

GAPDH

Bcl-2

Con DOX AP39 DOX+AP39

Cleaved Caspase-3 and Caspase-3 Repeat1+Repeat2

Repeat1 Repeat2

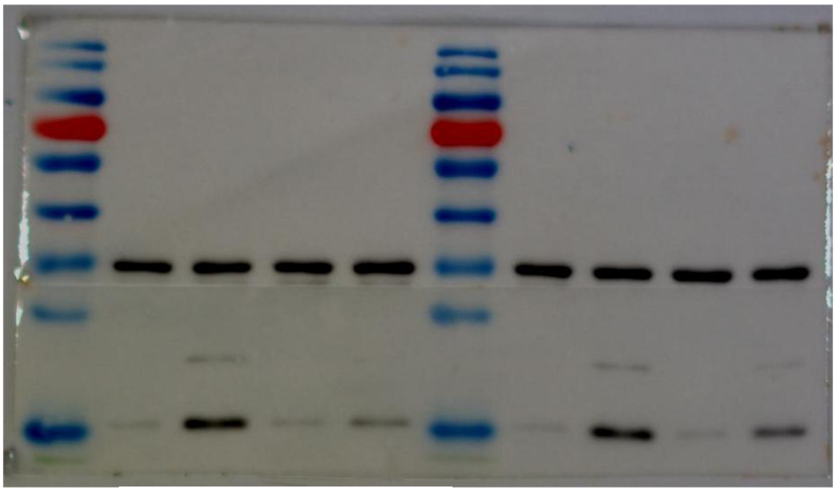

Caspase-3

Cleaved Caspase-3

Con DOX AP39 DOX+AP39 Con DOX AP39 DOX+AP39

GAPDH Repeat1 Repeat2

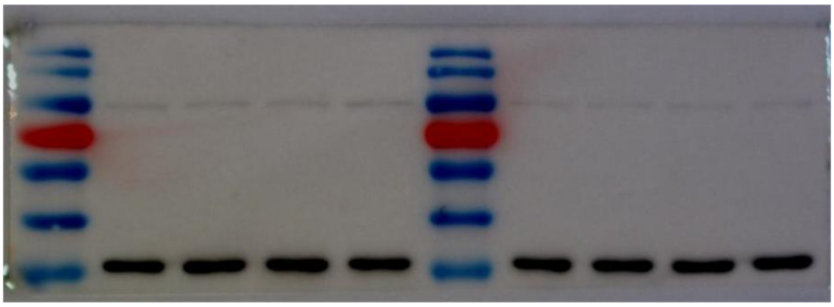

Con DOX AP39 DOX+AP39 Con DOX AP39 DOX+AP39

Cleaved Caspase-3 and Caspase-3 Repeat3+Repeat4

Repeat3 Repeat4

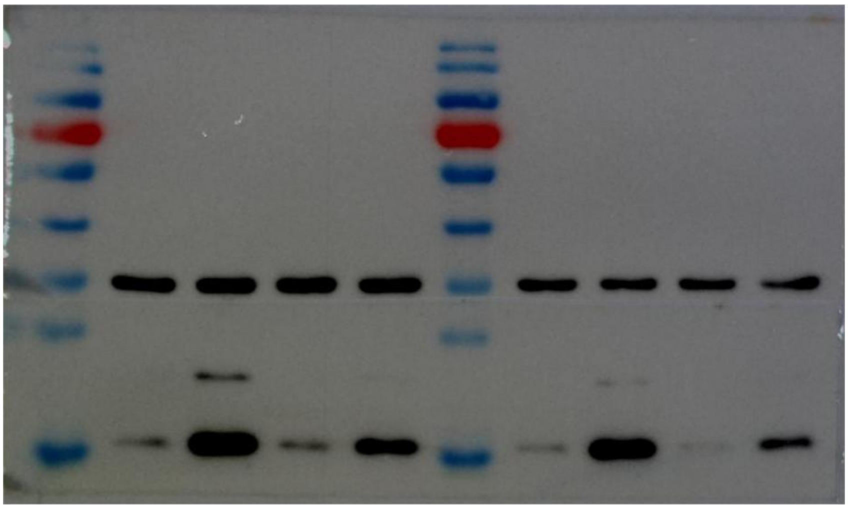

Caspase-3

Cleaved Caspase-3

Con DOX AP39 DOX+AP39 Con DOX AP39 DOX+AP39

GAPDH Repeat3 Repeat4

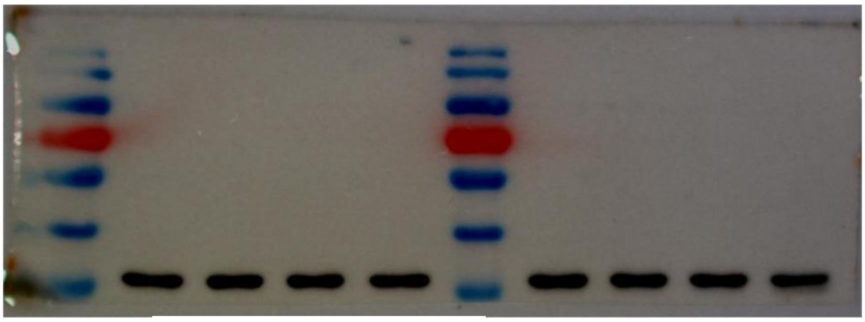

Con DOX AP39 DOX+AP39 Con DOX AP39 DOX+AP39

p-AMPK and AMPK Repeat1

AMPK

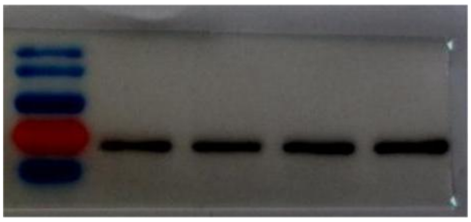

Con DOX AP39 DOX+AP39

AMPK+GAPDH

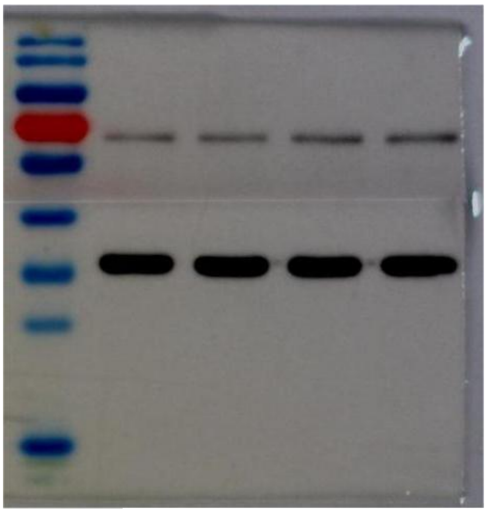

AMPK

GAPDH

Con DOX AP39 DOX+AP39

P-AMPK

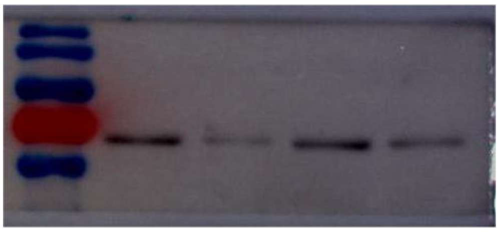

Con DOX AP39 DOX+AP39

P-AMPK+GAPDH

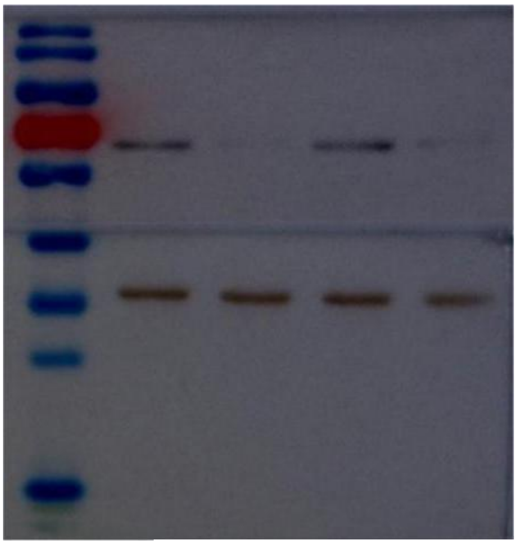

P-AMPK

GAPDH

Con DOX AP39 DOX+AP39

p-AMPK and AMPK Repeat2+Repeat3

AMPK

Repeat2 Repeat3

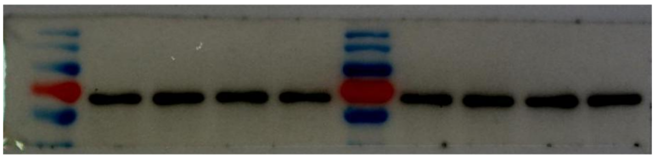

Con DOX AP39 DOX+AP39 Con DOX AP39 DOX+AP39

AMPK+GAPDH

Repeat2 Repeat3

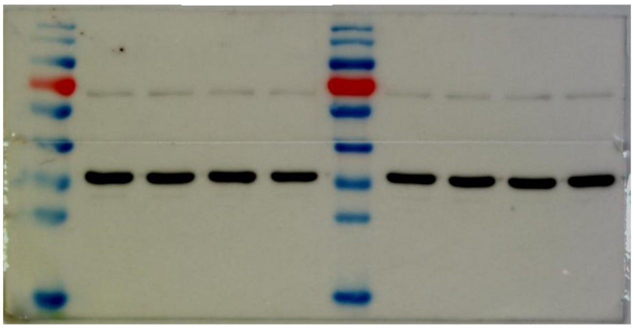

AMPK

GAPDH

Con DOX AP39 DOX+AP39 Con DOX AP39 DOX+AP39

P-AMPK

Repeat2 Repeat3

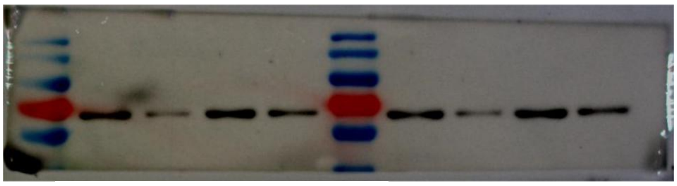

Con DOX AP39 DOX+AP39 Con DOX AP39 DOX+AP39

P-AMPK+GAPDH

Repeat2 Repeat3

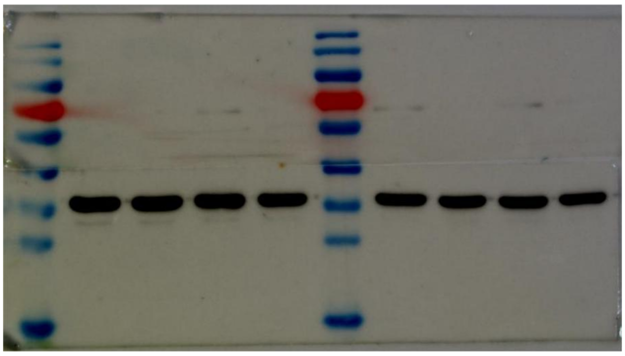

P-AMPK

GAPDH

Con DOX AP39 DOX+AP39 Con DOX AP39 DOX+AP39

UCP2 Repeat1+Repeat2

UCP2

Repeat1 Repeat2

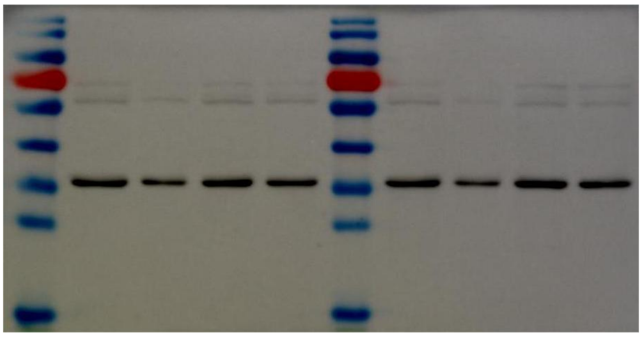

Con DOX AP39 DOX+AP39 Con DOX AP39 DOX+AP39

GAPDH

Repeat1 Repeat2

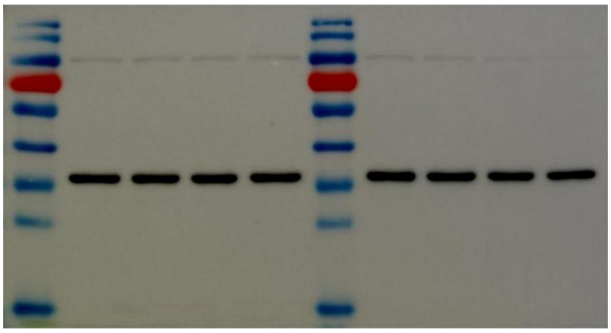

Con DOX AP39 DOX+AP39 Con DOX AP39 DOX+AP39

UCP2 Repeat3+Repeat4

UCP2

Repeat3 Repeat4

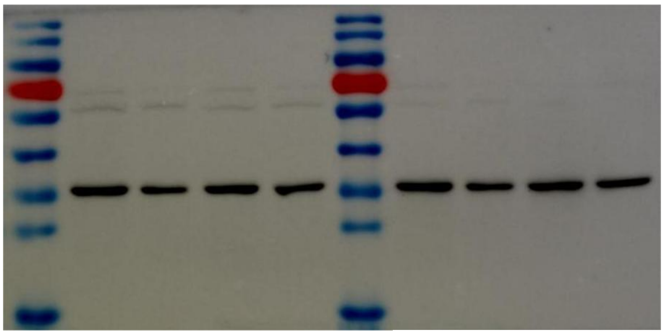

Con DOX AP39 DOX+AP39 Con DOX AP39 DOX+AP39

GAPDH

Repeat3 Repeat4

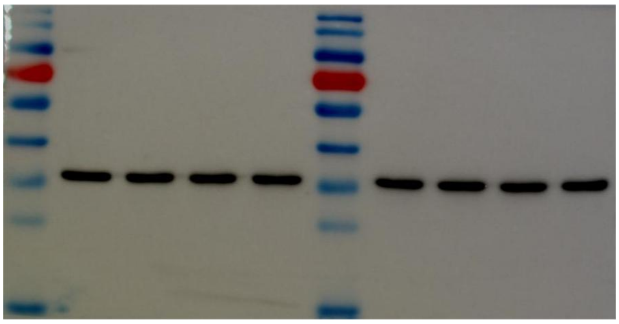

Con DOX AP39 DOX+AP39 Con DOX AP39 DOX+AP39

FIG.5

AMPK

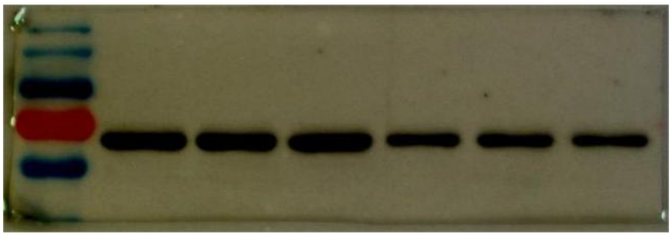

Con Con Con CC CC CC

AMPK+GAPDH

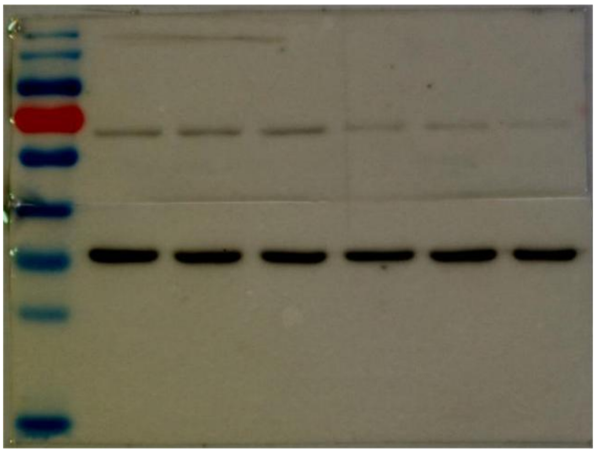

AMPK

GAPDH

Con Con Con CC CC CC

P-AMPK

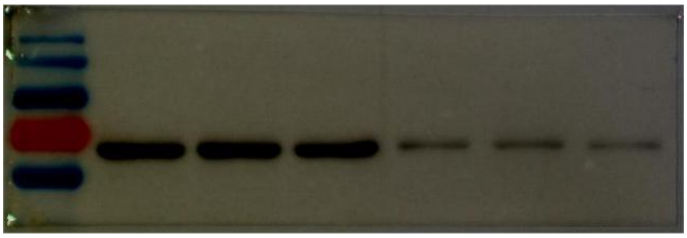

Con Con Con CC CC CC

P-AMPK+GAPDH

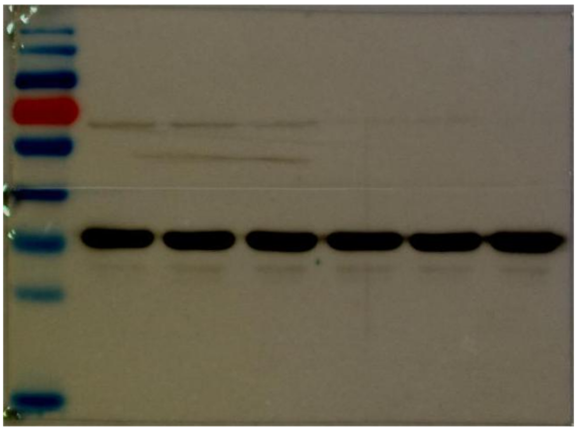

P-AMPK

GAPDH

Con Con Con CC CC CC

Bax Repeat1

Bax

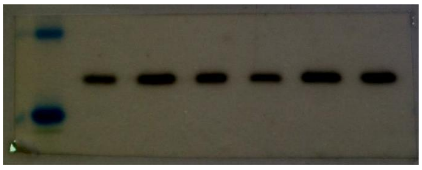

DOX	–	+	+	–	+	+
AP39	–	–	+	–	–	+
CC	–	–	–	+	+	+

GAPDH

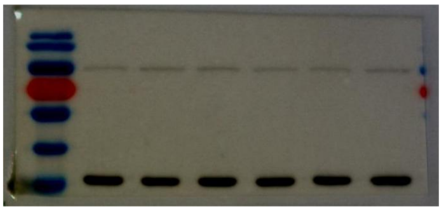

DOX	–	+	+	–	+	+
AP39	–	–	+	–	–	+
CC	–	–	–	+	+	+

Bax+GAPDH

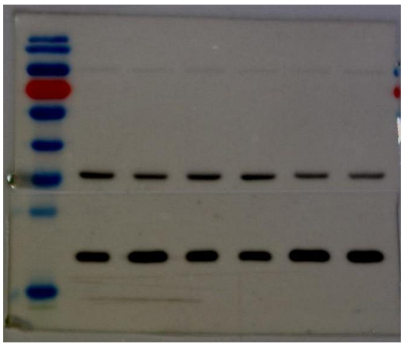

DOX	–	+	+	–	+	+
AP39	–	–	+	–	–	+
CC	–	–	–	+	+	+

Bax Repeat2

Bax

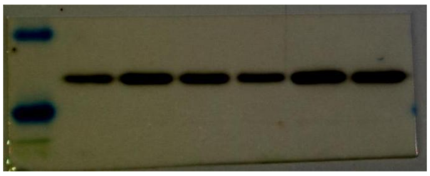

DOX	–	+	+	–	+	+
AP39	–	–	+	–	–	+
CC	–	–	–	+	+	+

GAPDH

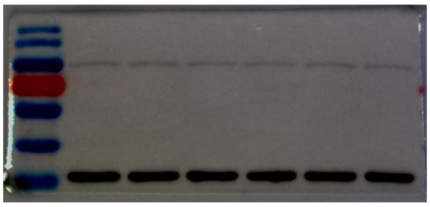

DOX	–	+	+	–	+	+
AP39	–	–	+	–	–	+
CC	–	–	–	+	+	+

Bax+GAPDH

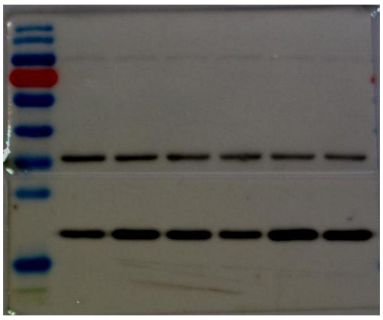

DOX	–	+	+	–	+	+
AP39	–	–	+	–	–	+
CC	–	–	–	+	+	+

Bax Repeat3

Bax

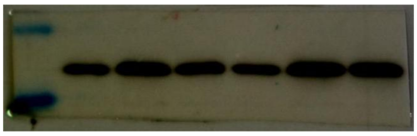

DOX	–	+	+	–	+	+
AP39	–	–	+	–	–	+
CC	–	–	–	+	+	+

GAPDH

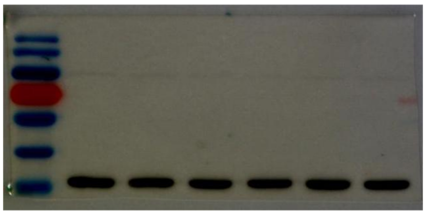

DOX	–	+	+	–	+	+
AP39	–	–	+	–	–	+
CC	–	–	–	+	+	+

Bax+GAPDH

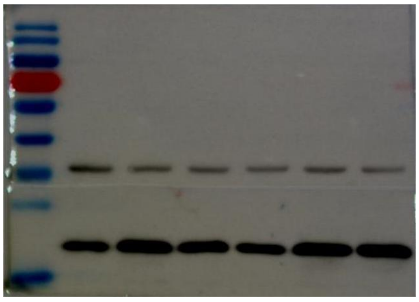

DOX	–	+	+	–	+	+
AP39	–	–	+	–	–	+
CC	–	–	–	+	+	+

Bcl-2 Repeat1

Bcl-2

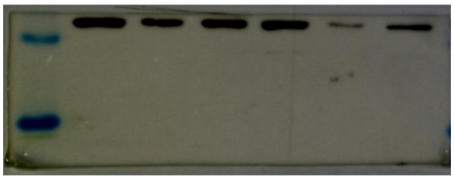

DOX	−	+	+	−	+	+
AP39	−	−	+	−	−	+
CC	−	−	−	+	+	+

GAPDH

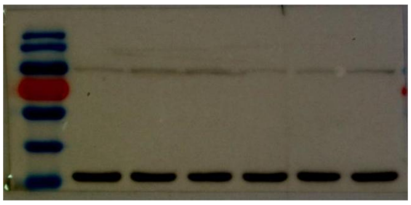

DOX	−	+	+	−	+	+
AP39	−	−	+	−	−	+
CC	−	−	−	+	+	+

Bcl-2+GAPDH

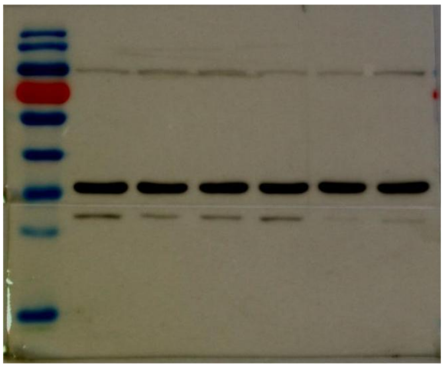

GAPDH
Bcl-2

DOX	−	+	+	−	+	+
AP39	−	−	+	−	−	+
CC	−	−	−	+	+	+

Bcl-2 Repeat 2

Bcl-2

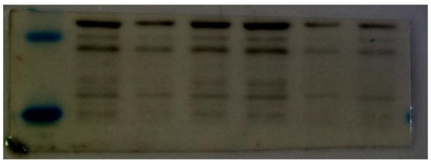

DOX	−	+	+	−	+	+
AP39	−	−	+	−	−	+
CC	−	−	−	+	+	+

GAPDH

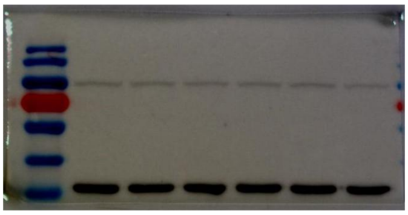

DOX	−	+	+	−	+	+
AP39	−	−	+	−	−	+
CC	−	−	−	+	+	+

Bcl-2+GAPDH

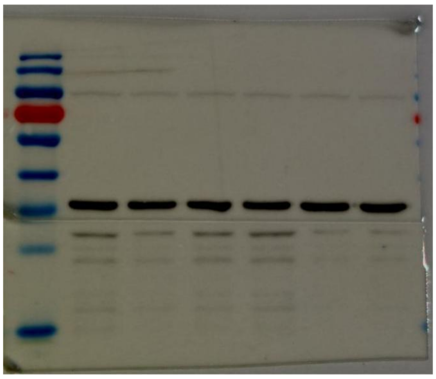

GAPDH
Bcl-2

DOX	−	+	+	−	+	+
AP39	−	−	+	−	−	+
CC	−	−	−	+	+	+

Bcl-2 Repeat 3

Bcl-2

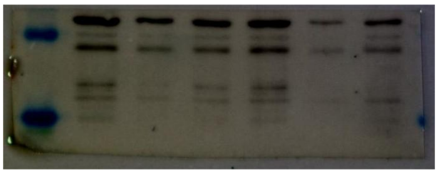

DOX	−	+	+	−	+	+
AP39	−	−	+	−	−	+
CC	−	−	−	+	+	+

GAPDH

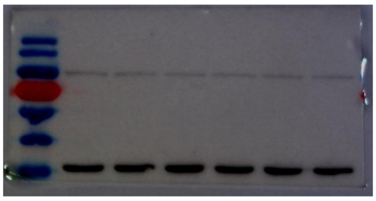

DOX	−	+	+	−	+	+
AP39	−	−	+	−	−	+
CC	−	−	−	+	+	+

Bcl-2+GAPDH

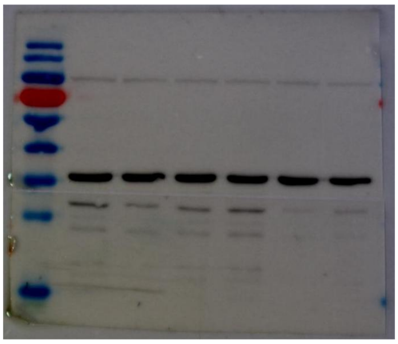

GAPDH
Bcl-2

DOX	−	+	+	−	+	+
AP39	−	−	+	−	−	+
CC	−	−	−	+	+	+

Bcl-2 Repeat 4

Bcl-2

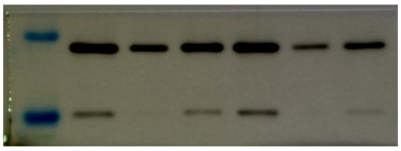

DOX	–	+	+	–	+	+
AP39	–	–	+	–	–	+
CC	–	–	–	+	+	+

GAPDH

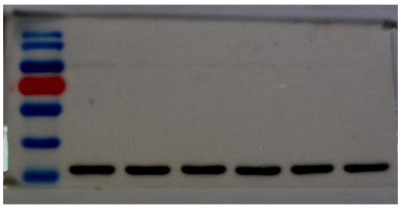

DOX	–	+	+	–	+	+
AP39	–	–	+	–	–	+
CC	–	–	–	+	+	+

Bcl-2+GAPDH

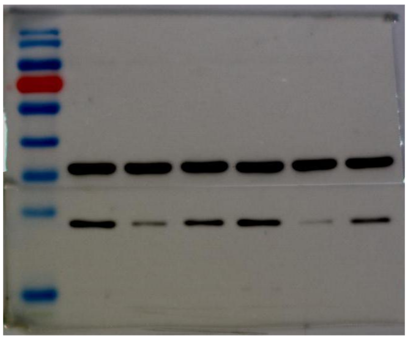

GAPDH

Bcl-2

DOX	–	+	+	–	+	+
AP39	–	–	+	–	–	+
CC	–	–	–	+	+	+

Cleaved Caspase-3 and Caspase-3 Repeat1

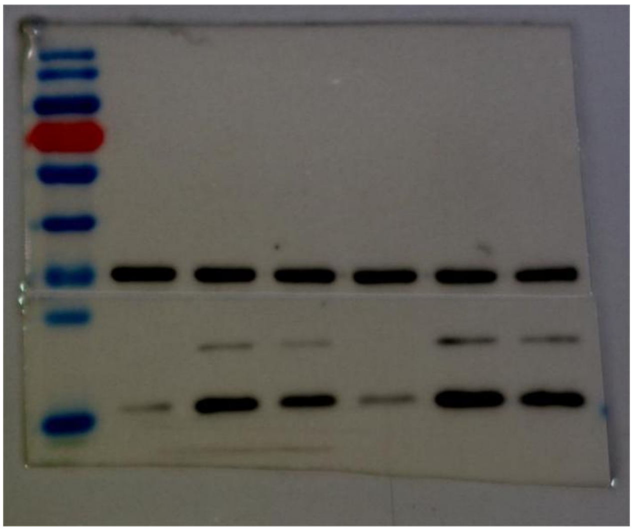

Caspase-3

Cleaved Caspase-3

DOX	–	+	+	–	+	+
AP39	–	–	+	–	–	+
CC	–	–	–	+	+	+

GAPDH

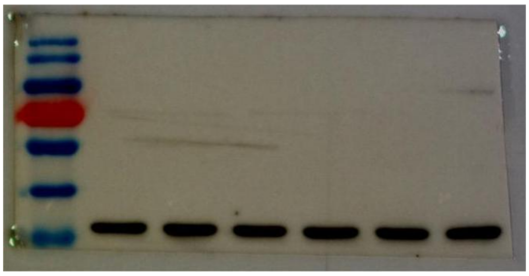

DOX	–	+	+	–	+	+
AP39	–	–	+	–	–	+
CC	–	–	–	+	+	+

Cleaved Caspase-3 and Caspase-3 Repeat 2

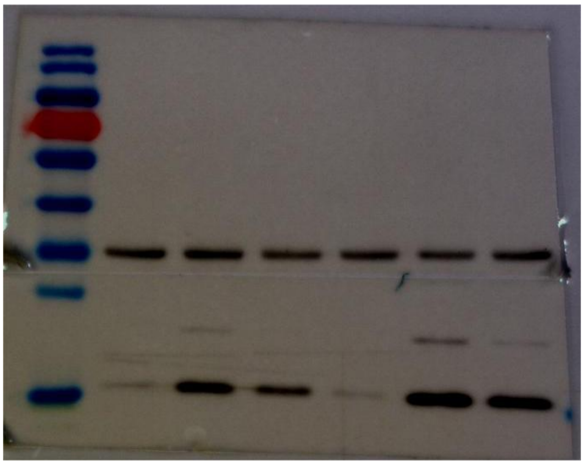

GAPDH

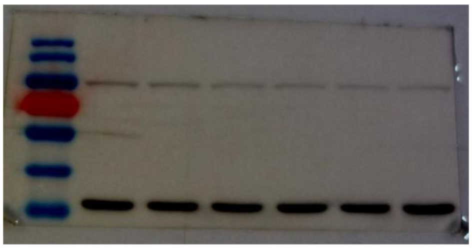

Cleaved Caspase-3 and Caspase-3 Repeat 3

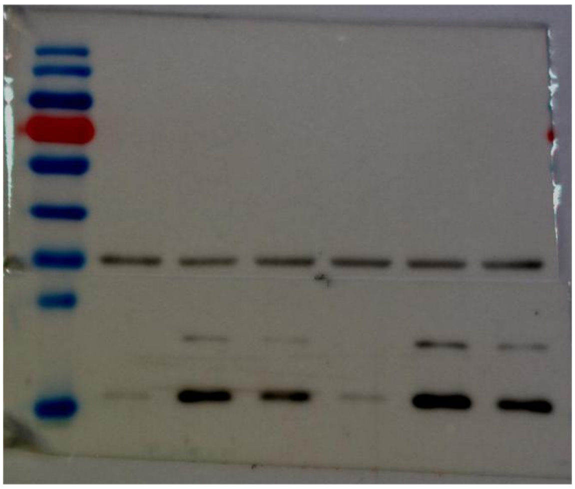

GAPDH

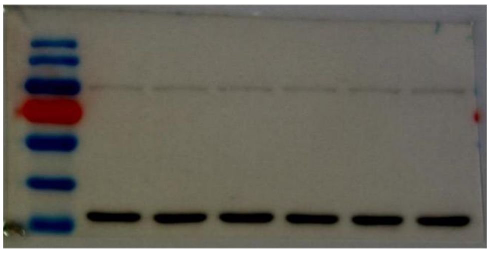

UCP2 Repeat1

UCP2

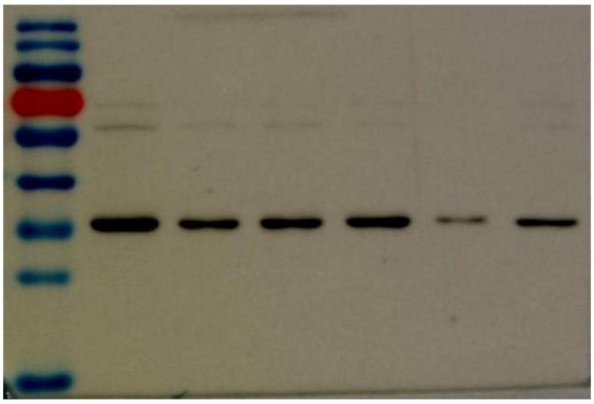

DOX	−	+	+	−	+	+
AP39	−	−	+	−	−	+
CC	−	−	−	+	+	+

GAPDH

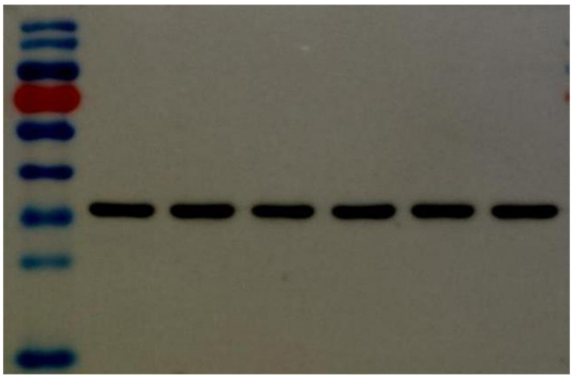

DOX	−	+	+	−	+	+
AP39	−	−	+	−	−	+
CC	−	−	−	+	+	+

UCP2 Repeat 2

UCP2

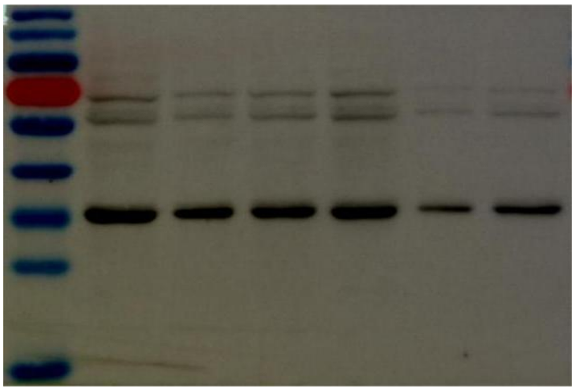

DOX	−	+	+	−	+	+
AP39	−	−	+	−	−	+
CC	−	−	−	+	+	+

GAPDH

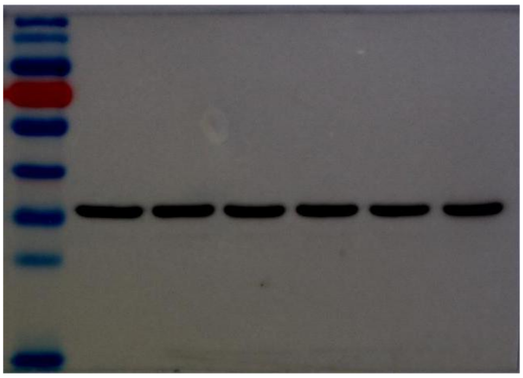

DOX	−	+	+	−	+	+
AP39	−	−	+	−	−	+
CC	−	−	−	+	+	+

UCP2 Repeat 3

UCP2

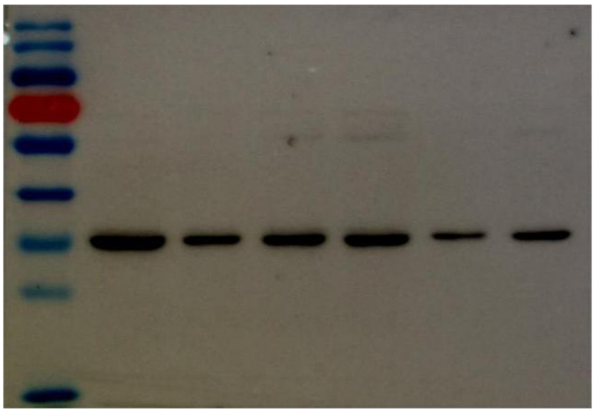

DOX	−	+	+	−	+	+
AP39	−	−	+	−	−	+
CC	−	−	−	+	+	+

GAPDH

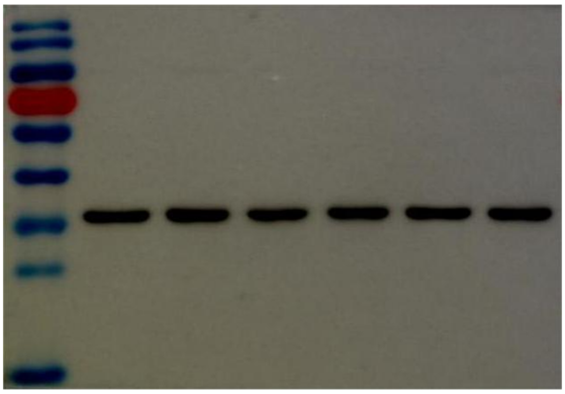

DOX	−	+	+	−	+	+
AP39	−	−	+	−	−	+
CC	−	−	−	+	+	+

FIG.6

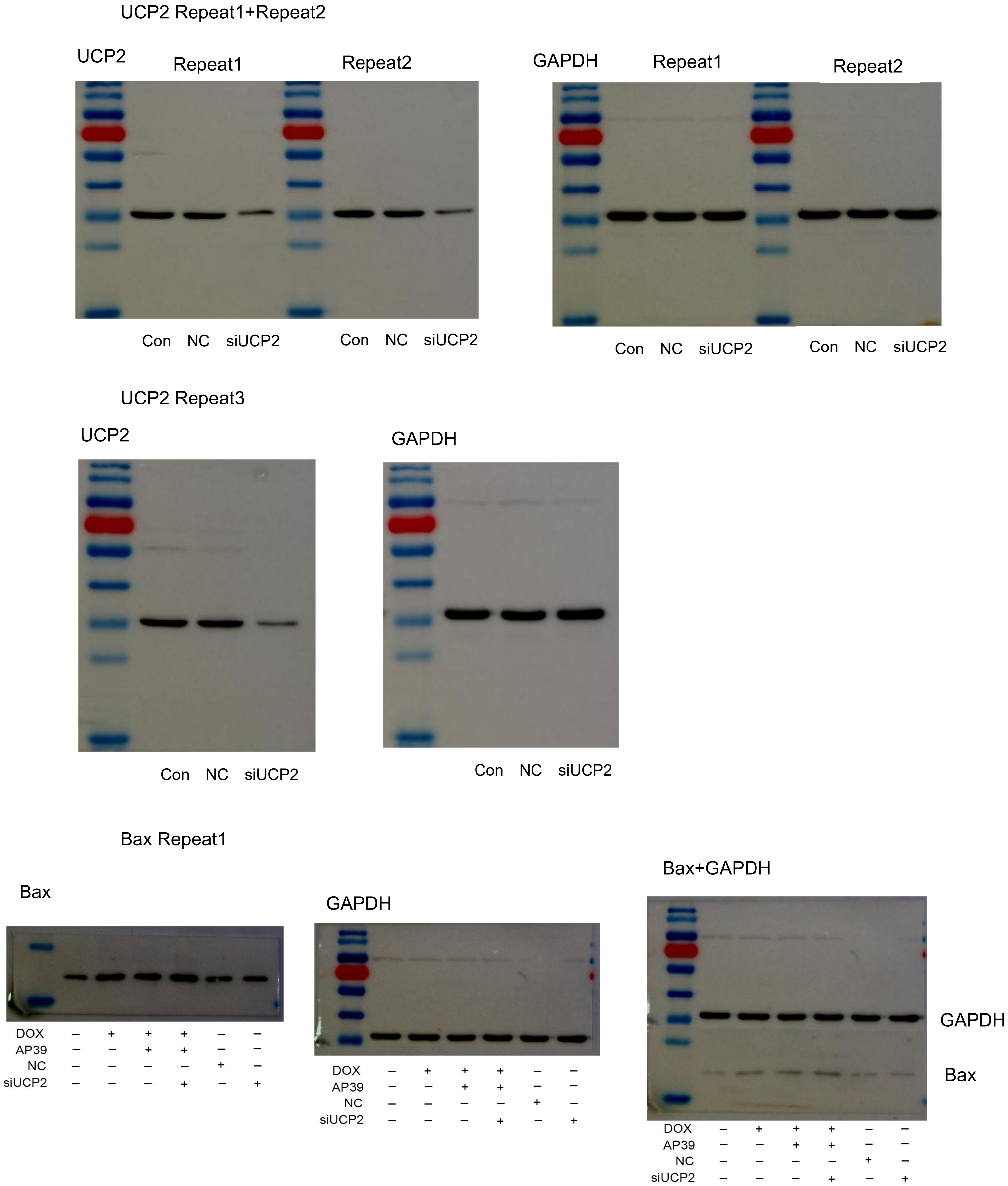
UCP2 Repeat1+Repeat2
UCP2
Repeat1
Repeat2
Con NC siUCP2
Con NC siUCP2
GAPDH
Repeat1
Repeat2
Con NC siUCP2
Con NC siUCP2
UCP2 Repeat3
UCP2
Con NC siUCP2
GAPDH
Con NC siUCP2
Bax Repeat1
Bax
DOX – + + + – –
AP39 – – + + – –
NC – – – – + –
siUCP2 – – – + – +
GAPDH
DOX – + + + – –
AP39 – – + + – –
NC – – – – + –
siUCP2 – – – + – +
Bax+GAPDH
GAPDH
Bax
DOX – + + + – –
AP39 – – + + – –
NC – – – – + –
siUCP2 – – – + – +

Bax Repeat2

Bax

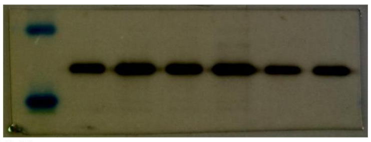

DOX	–	+	+	+	–	–
AP39	–	–	+	+	–	–
NC	–	–	–	–	+	–
siUCP2	–	–	–	+	–	+

GAPDH

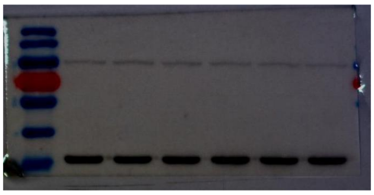

DOX	–	+	+	+	–	–
AP39	–	–	+	+	–	–
NC	–	–	–	–	+	–
siUCP2	–	–	–	+	–	+

Bax+GAPDH

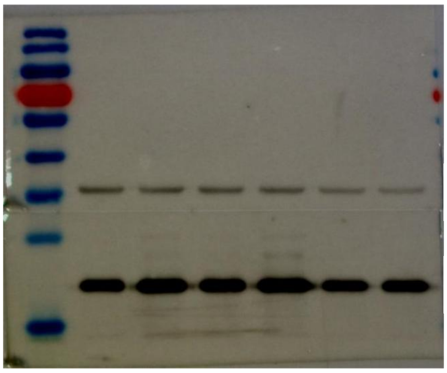

GAPDH

Bax

DOX	–	+	+	+	–	–
AP39	–	–	+	+	–	–
NC	–	–	–	–	+	–
siUCP2	–	–	–	+	–	+

Bax Repeat3

Bax

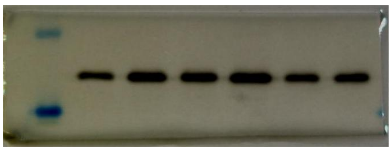

DOX	–	+	+	+	–	–
AP39	–	–	+	+	–	–
NC	–	–	–	–	+	–
siUCP2	–	–	–	+	–	+

GAPDH

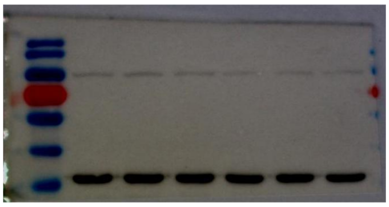

DOX	–	+	+	+	–	–
AP39	–	–	+	+	–	–
NC	–	–	–	–	+	–
siUCP2	–	–	–	+	–	+

Bax+GAPDH

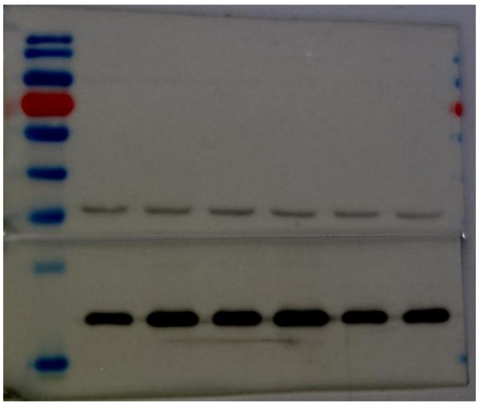

GAPDH

Bax

DOX	–	+	+	+	–	–
AP39	–	–	+	+	–	–
NC	–	–	–	–	+	–
siUCP2	–	–	–	+	–	+

Bax Repeat4

Bax

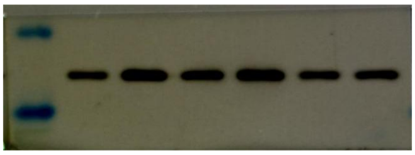

DOX	–	+	+	+	–	–
AP39	–	–	+	+	–	–
NC	–	–	–	–	+	–
siUCP2	–	–	–	+	–	+

GAPDH

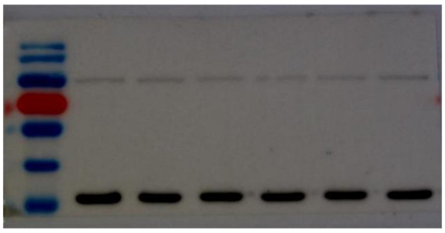

DOX	–	+	+	+	–	–
AP39	–	–	+	+	–	–
NC	–	–	–	–	+	–
siUCP2	–	–	–	+	–	+

Bax+GAPDH

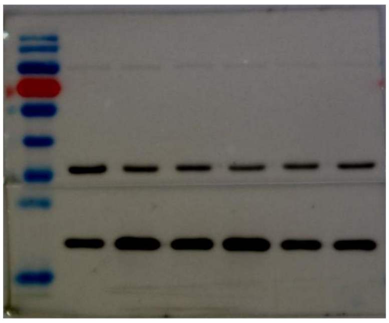

GAPDH

Bax

DOX	–	+	+	+	–	–
AP39	–	–	+	+	–	–
NC	–	–	–	–	+	–
siUCP2	–	–	–	+	–	+

Bcl-2 Repeat1

Bcl-2

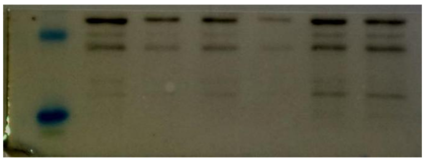

DOX	–	+	+	+	–	–
AP39	–	–	+	+	–	–
NC	–	–	–	–	+	–
siUCP2	–	–	–	+	–	+

GAPDH

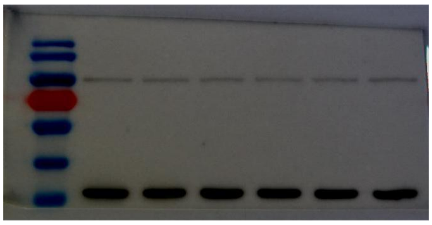

DOX	–	+	+	+	–	–
AP39	–	–	+	+	–	–
NC	–	–	–	–	+	–
siUCP2	–	–	–	+	–	+

Bcl-2+GAPDH

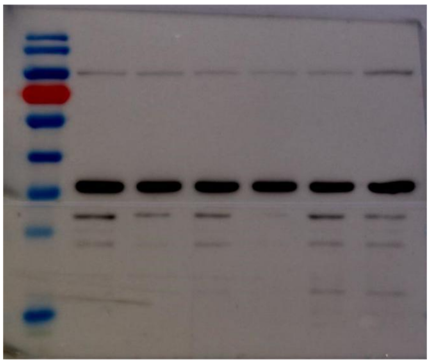

GAPDH

Bcl-2

DOX	–	+	+	+	–	–
AP39	–	–	+	+	–	–
NC	–	–	–	–	+	–
siUCP2	–	–	–	+	–	+

Bcl-2 Repeat2

Bcl-2

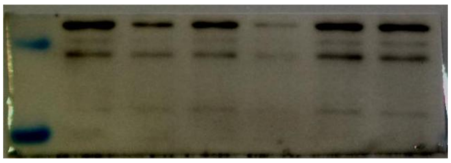

DOX	–	+	+	+	–	–
AP39	–	–	+	+	–	–
NC	–	–	–	–	+	–
siUCP2	–	–	–	+	–	+

GAPDH

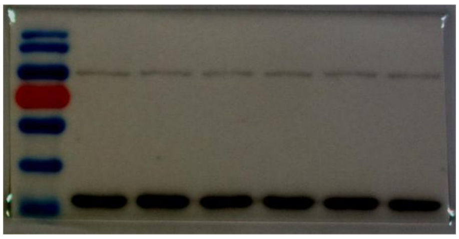

DOX	–	+	+	+	–	–
AP39	–	–	+	+	–	–
NC	–	–	–	–	+	–
siUCP2	–	–	–	+	–	+

Bcl-2+GAPDH

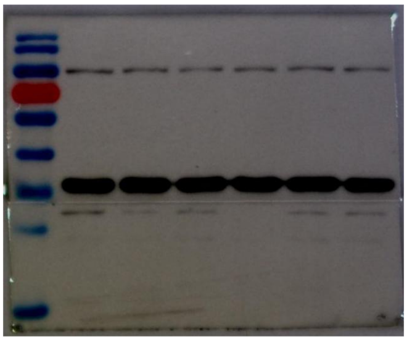

GAPDH

Bcl-2

DOX	–	+	+	+	–	–
AP39	–	–	+	+	–	–
NC	–	–	–	–	+	–
siUCP2	–	–	–	+	–	+

Bcl-2 Repeat3

Bcl-2

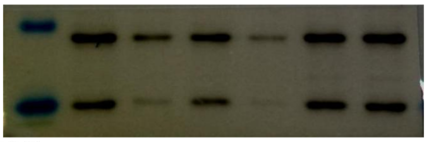

DOX	–	+	+	+	–	–
AP39	–	–	+	+	–	–
NC	–	–	–	–	+	–
siUCP2	–	–	–	+	–	+

GAPDH

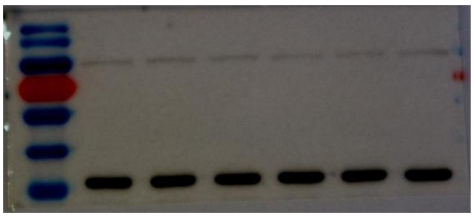

DOX	–	+	+	+	–	–
AP39	–	–	+	+	–	–
NC	–	–	–	–	+	–
siUCP2	–	–	–	+	–	+

Bcl-2+GAPDH

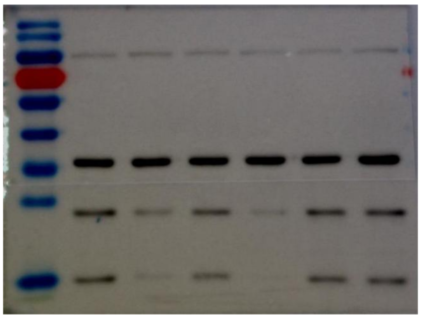

GAPDH

Bcl-2

DOX	–	+	+	+	–	–
AP39	–	–	+	+	–	–
NC	–	–	–	–	+	–
siUCP2	–	–	–	+	–	+

Cleaved Caspase-3 and Caspase-3 Repeat 1

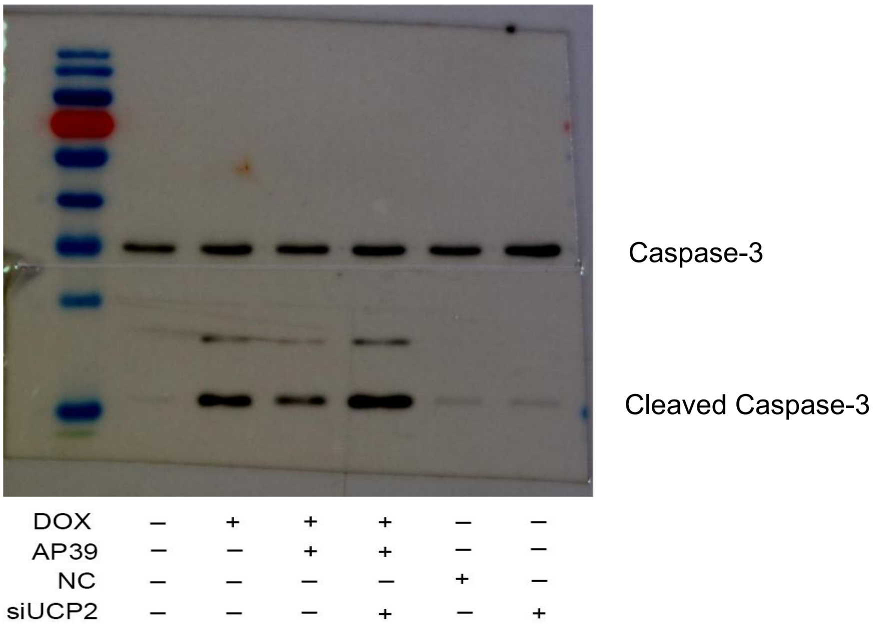

GAPDH

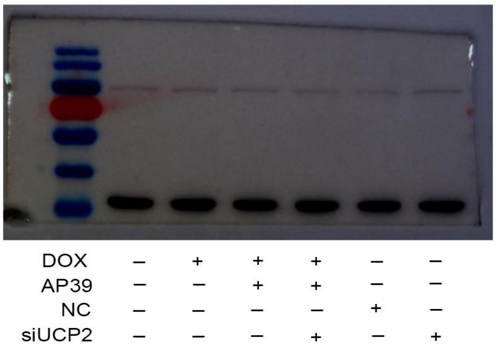

Cleaved Caspase-3 and Caspase-3 Repeat 2

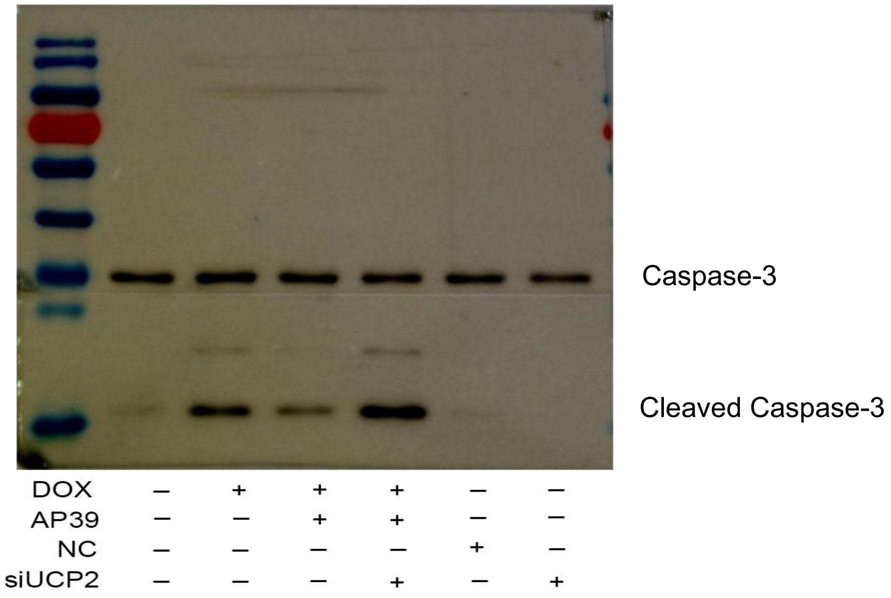

GAPDH

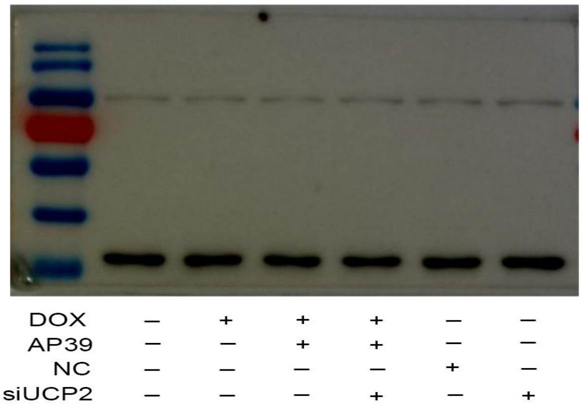

Cleaved Caspase-3 and Caspase-3 Repeat 3

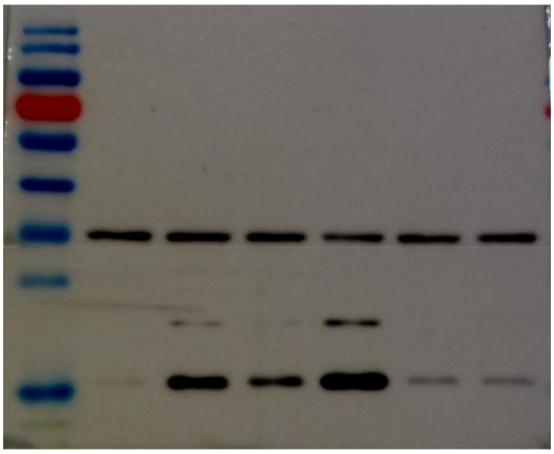

Caspase-3

Cleaved Caspase-3

DOX	–	+	+	+	–	–
AP39	–	–	+	+	–	–
NC	–	–	–	–	+	–
siUCP2	–	–	–	+	–	+

GAPDH

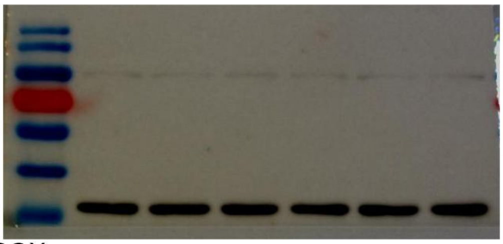

DOX	–	+	+	+	–	–
AP39	–	–	+	+	–	–
NC	–	–	–	–	+	–
siUCP2	–	–	–	+	–	+

p-AMPK and AMPK Repeat1

AMPK

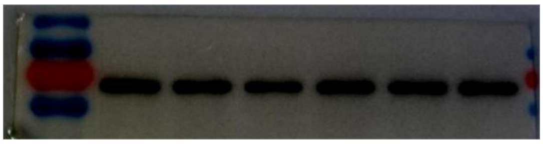

DOX	–	+	+	+	–	–
AP39	–	–	+	+	–	–
NC	–	–	–	–	+	–
siUCP2	–	–	–	+	–	+

AMPK+GAPDH

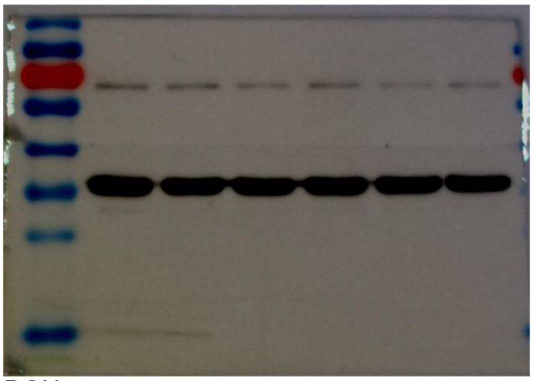

AMPK

GAPDH

DOX	–	+	+	+	–	–
AP39	–	–	+	+	–	–
NC	–	–	–	–	+	–
siUCP2	–	–	–	+	–	+

P-AMPK

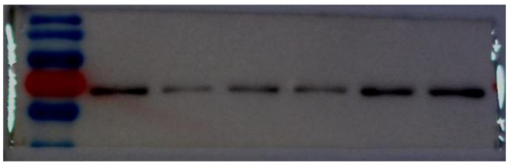

DOX	–	+	+	+	–	–
AP39	–	–	+	+	–	–
NC	–	–	–	–	+	–
siUCP2	–	–	–	+	–	+

P-AMPK+GAPDH

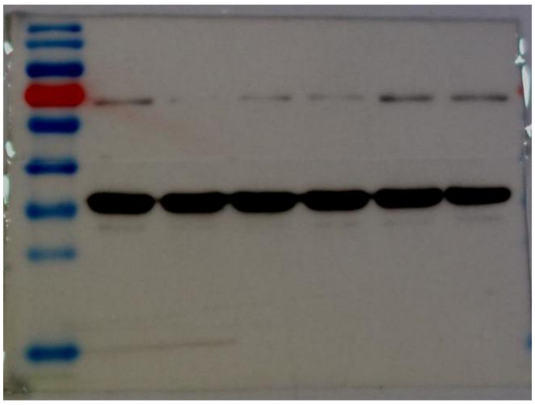

P-AMPK

GAPDH

DOX	–	+	+	+	–	–
AP39	–	–	+	+	–	–
NC	–	–	–	–	+	–
siUCP2	–	–	–	+	–	+

p-AMPK and AMPK Repeat2

AMPK

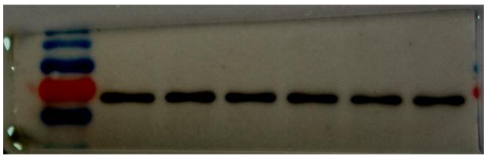

DOX	–	+	+	+	–	–
AP39	–	–	+	+	–	–
NC	–	–	–	–	+	–
siUCP2	–	–	–	+	–	+

AMPK+GAPDH

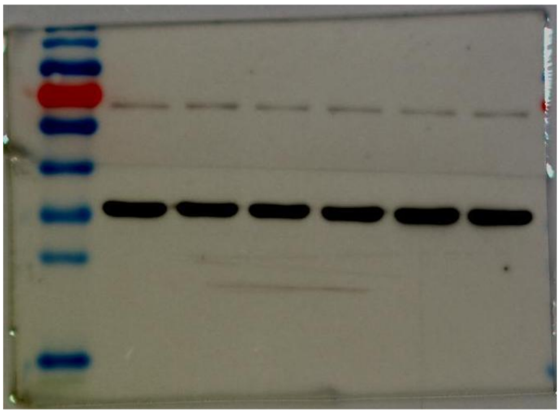

AMPK

GAPDH

DOX	–	+	+	+	–	–
AP39	–	–	+	+	–	–
NC	–	–	–	–	+	–
siUCP2	–	–	–	+	–	+

P-AMPK

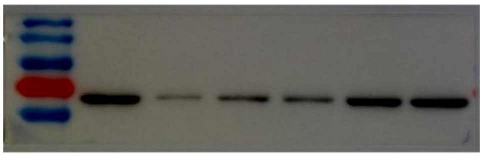

DOX	–	+	+	+	–	–
AP39	–	–	+	+	–	–
NC	–	–	–	–	+	–
siUCP2	–	–	–	+	–	+

P-AMPK+GAPDH

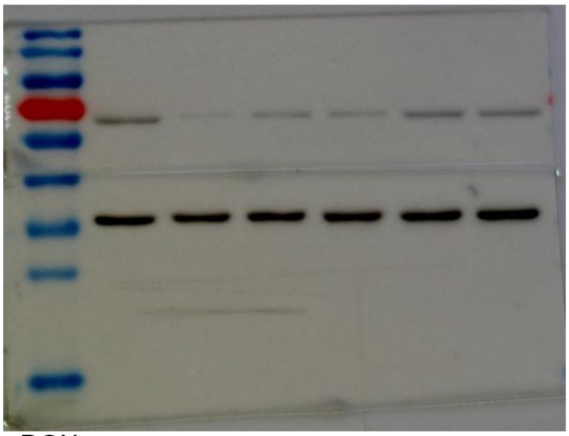

P-AMPK

GAPDH

DOX	–	+	+	+	–	–
AP39	–	–	+	+	–	–
NC	–	–	–	–	+	–
siUCP2	–	–	–	+	–	+

p-AMPK and AMPK Repeat3

AMPK

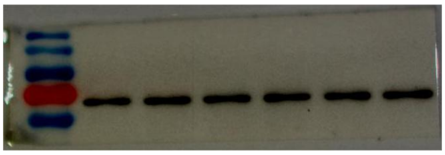

DOX	–	+	+	+	–	–
AP39	–	–	+	+	–	–
NC	–	–	–	–	+	–
siUCP2	–	–	–	+	–	+

AMPK+GAPDH

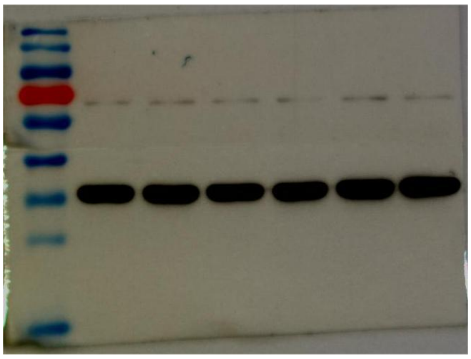

AMPK

GAPDH

DOX	–	+	+	+	–	–
AP39	–	–	+	+	–	–
NC	–	–	–	–	+	–
siUCP2	–	–	–	+	–	+

P-AMPK

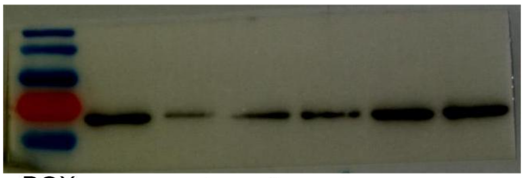

P-AMPK+GAPDH

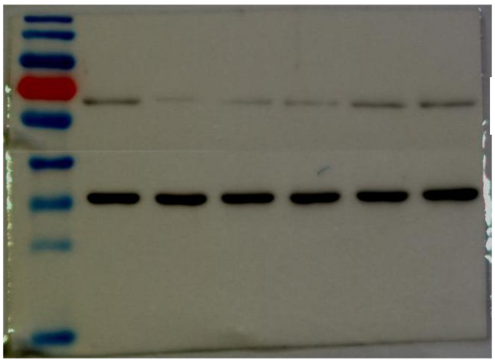

FIG.7

p-AMPK and AMPK Repeat1+Repeat2

AMPK

Repeat1 Repeat2

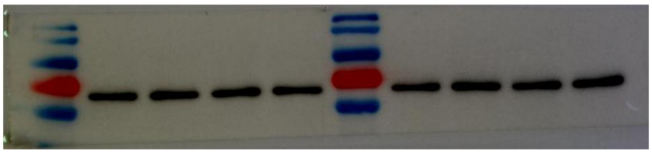

AMPK+GAPDH

Repeat1 Repeat2

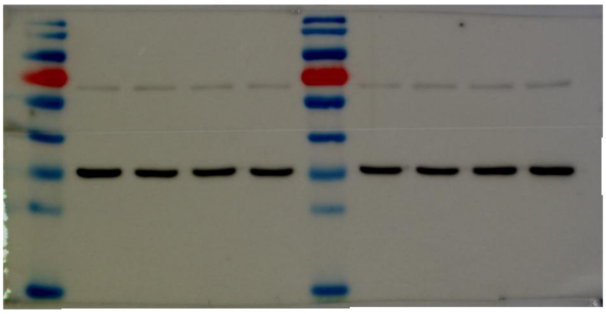

P-AMPK

Repeat1 Repeat2

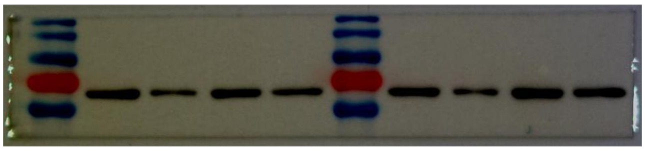

P-AMPK+GAPDH

Repeat1 Repeat2

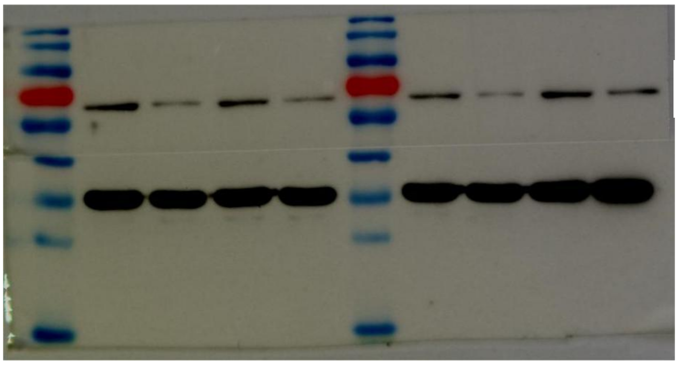

p-AMPK and AMPK Repeat3+Repeat4

AMPK

Repeat3 Repeat4

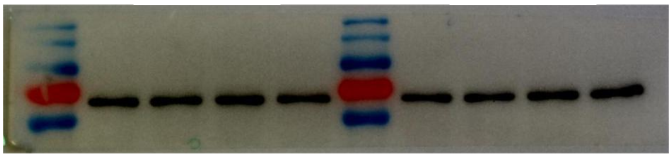

Con DOX AP39 DOX+AP39 Con DOX AP39 DOX+AP39

AMPK+GAPDH

Repeat3 Repeat4

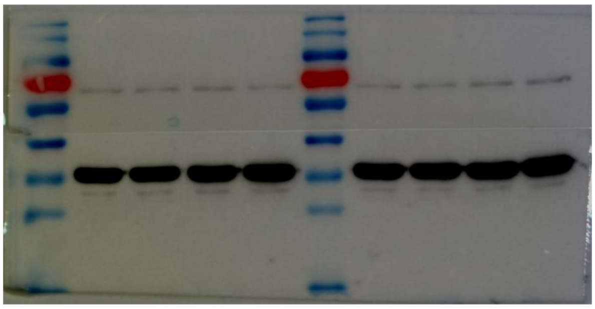

AMPK

GAPDH

Con DOX AP39 DOX+AP39 Con DOX AP39 DOX+AP39

P-AMPK

Repeat3 Repeat4

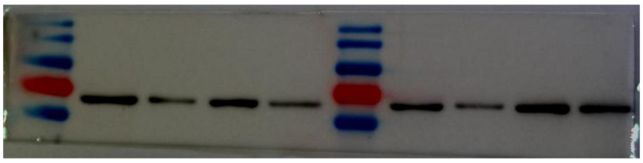

Con DOX AP39 DOX+AP39 Con DOX AP39 DOX+AP39

P-AMPK+GAPDH

Repeat3 Repeat4

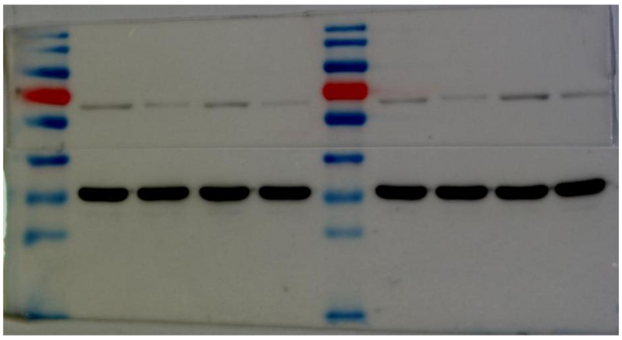

P-AMPK

GAPDH

Con DOX AP39 DOX+AP39 Con DOX AP39 DOX+AP39

UCP2 Repeat1+Repeat2

UCP2

Repeat1 Repeat2

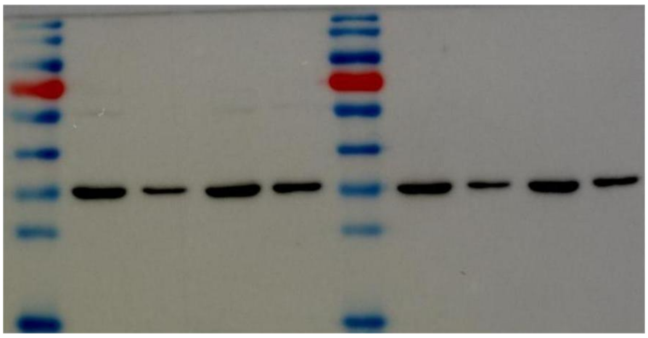

Con DOX AP39 DOX+AP39 Con DOX AP39 DOX+AP39

GAPDH

Repeat1 Repeat2

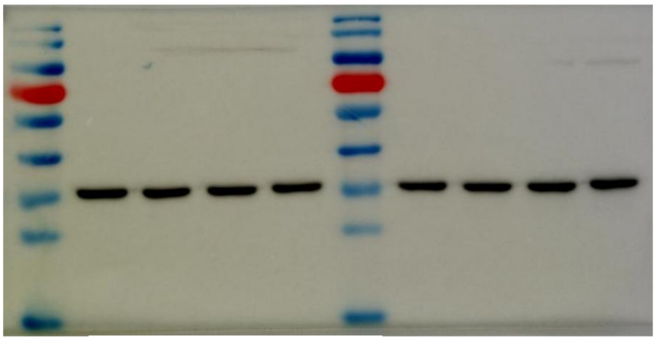

Con DOX AP39 DOX+AP39 Con DOX AP39 DOX+AP39

UCP2 Repeat3+Repeat4

UCP2

Repeat3 Repeat4

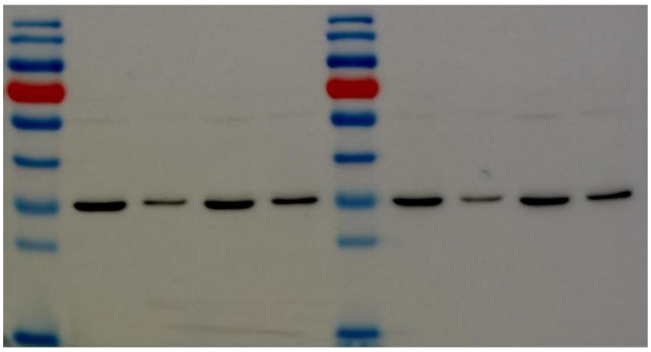

Con DOX AP39 DOX+AP39 Con DOX AP39 DOX+AP39

GAPDH

Repeat3 Repeat4

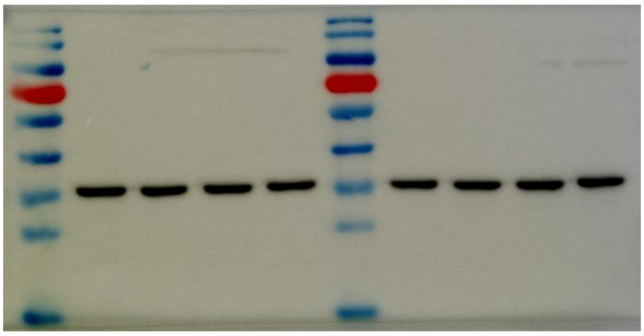

Con DOX AP39 DOX+AP39 Con DOX AP39 DOX+AP39

Bax Repeat1

Bax

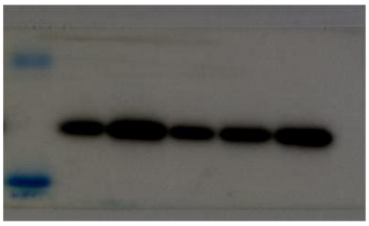

DOX	−	+	−	+	+
AP39	−	−	+	+	+
CC	−	−	−	−	+

GAPDH

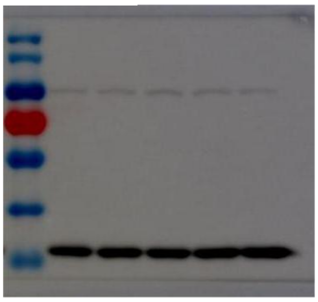

DOX	−	+	−	+	+
AP39	−	−	+	+	+
CC	−	−	−	−	+

Bax+GAPDH

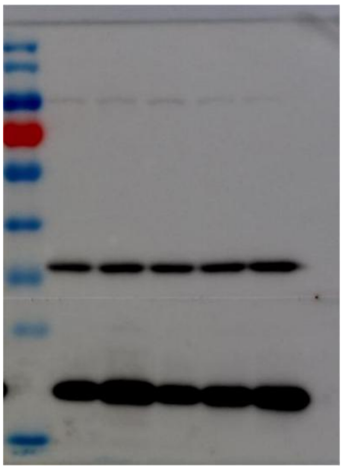

DOX	−	+	−	+	+
AP39	−	−	+	+	+
CC	−	−	−	−	+

Bax Repeat2

Bax

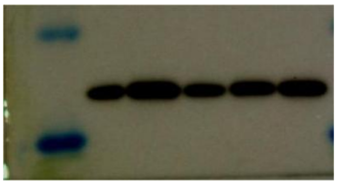

DOX	−	+	−	+	+
AP39	−	−	+	+	+
CC	−	−	−	−	+

GAPDH

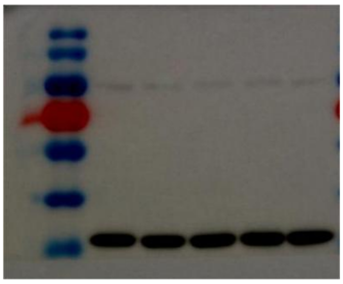

DOX	−	+	−	+	+
AP39	−	−	+	+	+
CC	−	−	−	−	+

Bax+GAPDH

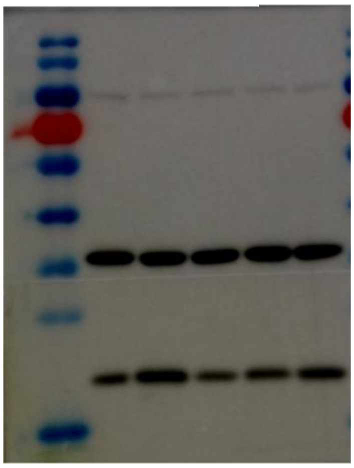

DOX	−	+	−	+	+
AP39	−	−	+	+	+
CC	−	−	−	−	+

Bax Repeat3

Bax

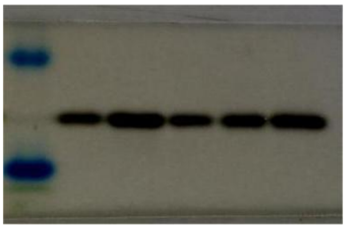

DOX	−	+	−	+	+
AP39	−	−	+	+	+
CC	−	−	−	−	+

GAPDH

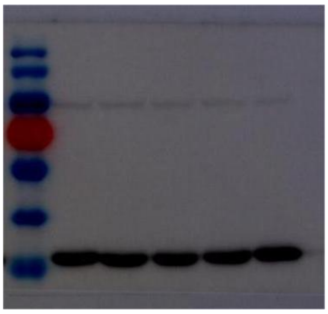

DOX	−	+	−	+	+
AP39	−	−	+	+	+
CC	−	−	−	−	+

Bax+GAPDH

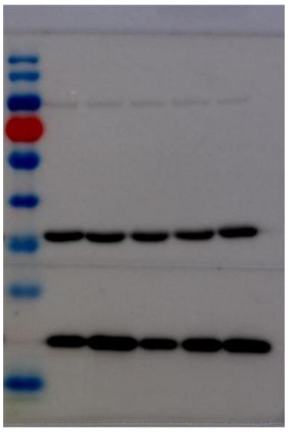

DOX	−	+	−	+	+
AP39	−	−	+	+	+
CC	−	−	−	−	+

Bcl-2 Repeat1+Repeat2

Bcl-2 Repeat1 Repeat2

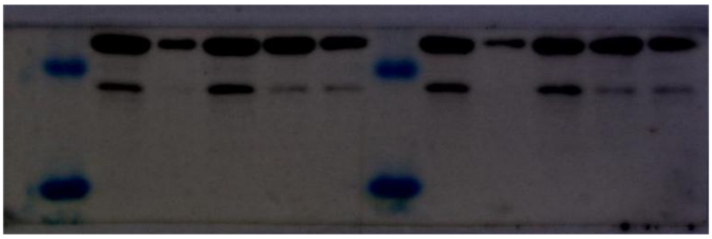

DOX	−	+	−	+	+	DOX	−	+	−	+	+
AP39	−	−	+	+	+	AP39	−	−	+	+	+
CC	−	−	−	−	+	CC	−	−	−	−	+

GAPDH Repeat1 Repeat2

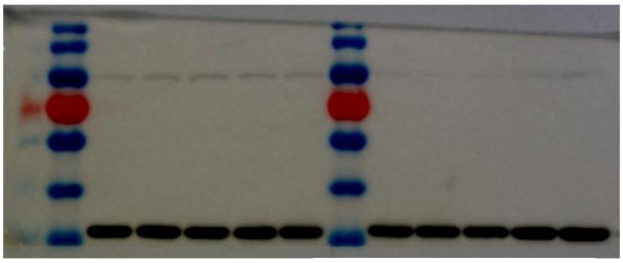

DOX	−	+	−	+	+	DOX	−	+	−	+	+
AP39	−	−	+	+	+	AP39	−	−	+	+	+
CC	−	−	−	−	+	CC	−	−	−	−	+

Bcl-2+GAPDH Repeat1 Repeat2

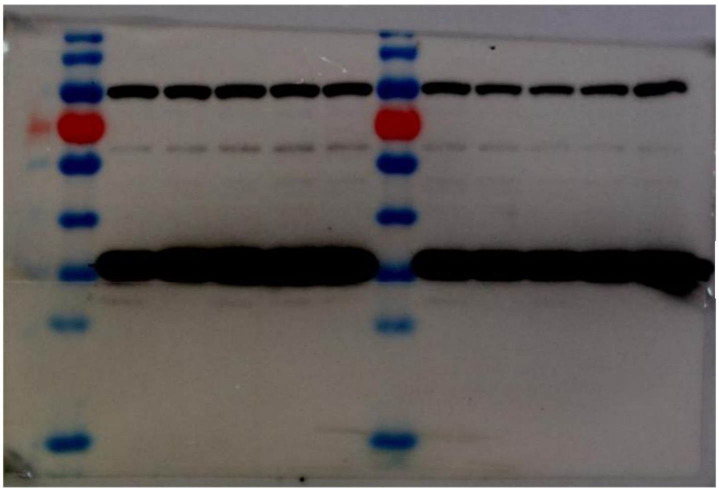

DOX	−	+	−	+	+	DOX	−	+	−	+	+
AP39	−	−	+	+	+	AP39	−	−	+	+	+
CC	−	−	−	−	+	CC	−	−	−	−	+

Bcl-2 Repeat3

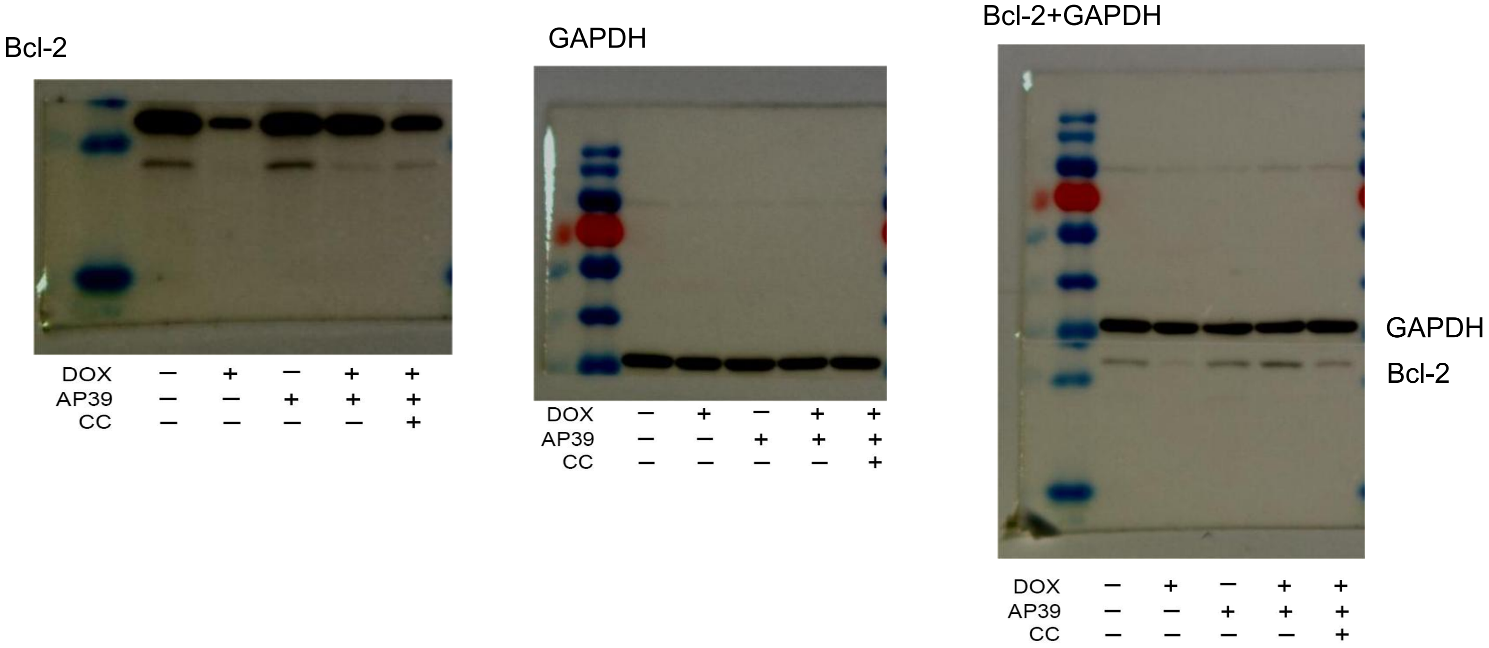

Bcl-2 Repeat4

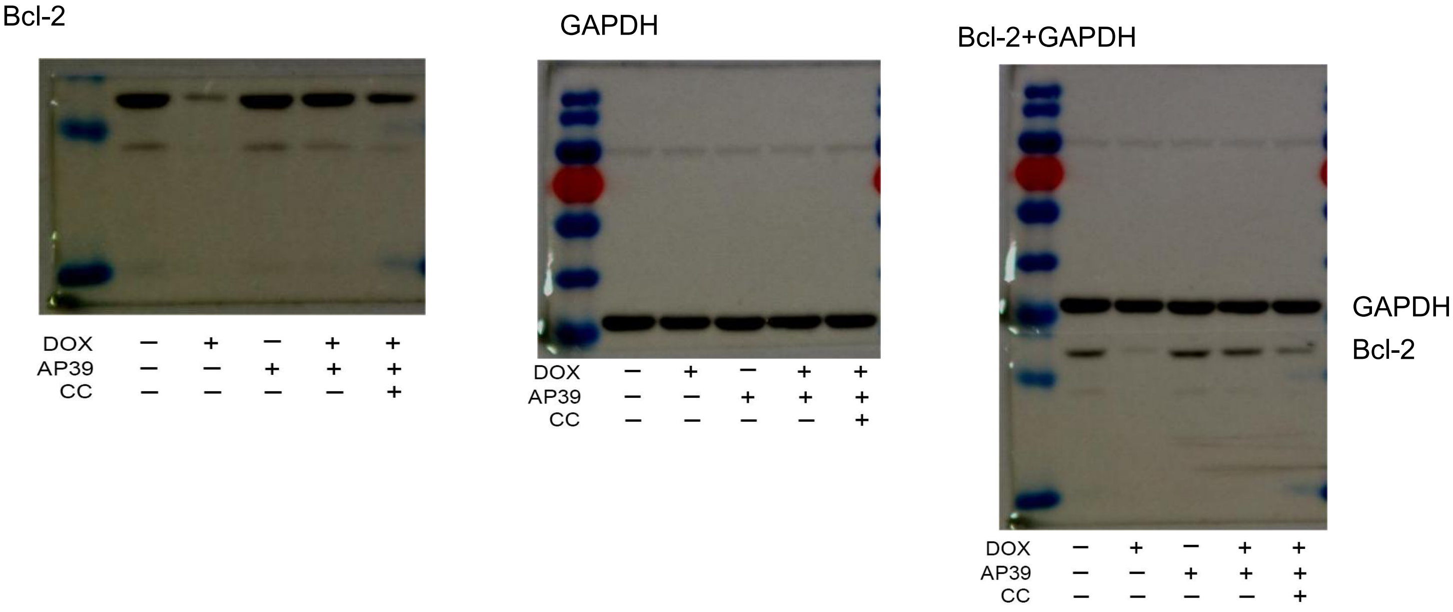

Cleaved Caspase-3 and Caspase-3 Repeat 1+Repeat2

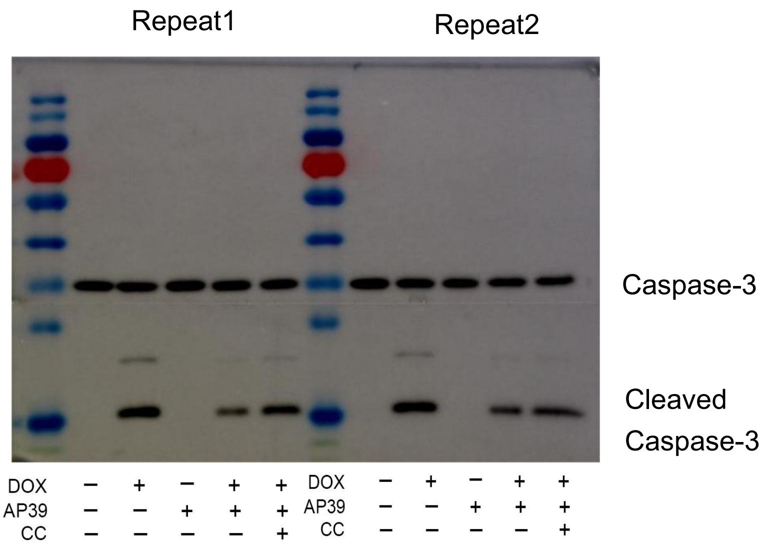

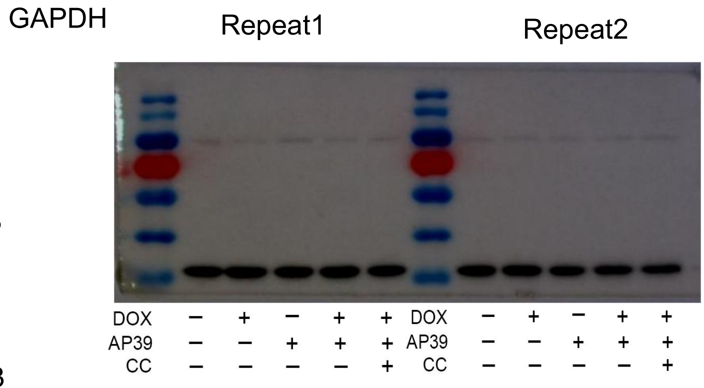

Cleaved Caspase-3 and Caspase-3 Repeat3

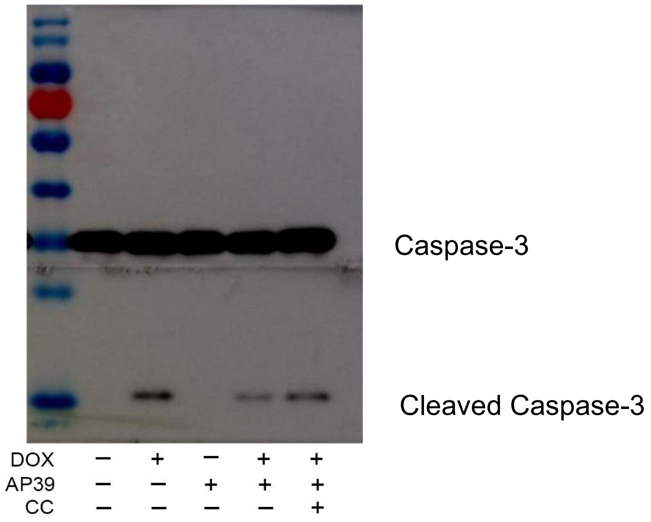

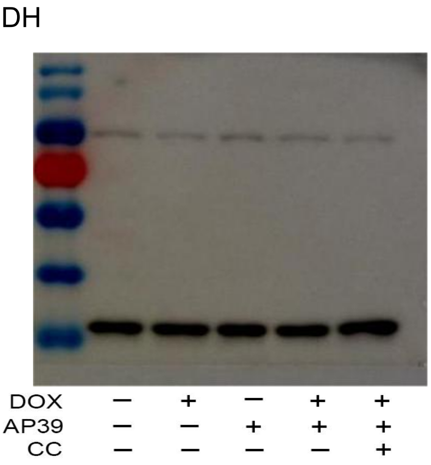

Cleaved Caspase-3 and Caspase-3 Repeat4

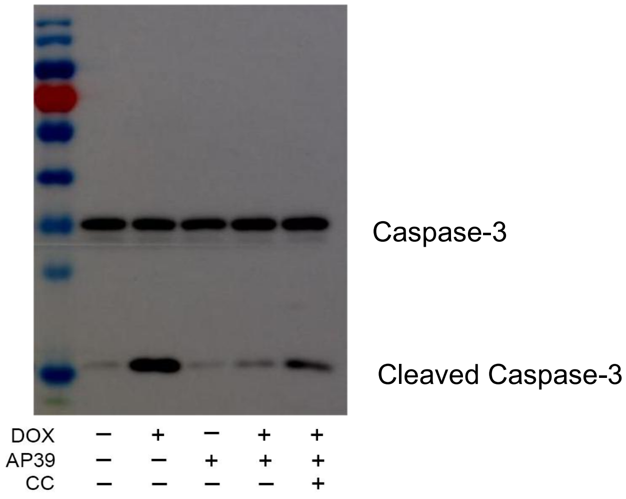

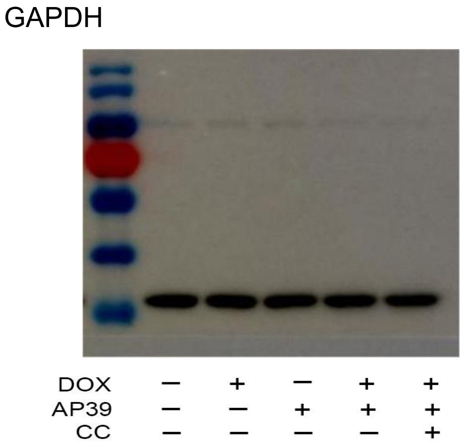

Bax Repeat1

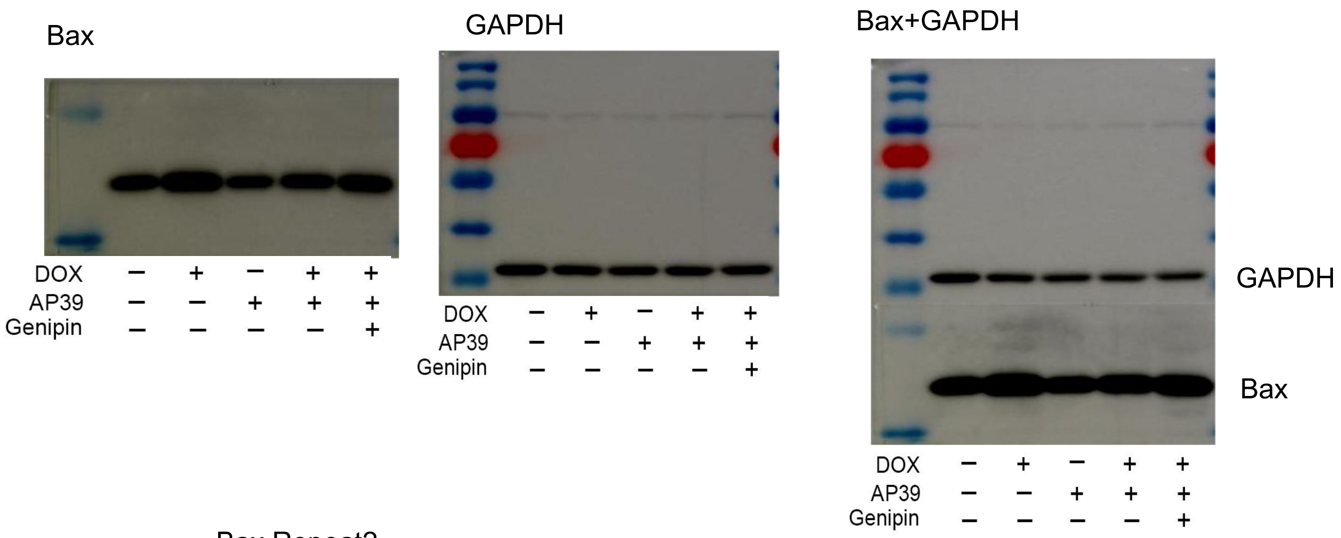

Bax Repeat2

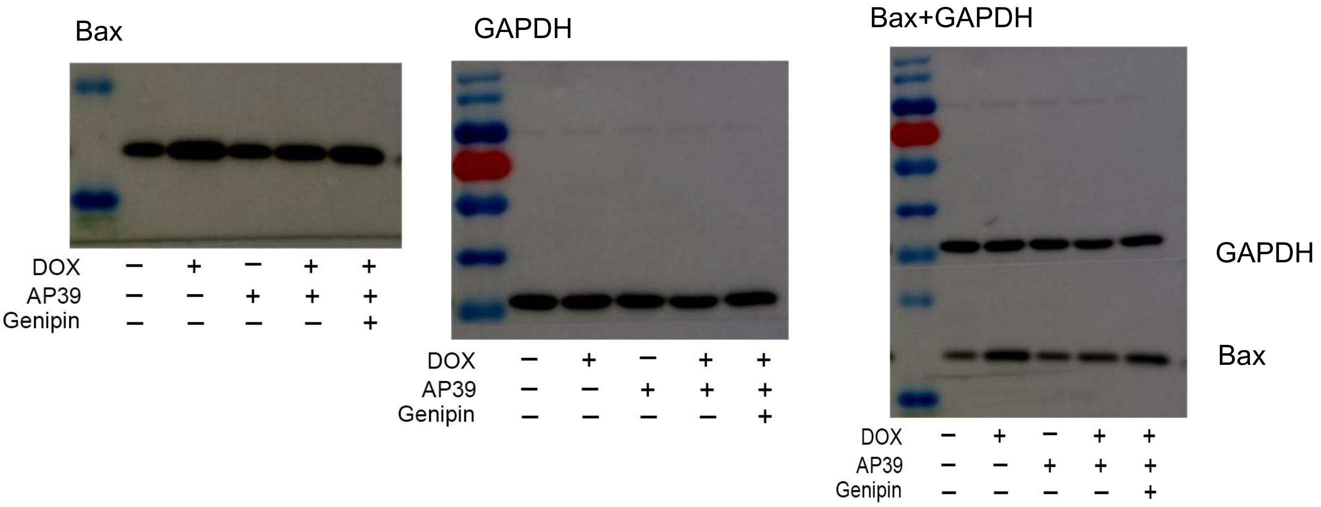

Bax Repeat3

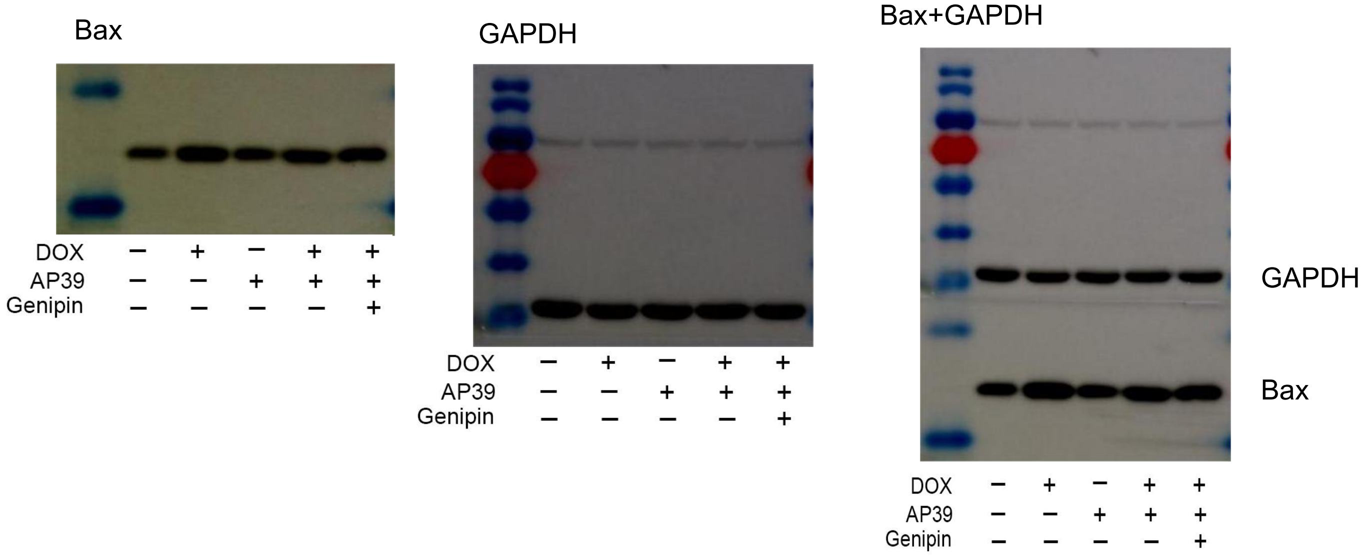

Bcl-2 Repeat1+Repeat2

Bcl-2 Repeat1 Repeat2

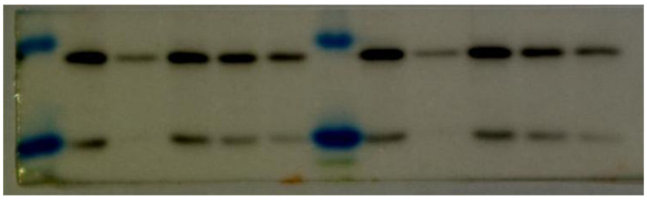

GAPDH Repeat1 Repeat2

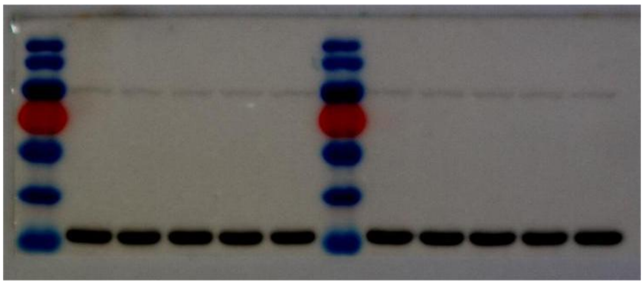

Bcl-2+GAPDH Repeat1 Repeat2

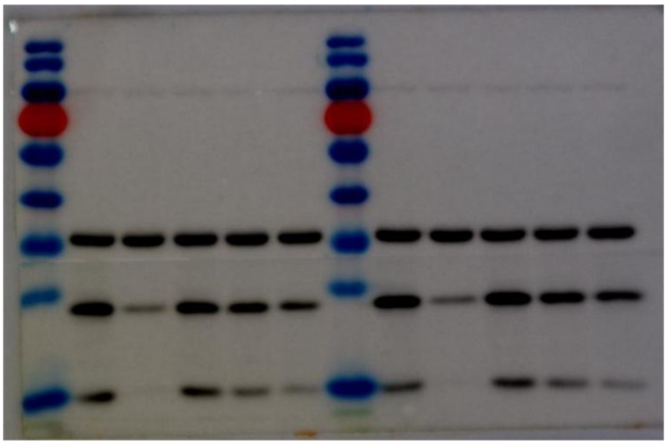

Bcl-2 Repeat3

Bcl-2

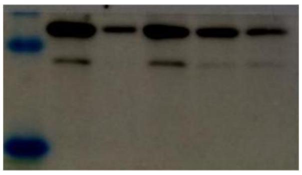

GAPDH

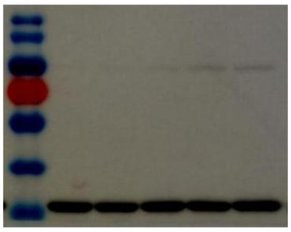

Bcl-2+GAPDH

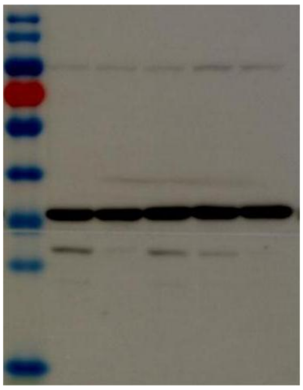

Bcl-2 Repeat4

Bcl-2

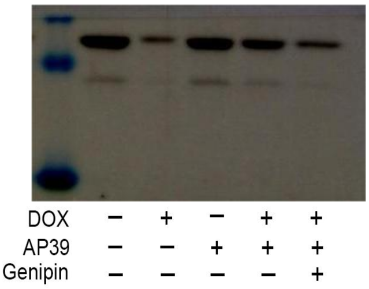

GAPDH

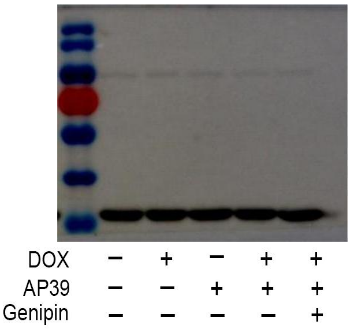

Bcl-2+GAPDH

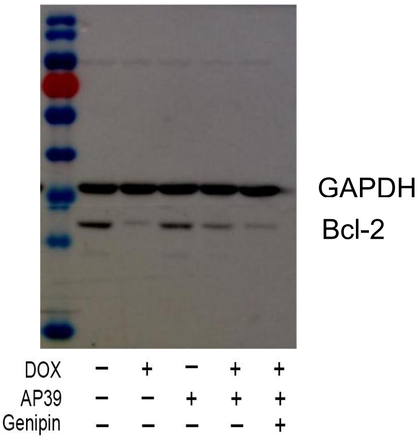

Cleaved Caspase-3 and Caspase-3 Repeat 1+Repeat2

Repeat1 Repeat2

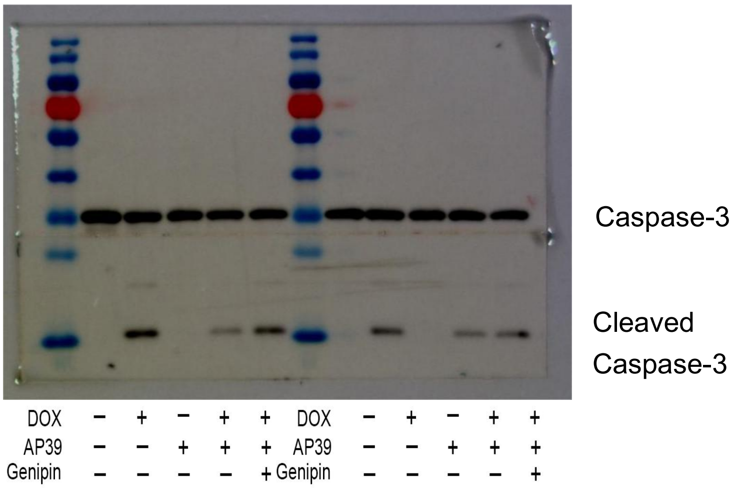

GAPDH Repeat1 Repeat2

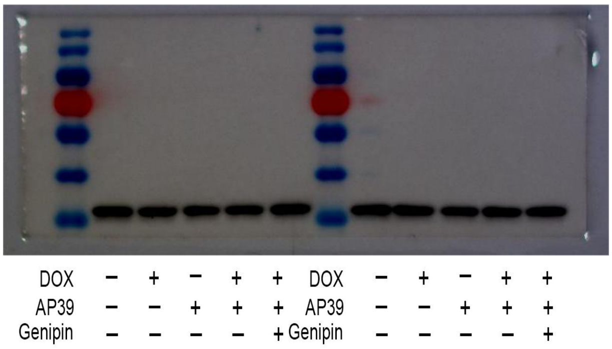

Cleaved Caspase-3 and Caspase-3 Repeat 3

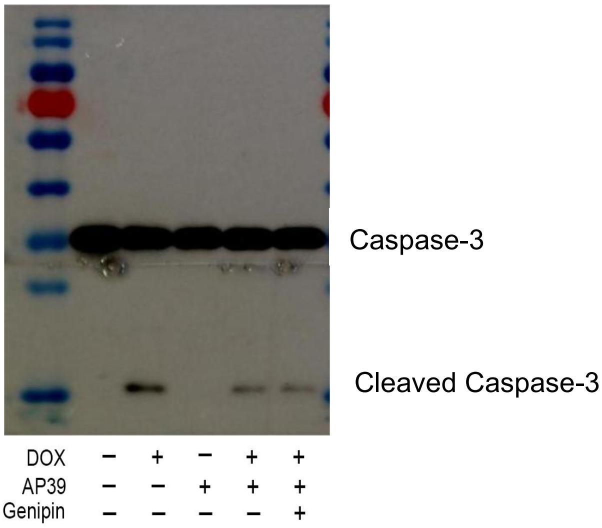

GAPDH

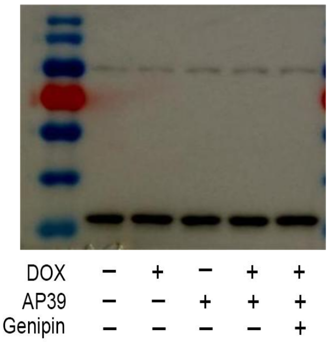

Cleaved Caspase-3 and Caspase-3 Repeat 4

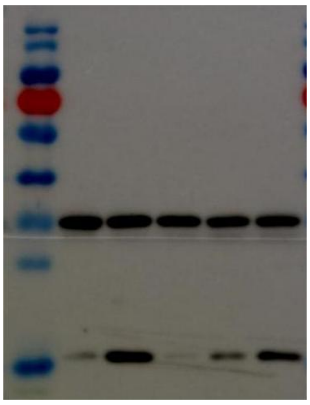

GAPDH

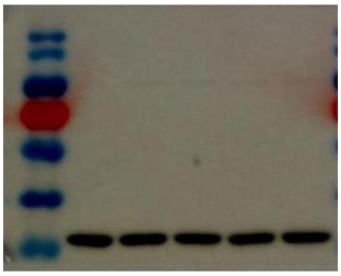

Supplement: S1 Raw images — (PDF) [file pone.0300261.s001.pdf]
