# Supplementary material for: AP39, a novel mitochondria-targeted hydrogen sulfide donor ameliorates doxorubicin-induced cardiotoxicity by regulating the AMPK/UCP2 pathway
Source: PLoS One. 2024 Apr 3;19(4):e0300261. doi: 10.1371/journal.pone.0300261 (PMC10990198; doi:10.1371/journal.pone.0300261)
Supplement: S1 Raw data — (PDF) [file pone.0300261.s002.pdf]

Fig 1

A

Con	0.5	1	2
95.02034413	65.14388397	55.14037278	44.41718490
104.0563140	69.34688853	57.51302475	48.55017626
95.78485244	65.34122742	53.20755542	41.90250464
105.1384894	72.65726432	31.47095677	48.76880288

描述性统计	A	B	C	D
	Con	0.5	1	2
值的数目	4	4	4	4
最小	95.02	65.14	53.21	41.90
最大值	105.1	72.66	61.47	48.77
范围	10.12	7.513	8.263	6.866
平均: mean	100.0	68.12	56.83	45.91
标准偏差 SD	5.336	3.590	3.558	3.338
标准误差的平均值	2.668	1.795	1.779	1.669

B

组 A	组 B	组 C	组 D	组 E
Con	6h	12h	24h	48h
99.17768722	35.31934035	33.22778282	48.72804625	20.66744672
97.08317187	30.87260331	79.05687465	47.57787506	20.21245819
100.27804490	34.25175350	31.94047186	50.73913218	19.16341642
96.97208912	79.16345023	78.35253681	48.80194492	19.23327742
106.48900690	38.19340679	32.16634636	56.67656583	22.25307541

描述性统计	A	B	C	D	E
	Con	6h	12h	24h	48h
值的数目	5	5	5	5	5
最小	96.97	79.16	78.35	47.58	19.16
最大值	106.5	88.19	83.23	56.68	22.25
范围	9.517	9.030	4.875	9.099	3.090
平均: mean	100.0	83.56	80.95	50.50	20.31
标准偏差 SD	3.890	3.591	2.120	3.632	1.264
标准误差的平均值	1.740	1.606	0.9481	1.624	0.5651

C

组 A	组 B
Con	DOX
1.015931013	2.207449008
0.860943832	2.097909195
0.997737029	2.337513439
1.045590214	2.143055837
1.079797911	2.134597890

描述性统计	A	B
	Con	DOX
值的数目	5	5
最小	0.8609	2.098
最大值	1.080	2.338
范围	0.2189	0.2396
平均: mean	1.000	2.184
标准偏差 SD	0.08373	0.09440
标准误差的平均值	0.03744	0.04222

D

SOD

格式: 列	组 A	组 B
	Con	DOX
1	0.009190838	0.004009002
2	0.008710496	0.004072746
3	0.008839669	0.003650656
4	0.009539399	0.003701586

描述性统计	A	B
	Con	DOX
值的数目	4	4
最小	0.008710	0.003651
最大值	0.009539	0.004073
范围	0.0008289	0.0004221
平均: mean	0.009070	0.003858
标准偏差 SD	0.0003729	0.0002132
标准误差的平均值	0.0001865	0.0001066

GSH-Px

格式: 列	组 A	组 B
	Con	DOX
1	0.309686445	0.107101366
2	0.338544448	0.148452906
3	0.359813874	0.135419239
4	0.379903288	0.141234147

描述性统计	A	B
	Con	DOX
值的数目	4	4
最小	0.3097	0.1071
最大值	0.3799	0.1485
范围	0.07022	0.04135
平均: mean	0.3470	0.1331
标准偏差 SD	0.03006	0.01810
标准误差的平均值	0.01503	0.009052

MDA

格式: 列	组 A	组 B
	Con	DOX
1	0.004240398	0.008198603
2	0.004653456	0.007523505
3	0.003994748	0.007390016
4	0.004438287	0.006963769

描述性统计	A	B
	Con	DOX
值的数目	4	4
最小	0.003995	0.006964
最大值	0.004653	0.008199
范围	0.0006587	0.001235
平均: mean	0.004332	0.007519
标准偏差 SD	0.0002809	0.0005121
标准误差的平均值	0.0001405	0.0002561

NADPH

格式: 列	组 A	组 B
	Con	DOX
1	0.000413339	0.002508246
2	0.000419321	0.002661953
3	0.000405097	0.002226949
4	0.000450453	0.002327293

描述性统计	A	B
	Con	DOX
值的数目	4	4
最小	0.0004051	0.002227
最大值	0.0004505	0.002662
范围	4.536e-005	0.0004350
平均: mean	0.0004221	0.002431
标准偏差 SD	1.981e-005	0.0001930
标准误差的平均值	9.906e-006	9.648e-005

E

格式: 列	组 A Con	组 B DOX
1	4.01	25.41
2	4.58	32.06
3	5.02	32.48

描述性统计	A Con	B DOX
值的数目	3	3
最小	4.010	25.41
最大值	5.020	32.48
范围	1.010	7.070
平均: mean	4.537	29.98
标准偏差 SD	0.5064	3.966
标准误差的平均值	0.2924	2.290

F

格式: 列	组 A Con	组 B DOX
1	7	33.00
2	8	28.00
3	2	26.00

描述性统计	A Con	B DOX
值的数目	3	3
最小	2.000	26.00
最大值	8.000	33.00
范围	6.000	7.000
平均: mean	5.667	29.00
标准偏差 SD	3.215	3.606
标准误差的平均值	1.856	2.082

Fig 2

A

Bax

格式: 列	组 A Con	组 B DOX
1	0.946545799	1.450255968
2	0.961567047	1.886270339
3	1.091887155	1.667956682

描述性统计	A Con	B DOX
值的数目	3	3
最小	0.9465	1.450
最大值	1.092	1.886
范围	0.1453	0.4360
平均: mean	1.000	1.668
标准偏差 SD	0.07993	0.2180
标准误差的平均值	0.04615	0.1259

Bcl-2

格式: 列	组 A Con	组 B DOX
1	0.957353452	0.690052429
2	0.956281894	0.638543771
3	1.086364654	0.497948231

描述性统计	A Con	B DOX
值的数目	3	3
最小	0.9563	0.4979
最大值	1.086	0.6901
范围	0.1301	0.1921
平均: mean	1.000	0.6088
标准偏差 SD	0.07480	0.09944
标准误差的平均值	0.04318	0.05741

Cleaved Caspase3/Caspase3

格式: 列	组 A Con	组 B DOX
1	1.489647148	8.296204145
2	0.889779701	9.521978745
3	0.620573151	8.488335729

描述性统计	A Con	B DOX
值的数目	3	3
最小	0.6206	8.296
最大值	1.490	9.522
范围	0.8691	1.226
平均: mean	1.000	8.769
标准偏差 SD	0.4449	0.6593
标准误差的平均值	0.2569	0.3806

B

组 A Con	组 B DOX
0.202195369	8.334759118
0.246858957	7.825035902
0.262196058	8.154836863
0.241702087	7.890690844

描述性统计	A Con	B DOX
值的数目	4	4
最小	0.2022	7.825
最大值	0.2622	8.335
范围	0.06000	0.5097
平均: mean	0.2382	8.051
标准偏差 SD	0.02556	0.2367
标准误差的平均值	0.01278	0.1183

C

组 A Con	组 B DOX
184.9890732	59.33256962
173.0679532	51.44954740
195.1513394	49.97245250
180.6733672	56.59605697

描述性统计	A Con	B DOX
值的数目	4	4
最小	173.1	49.97
最大值	195.2	59.33
范围	22.08	9.360
平均: mean	183.5	54.34
标准偏差 SD	9.216	4.376
标准误差的平均值	4.608	2.188

E

p-AMPK/AMPK

组 A Con	组 B DOX
1.011378050	0.493984011
0.976472751	0.462155356
1.012149199	0.623194641

描述性统计	A Con	B DOX
值的数目	3	3
最小	0.9765	0.4622
最大值	1.012	0.6232
范围	0.03568	0.1610
平均: mean	1.000	0.5264
标准偏差 SD	0.02038	0.08529
标准误差的平均值	0.01177	0.04924

UCP2

组 A Con	组 B DOX
1.059882154	0.428420877
1.025840100	0.612741219
0.914277746	0.515509362

描述性统计	A Con	B DOX
值的数目	3	3
最小	0.9143	0.4284
最大值	1.060	0.6127
范围	0.1456	0.1843
平均: mean	1.000	0.5189
标准偏差 SD	0.07616	0.09221
标准误差的平均值	0.04397	0.05324

Fig 3

A

组 A	组 B	组 C	组 D	组 E	组 F
Con	30	50	100	300	500
101.665585500	103.491367900	99.549336660	98.055486350	[illegible]2.941483050	[illegible]0.607175890
94.342609100	95.051064110	90.724696080	91.435859880	[illegible]5.075705060	[illegible]1.550307920
105.522393800	106.193759100	103.750214400	103.713020500	[illegible]5.535418130	[illegible]0.064322240
98.469411610	99.522352860	98.443625930	94.917447410	[illegible]9.892938640	[illegible]6.298102500

描述性统计	A	B	C	D	E	F
	Con	30	50	100	300	500
值的数目	4	4	4	4	4	4
最小	94.34	95.05	90.72	91.44	85.08	81.55
最大值	105.5	106.2	103.8	103.7	95.54	90.61
范围	11.18	11.14	13.03	12.28	10.46	9.057
平均: mean	100.0	101.1	98.12	97.03	90.86	87.13
标准偏差 SD	4.748	4.856	5.433	5.211	4.494	4.184
标准误差的平均值	2.374	2.428	2.716	2.606	2.247	2.092

B

组 A	组 B	组 C	组 D	组 E	组 F	组 G
Con	0	30	50	100	300	500
95.075040640	55.787361130	[illegible]2.077956690	[illegible]5.195333340	74.932764580	[illegible]5.824154610	59.919378370
103.198433400	57.999398840	[illegible]5.833012700	69.119470210	76.082089690	59.653376460	53.751501120
98.912565020	49.064998100	57.466151770	58.507107650	[illegible]9.488039560	56.987348330	52.037174480
102.813960900	52.346430840	58.688620930	[illegible]3.152687300	71.218321130	59.897777050	58.718924950

描述性统计	A	B	C	D	E	F	G
	Con	0	30	50	100	300	500
值的数目	4	4	4	4	4	4	4
最小	95.08	49.06	57.47	58.51	69.49	56.99	52.04
最大值	103.2	58.00	65.83	69.12	76.08	65.82	59.92
范围	8.123	8.934	8.367	10.61	6.594	8.837	7.882
平均: mean	100.0	53.80	61.02	63.99	72.93	60.59	56.11
标准偏差 SD	3.812	3.921	3.757	4.417	3.094	3.730	3.806
标准误差的平均值	1.906	1.960	1.879	2.208	1.547	1.865	1.903

C

组 A	组 B	组 C	组 D
Con	DOX	AP39	DOX+AP39
102.360293900	51.979388560	102.611206300	74.885655280
101.775915000	52.412364150	104.882093600	73.921115890
96.722998270	50.395639370	100.007773300	72.302806650
99.140792900	50.613996010	107.156122800	72.945471780

描述性统计	A	B	C	D
	Con	DOX	AP39	DOX+AP39
值的数目	4	4	4	4
最小	96.72	50.40	100.0	72.30
最大值	102.4	52.41	107.2	74.89
范围	5.637	2.017	7.148	2.583
平均: mean	100.0	51.35	103.7	73.51
标准偏差 SD	2.595	0.9962	3.063	1.131
标准误差的平均值	1.297	0.4981	1.532	0.5655

D

组 A	组 B	组 C	组 D
Con	DOX	AP39	DOX+AP39
[illegible].095506706	[illegible].074498288	0.110346538	[illegible].085914794
0.096571181	[illegible].074772719	0.110001094	[illegible].081192344
[illegible].097345788	[illegible].073172088	0.111626188	[illegible].085875013
[illegible].096186431	[illegible].076132406	0.112589725	[illegible].088493569

描述性统计	A	B	C	D
	Con	DOX	AP39	DOX+AP39
值的数目	4	4	4	4
最小	0.09551	0.07317	0.1100	0.08119
最大值	0.09735	0.07613	0.1126	0.08849
范围	0.001839	0.002960	0.002589	0.007301
平均: mean	0.09640	0.07464	0.1111	0.08537
标准偏差 SD	0.0007675	0.001214	0.001192	0.003042
标准误差的平均值	0.0003838	0.0006069	0.0005962	0.001521

E

组 A	组 B	组 C	组 D
Con	DOX	AP39	DOX+AP39
1.163681973	2.620592642	0.962911992	2.042976099
1.115819455	2.668890670	[illegible].958034283	2.150808306
1.061816250	2.553567696	[illegible].934386105	1.929308062
1.015695770	2.674682949	[illegible].991873389	2.098503589
[illegible].642986551	2.666538917	[illegible].903813323	1.902175807

描述性统计	A	B	C	D
	Con	DOX	AP39	DOX+AP39
值的数目	5	5	5	5
最小	0.6430	2.554	0.9038	1.902
最大值	1.164	2.675	0.9919	2.151
范围	0.5207	0.1211	0.08806	0.2486
平均: mean	1.000	2.637	0.9502	2.025
标准偏差 SD	0.2072	0.05133	0.03302	0.1070
标准误差的平均值	0.09266	0.02296	0.01477	0.04785

F

SOD

组 A	组 B	组 C	组 D
Con	DOX	AP39	DOX+AP39
[illegible].008662683	[illegible].004039753	[illegible].008770934	[illegible].005782701
[illegible].008434927	[illegible].003726106	[illegible].009064018	[illegible].005843556
[illegible].008747237	[illegible].003969615	[illegible].008797399	[illegible].005937367
[illegible].008344061	[illegible].004364445	[illegible].008921801	[illegible].006052590

描述性统计	A	B	C	D
	Con	DOX	AP39	DOX+AP39
值的数目	4	4	4	4
最小	0.008344	0.003726	0.008771	0.005783
最大值	0.008747	0.004364	0.009064	0.006053
范围	0.0004032	0.0006383	0.0002931	0.0002699
平均: mean	0.008547	0.004025	0.008889	0.005904
标准偏差 SD	0.0001891	0.0002632	0.0001342	0.0001177
标准误差的平均值	9.453e-005	0.0001316	6.710e-005	5.885e-005

GSH-Px

组 A	组 B	组 C	组 D
Con	DOX	AP39	DOX+AP39
0.314506250	0.128025303	0.368224811	0.246649433
0.350539217	0.127896354	0.377489736	0.236411301
0.365825857	0.133115173	0.368195614	0.227367781
0.380470180	0.144109972	0.376883915	0.230365257

描述性统计	A	B	C	D
	Con	DOX	AP39	DOX+AP39
值的数目	4	4	4	4
最小	0.3145	0.1279	0.3682	0.2274
最大值	0.3805	0.1441	0.3775	0.2466
范围	0.06596	0.01621	0.009294	0.01928
平均: mean	0.3528	0.1333	0.3727	0.2352
标准偏差 SD	0.02832	0.007614	0.005189	0.008510
标准误差的平均值	0.01416	0.003807	0.002594	0.004255

MDA

组 A	组 B	组 C	组 D
Con	DOX	AP39	DOX+AP39
0.004187993	0.006960833	0.003925050	0.006247346
0.004596085	0.008002611	0.004610633	0.005750039
0.003925966	0.007038903	0.004459123	0.006037115
0.004360329	0.007520486	0.004342249	0.006603038

描述性统计	A	B	C	D
	Con	DOX	AP39	DOX+AP39
值的数目	4	4	4	4
最小	0.003926	0.006961	0.003925	0.005750
最大值	0.004596	0.008003	0.004611	0.006603
范围	0.0006701	0.001042	0.0006856	0.0008530
平均: mean	0.004268	0.007381	0.004334	0.006159
标准偏差 SD	0.0002826	0.0004828	0.0002941	0.0003592
标准误差的平均值	0.0001413	0.0002414	0.0001471	0.0001796

NADPH

组 A	组 B	组 C	组 D
Con	DOX	AP39	DOX+AP39
0.000435311	0.002320110	0.000491994	0.001751581
0.000409971	0.002444310	0.000470046	0.001743856
0.000480455	0.002446665	0.000417413	0.001846178
0.000471506	0.002511567	0.000407499	0.001863040

描述性统计	A	B	C	D
	Con	DOX	AP39	DOX+AP39
值的数目	4	4	4	4
最小	0.0004100	0.002320	0.0004075	0.001744
最大值	0.0004805	0.002512	0.0004920	0.001863
范围	7.048e-005	0.0001915	8.450e-005	0.0001192
平均: mean	0.0004493	0.002431	0.0004467	0.001801
标准偏差 SD	3.269e-005	8.002e-005	4.079e-005	6.218e-005
标准误差的平均值	1.635e-005	4.001e-005	2.039e-005	3.109e-005

G

组 A	组 B	组 C	组 D
Con	DOX	AP39	DOX+AP39
4.16	27.35	4.21	18.77
5.26	32.47	5.28	18.97
4.60	34.02	5.41	21.67

描述性统计	A	B	C	D
	Con	DOX	AP39	DOX+AP39
值的数目	3	3	3	3
最小	4.160	27.35	4.210	18.77
最大值	5.260	34.02	5.410	21.67
范围	1.100	6.670	1.200	2.900
平均: mean	4.673	31.28	4.967	19.80
标准偏差 SD	0.5537	3.491	0.6585	1.620
标准误差的平均值	0.3197	2.015	0.3802	0.9351

H

组 A	组 B	组 C	组 D
Con	DOX	AP39	DOX+AP39
8	29	3	20
6	26	7	18
5	30	8	15

描述性统计	A	B	C	D
	Con	DOX	AP39	DOX+AP39
值的数目	3	3	3	3
最小	5.000	26.00	3.000	15.00
最大值	8.000	30.00	8.000	20.00
范围	3.000	4.000	5.000	5.000
平均: mean	6.333	28.33	6.000	17.67
标准偏差 SD	1.528	2.082	2.646	2.517
标准误差的平均值	0.8819	1.202	1.528	1.453

Fig 4

Bax

组 A	组 B	组 C	组 D
Con	DOX	AP39	DOX+AP39
0.998934345	1.430962643	1.087204428	1.266941735
0.898009767	1.552209129	0.940565648	1.107945076
1.014655710	1.601159589	1.043117903	1.143760160
1.088400178	1.628349928	0.974901411	1.189940389

描述性统计	A	B	C	D
	Con	DOX	AP39	DOX+AP39
值的数目	4	4	4	4
最小	0.8980	1.431	0.9406	1.108
最大值	1.088	1.628	1.087	1.267
范围	0.1904	0.1974	0.1466	0.1590
平均: mean	1.000	1.553	1.011	1.177
标准偏差 SD	0.07838	0.08735	0.06609	0.06863
标准误差的平均值	0.03919	0.04368	0.03304	0.03432

Bcl-2

组 A	组 B	组 C	组 D
Con	DOX	AP39	DOX+AP39
1.075392362	0.431691066	1.033282780	0.745468940
1.018960414	0.582901429	0.976361139	0.839983301
0.905647224	0.659036235	0.962775383	0.723265620

描述性统计	A	B	C	D
	Con	DOX	AP39	DOX+AP39
值的数目	3	3	3	3
最小	0.9056	0.4317	0.9628	0.7233
最大值	1.075	0.6590	1.033	0.8400
范围	0.1697	0.2273	0.07051	0.1167
平均: mean	1.000	0.5579	0.9908	0.7696
标准偏差 SD	0.08645	0.1157	0.03741	0.06198
标准误差的平均值	0.04991	0.06681	0.02160	0.03578

Cleaved Caspase3/Caspase3

组 A	组 B	组 C	组 D
Con	DOX	AP39	DOX+AP39
1.417580065	7.451957476	1.522590091	3.685303216
0.880855447	7.135381649	0.669622225	4.427313878
1.067119093	7.697713503	1.403710746	5.193526113
0.634445395	7.599233420	0.370853436	3.154284733

描述性统计	A	B	C	D
	Con	DOX	AP39	DOX+AP39
值的数目	4	4	4	4
最小	0.6344	7.135	0.3709	3.154
最大值	1.418	7.698	1.523	5.194
范围	0.7831	0.5623	1.152	2.039
平均: mean	1.000	7.471	0.9917	4.115
标准偏差 SD	0.3300	0.2455	0.5600	0.8885
标准误差的平均值	0.1650	0.1228	0.2800	0.4443

B

组 A	组 B	组 C	组 D
Con	DOX	AP39	DOX+AP39
0.191405059	7.863583001	0.147899971	3.389454340
0.193911106	7.716663156	0.128900214	3.083059211
0.215355805	7.732113145	0.132252320	2.966009989
0.182854719	8.164694280	0.162479407	2.851950061

描述性统计	A	B	C	D
	Con	DOX	AP39	DOX+AP39
值的数目	4	4	4	4
最小	0.1829	7.717	0.1289	2.852
最大值	0.2154	8.165	0.1625	3.389
范围	0.03250	0.4480	0.03358	0.5375
平均: mean	0.1959	7.869	0.1429	3.073
标准偏差 SD	0.01382	0.2077	0.01547	0.2313
标准误差的平均值	0.006909	0.1038	0.007734	0.1157

C

组 A	组 B	组 C	组 D
Con	DOX	AP39	DOX+AP39
176.3739469	56.54941187	165.5965266	92.62950860
194.0927700	51.44954740	176.3092403	104.84477020
165.1205399	58.41521595	153.9293469	99.97775191
189.8422067	49.97245250	170.9412029	97.40977078

描述性统计	A	B	C	D
	Con	DOX	AP39	DOX+AP39
值的数目	4	4	4	4
最小	165.1	49.97	153.9	92.63
最大值	194.1	58.42	176.3	104.8
范围	28.97	8.443	22.38	12.22
平均: mean	181.4	54.10	166.7	98.72
标准偏差 SD	13.20	4.028	9.568	5.096
标准误差的平均值	6.600	2.014	4.784	2.548

E

p-AMPK/AMPK

组 A	组 B	组 C	组 D
Con	DOX	AP39	DOX+AP39
1.092081041	0.334754020	0.838035357	0.445305054
0.953486373	0.259550019	0.791336761	0.564518382
0.954432585	0.256161698	0.882477412	0.582489985

描述性统计	A	B	C	D
	Con	DOX	AP39	DOX+AP39
值的数目	3	3	3	3
最小	0.9535	0.2562	0.7913	0.4453
最大值	1.092	0.3348	0.8825	0.5825
范围	0.1386	0.07859	0.09114	0.1372
平均: mean	1.000	0.2835	0.8373	0.5308
标准偏差 SD	0.07975	0.04443	0.04557	0.07456
标准误差的平均值	0.04604	0.02565	0.02631	0.04305

UCP2

组 A	组 B	组 C	组 D
Con	DOX	AP39	DOX+AP39
0.941573659	0.559152881	0.953887231	0.743254192
0.950116790	0.560781617	1.100970179	0.952714602
1.117190059	0.704942782	0.968243341	0.776004365
0.991119492	0.663468423	0.954673888	0.840861334

描述性统计	A	B	C	D
	Con	DOX	AP39	DOX+AP39
值的数目	4	4	4	4
最小	0.9416	0.5592	0.9539	0.7433
最大值	1.117	0.7049	1.101	0.9527
范围	0.1756	0.1458	0.1471	0.2095
平均: mean	1.000	0.6221	0.9944	0.8282
标准偏差 SD	0.08106	0.07370	0.07132	0.09238
标准误差的平均值	0.04053	0.03685	0.03566	0.04619

Fig 5

A

AMPK

组 A	组 B
Con	CC
0.977696788	0.684741592
0.985608687	0.753948770
1.036694526	0.680053137

描述性统计	A	B
	Con	CC
值的数目	3	3
最小	0.9777	0.6801
最大值	1.037	0.7539
范围	0.05900	0.07390
平均: mean	1.000	0.7062
标准偏差 SD	0.03202	0.04138
标准误差的平均值	0.01849	0.02389

p-AMPK/AMPK

组 A	组 B
Con	CC
1.063703007	0.409524977
1.027935355	0.321809239
0.908361638	0.311172409

描述性统计	A	B
	Con	CC
值的数目	3	3
最小	0.9084	0.3112
最大值	1.064	0.4095
范围	0.1553	0.09835
平均: mean	1.000	0.3475
标准偏差 SD	0.08135	0.05398
标准误差的平均值	0.04697	0.03116

B

组 A	组 B	组 C	组 D	组 E	组 F
Con	DOX	DOX+AP39	CC	DOX+CC	DOX+AP39+CC
106.0977452	53.90865971	79.00711121	99.81176921	45.31292004	43.37396969
96.84739398	48.57633311	73.05043919	90.32312553	39.02646448	37.99741619
103.03516490	50.55919066	77.61353727	97.64097394	43.59259983	45.07617286
94.01969589	48.17466752	73.75007693	86.76951999	39.80352595	39.89677546

描述性统计	A	B	C	D	E	F
	Con	DOX	DOX+AP39	CC	DOX+CC	DOX+AP39+CC
值的数目	4	4	4	4	4	4
最小	94.02	48.17	73.05	86.77	39.03	38.00
最大值	106.1	53.91	79.01	99.81	45.31	45.08
范围	12.08	5.734	5.957	13.04	6.286	7.079
平均: mean	100.0	50.30	75.86	93.64	41.93	41.59
标准偏差 SD	5.541	2.619	2.905	6.118	3.009	3.220
标准误差的平均值	2.770	1.310	1.453	3.059	1.504	1.610

C

组 A	组 B	组 C	组 D	组 E	组 F
Con	DOX	DOX+AP39	CC	DOX+CC	DOX+AP39+CC
0.849115291	2.403452591	1.725199528	0.937833577	2.429502541	1.967566574
1.005697764	2.256554903	1.473147697	1.286697912	2.337532430	2.407305297
0.805922564	2.152390445	1.679391202	1.079994910	2.574314819	2.173880771
1.064159933	2.226334132	1.832686503	1.110463102	2.310952290	2.509985225
1.275104447	1.953392856	1.615697835	0.879512792	2.070812037	2.195265059

描述性统计	A	B	C	D	E	F
	Con	DOX	DOX+AP39	CC	DOX+CC	DOX+AP39+CC
值的数目	5	5	5	5	5	5
最小	0.8059	1.953	1.473	0.8795	2.071	1.968
最大值	1.275	2.403	1.833	1.287	2.574	2.510
范围	0.4692	0.4501	0.3595	0.4072	0.5035	0.5424
平均: mean	1.000	2.198	1.665	1.059	2.345	2.251
标准偏差 SD	0.1873	0.1646	0.1334	0.1596	0.1845	0.2127
标准误差的平均值	0.08374	0.07361	0.05966	0.07136	0.08250	0.09510

D

SOD

组 A	组 B	组 C	组 D	组 E	组 F
Con	DOX	DOX+AP39	CC	DOX+CC	DOX+AP39+CC
0.008372033	0.003743098	0.006496933	0.008531942	0.003398549	0.003130384
0.009346132	0.003674752	0.005963451	0.008650070	0.002815713	0.003202334
0.009350484	0.003686429	0.005853407	0.008733845	0.003047570	0.003187665
0.009000907	0.004067469	0.005879233	0.008631493	0.003466485	0.003142135

描述性统计	A	B	C	D	E	F
	Con	DOX	DOX+AP39	CC	DOX+CC	DOX+AP39+CC
值的数目	4	4	4	4	4	4
最小	0.008372	0.003675	0.005853	0.008532	0.002816	0.003130
最大值	0.009350	0.004067	0.006497	0.008734	0.003466	0.003202
范围	0.0009785	0.0003927	0.0006435	0.0002019	0.0006508	7.195e-005
平均: mean	0.009017	0.003793	0.006048	0.008637	0.003182	0.003166
标准偏差 SD	0.0004604	0.0001854	0.0003028	8.290e-005	0.0003055	3.477e-005
标准误差的平均值	0.0002302	9.272e-005	0.0001514	4.145e-005	0.0001528	1.739e-005

GSH-Px

组 A	组 B	组 C	组 D	组 E	组 F
Con	DOX	DOX+AP39	CC	DOX+CC	DOX+AP39+CC
0.312270308	0.133852377	0.242941516	0.340271890	0.099936133	0.109473313
0.369485113	0.131446124	0.222718287	0.343230437	0.116513001	0.090365378
0.377039628	0.148584289	0.233953955	0.316126638	0.111998297	0.110094948
0.375229464	0.159781029	0.234902831	0.330011861	0.104355707	0.107351480

描述性统计	A	B	C	D	E	F
	Con	DOX	DOX+AP39	CC	DOX+CC	DOX+AP39+CC
值的数目	4	4	4	4	4	4
最小	0.3123	0.1314	0.2227	0.3161	0.09994	0.09037
最大值	0.3770	0.1598	0.2429	0.3432	0.1165	0.1101
范围	0.06477	0.02833	0.02022	0.02710	0.01658	0.01973
平均: mean	0.3585	0.1434	0.2336	0.3324	0.1082	0.1043
标准偏差 SD	0.03099	0.01328	0.008317	0.01224	0.007452	0.009378
标准误差的平均值	0.01550	0.006641	0.004158	0.006122	0.003726	0.004689

MDA

组 A	组 B	组 C	组 D	组 E	组 F
Con	DOX	DOX+AP39	CC	DOX+CC	DOX+AP39+CC
0.003996857	0.007587985	0.006329899	0.004393274	0.007574608	0.007614365
0.004263080	0.007410385	0.005142983	0.004534697	0.008417132	0.007541687
0.004504240	0.006995374	0.005853722	0.004252870	0.007712328	0.007392021
0.003811871	0.006828033	0.006099312	0.004882416	0.007382604	0.008301755

描述性统计	A	B	C	D	E	F
	Con	DOX	DOX+AP39	CC	DOX+CC	DOX+AP39+CC
值的数目	4	4	4	4	4	4
最小	0.003812	0.006828	0.005143	0.004253	0.007383	0.007392
最大值	0.004504	0.007588	0.006330	0.004882	0.008417	0.008302
范围	0.0006924	0.0007600	0.001187	0.0006295	0.001035	0.0009097
平均: mean	0.004144	0.007205	0.005856	0.004516	0.007772	0.007712
标准偏差 SD	0.0003033	0.0003535	0.0005139	0.0002701	0.0004511	0.0004036
标准误差的平均值	0.0001516	0.0001768	0.0002569	0.0001351	0.0002255	0.0002018

NADPH

组 A	组 B	组 C	组 D	组 E	组 F
Con	DOX	DOX+AP39	CC	DOX+CC	DOX+AP39+CC
0.000429612	0.002962820	0.001738746	0.000490039	0.002639605	0.002654794
0.000404838	0.002587090	0.001741525	0.000534524	0.002759047	0.002937128
0.000433356	0.002532337	0.001734366	0.000550137	0.002919725	0.003225325
0.000437077	0.002621260	0.001708367	0.000523856	0.002953942	0.002926978

描述性统计	A	B	C	D	E	F
	Con	DOX	DOX+AP39	CC	DOX+CC	DOX+AP39+CC
值的数目	4	4	4	4	4	4
最小	0.0004048	0.002532	0.001708	0.0004900	0.002640	0.002655
最大值	0.0004371	0.002963	0.001742	0.0005501	0.002954	0.003225
范围	3.224e-005	0.0004305	3.316e-005	6.010e-005	0.0003143	0.0005705
平均: mean	0.0004262	0.002676	0.001731	0.0005246	0.002818	0.002936
标准偏差 SD	1.458e-005	0.0001948	1.521e-005	2.547e-005	0.0001462	0.0002330
标准误差的平均	7.289e-006	9.739e-005	7.605e-006	1.273e-005	7.310e-005	0.0001165

E

组 A	组 B	组 C	组 D	组 E	组 F
Con	DOX	DOX+AP39	CC	DOX+CC	DOX+AP39+CC
4.3	27.69	20.03	5.40	40.85	36.68
4.67	33.95	20.71	5.50	45.53	45.10
4.94	35.60	23.63	5.21	48.45	35.66

描述性统计	A	B	C	D	E	F
	Con	DOX	DOX+AP39	CC	DOX+CC	DOX+AP39+CC
值的数目	3	3	3	3	3	3
最小	4.300	27.69	20.03	5.210	40.85	35.66
最大值	4.940	35.60	23.63	5.500	48.45	45.10
范围	0.6400	7.910	3.600	0.2900	7.600	9.440
平均: mean	4.637	32.41	21.46	5.370	44.94	39.15
标准偏差 SD	0.3213	4.173	1.913	0.1473	3.834	5.181
标准误差的平均	0.1855	2.409	1.104	0.08505	2.213	2.991

F

组 A	组 B	组 C	组 D	组 E	组 F
Con	DOX	DOX+AP39	CC	DOX+CC	DOX+AP39+CC
4	25	18	4	28	30
4	29	18	5	27	28
3	27	16	6	31	29

描述性统计	A	B	C	D	E	F
	Con	DOX	DOX+AP39	CC	DOX+CC	DOX+AP39+CC
值的数目	3	3	3	3	3	3
最小	3.000	25.00	16.00	4.000	27.00	28.00
最大值	4.000	29.00	18.00	6.000	31.00	30.00
范围	1.000	4.000	2.000	2.000	4.000	2.000
平均: mean	3.667	27.00	17.33	5.000	28.67	29.00
标准偏差 SD	0.5774	2.000	1.155	1.000	2.082	1.000
标准误差的平均	0.3333	1.155	0.6667	0.5774	1.202	0.5774

G

Bax

组 A	组 B	组 C	组 D	组 E	组 F
Con	DOX	DOX+AP39	CC	DOX+CC	DOX+AP39+CC
1.062085793	1.566943957	1.101793170	1.033025107	1.550419151	1.392342192
1.011500301	1.455224980	1.258150325	1.040769760	1.622598749	1.530982263
0.926413906	1.596537471	1.335079013	1.260407789	1.862647968	1.641168993

描述性统计	A	B	C	D	E	F
	Con	DOX	DOX+AP39	CC	DOX+CC	DOX+AP39+CC
值的数目	3	3	3	3	3	3
最小	0.9264	1.455	1.102	1.033	1.550	1.392
最大值	1.062	1.597	1.335	1.260	1.863	1.641
范围	0.1357	0.1413	0.2333	0.2274	0.3122	0.2488
平均: mean	1.000	1.540	1.232	1.111	1.679	1.521
标准偏差 SD	0.06856	0.07453	0.1189	0.1291	0.1635	0.1247
标准误差的平均	0.03958	0.04303	0.06863	0.07454	0.09438	0.07199

Bcl-2

组 A	组 B	组 C	组 D	组 E	组 F
Con	DOX	DOX+AP39	CC	DOX+CC	DOX+AP39+CC
1.004724677	0.738160541	0.959878623	0.913002846	0.219669742	0.579482219
0.845367298	0.559872228	0.979282454	1.166424434	0.336451136	0.395747582
1.112429299	0.707091706	0.731967111	0.957021593	0.269481547	0.558348561
1.037478726	0.621466173	0.864459816	1.014286573	0.428607757	0.744580602

描述性统计	A	B	C	D	E	F
	Con	DOX	DOX+AP39	CC	DOX+CC	DOX+AP39+CC
值的数目	4	4	4	4	4	4
最小	0.8454	0.5599	0.7320	0.9130	0.2197	0.3957
最大值	1.112	0.7382	0.9793	1.166	0.4286	0.7446
范围	0.2671	0.1783	0.2473	0.2534	0.2089	0.3488
平均: mean	1.000	0.6566	0.8839	1.013	0.3136	0.5695
标准偏差 SD	0.1125	0.08122	0.1130	0.1106	0.09040	0.1427
标准误差的平均	0.05626	0.04061	0.05652	0.05528	0.04520	0.07134

Cleaved Caspase3/Caspase3

组 A	组 B	组 C	组 D	组 E	组 F
Con	DOX	DOX+AP39	CC	DOX+CC	DOX+AP39+CC
1.673860988	5.536055636	3.880118371	1.616956860	6.858311783	5.131797924
0.654018683	5.085797191	3.631031237	0.662470634	7.651676325	5.082344220
0.672120329	5.753174633	3.789945427	0.705268964	6.493622293	4.767015783

描述性统计	A	B	C	D	E	F
	Con	DOX	DOX+AP39	CC	DOX+CC	DOX+AP39+CC
值的数目	3	3	3	3	3	3
最小	0.6540	5.086	3.631	0.6625	6.494	4.767
最大值	1.674	5.753	3.880	1.617	7.652	5.132
范围	1.020	0.6674	0.2491	0.9545	1.158	0.3648
平均: mean	1.000	5.458	3.767	0.9949	7.001	4.994
标准偏差 SD	0.5837	0.3404	0.1261	0.5391	0.5921	0.1979
标准误差的平均	0.3370	0.1965	0.07281	0.3113	0.3419	0.1142

H

组 A	组 B	组 C	组 D	组 E	组 F
con	DOX	DOX+AP39	CC	DOX+CC	DOX+AP39+CC
0.142140612	7.279936871	2.584880519	0.143606592	11.699323240	8.727992324
0.120269891	7.564222326	2.470243674	0.116950758	7.409899175	8.213643178
0.111414700	7.414535120	2.150477622	0.149867060	10.069380870	5.813849959
0.123798238	7.404300617	2.639016427	0.140666777	9.100938967	6.985873212

描述性统计	A	B	C	D	E	F
	con	DOX	DOX+AP39	CC	DOX+CC	DOX+AP39+CC
值的数目	4	4	4	4	4	4
最小	0.1114	7.280	2.150	0.1170	7.410	5.814
最大值	0.1421	7.564	2.639	0.1499	11.70	8.728
范围	0.03073	0.2843	0.4885	0.03292	4.289	2.914
平均: mean	0.1244	7.416	2.461	0.1378	9.570	7.435
标准偏差 SD	0.01292	0.1164	0.2187	0.01440	1.795	1.305
标准误差的平均	0.006460	0.05818	0.1094	0.007201	0.8977	0.6524

I

组 A	组 B	组 C	组 D	组 E	组 F
Con	DOX	DOX+AP39	CC	DOX+CC	DOX+AP39+CC
175.3503826	54.29498912	93.43246753	174.4442184	52.00506289	51.58733583
175.6821715	55.98491399	94.34627465	170.9571747	50.37551744	51.88842923
176.9750037	56.79624200	96.34630532	174.2857164	49.24235660	54.56816049
179.1888359	54.71336858	97.31183737	171.2588397	46.17680555	49.79033395

描述性统计	A	B	C	D	E	F
	Con	DOX	DOX+AP39	CC	DOX+CC	DOX+AP39+CC
值的数目	4	4	4	4	4	4
最小	175.4	54.29	93.43	171.0	46.18	49.79
最大值	179.2	56.80	97.31	174.4	52.01	54.57
范围	3.838	2.501	3.879	3.487	5.828	4.778
平均: mean	176.8	55.45	95.36	172.7	49.45	51.96
标准偏差 SD	1.741	1.151	1.782	1.886	2.459	1.971
标准误差的平均值	0.8703	0.5756	0.8909	0.9428	1.230	0.9855

J

组 A	组 B	组 C	组 D	组 E	组 F
Con	DOX	DOX+AP39	CC	DOX+CC	DOX+AP39+CC
0.977195514	0.577961228	0.754631683	0.748911120	0.208517183	0.485621343
0.913412816	0.654810115	0.790503857	0.843334358	0.368515235	0.605658390
1.109391670	0.583757532	0.852648189	0.877586788	0.393826517	0.544962724

描述性统计	A	B	C	D	E	F
	Con	DOX	DOX+AP39	CC	DOX+CC	DOX+AP39+CC
值的数目	3	3	3	3	3	3
最小	0.9134	0.5780	0.7546	0.7489	0.2085	0.4856
最大值	1.109	0.6548	0.8526	0.8776	0.3938	0.6057
范围	0.1960	0.07685	0.09802	0.1287	0.1853	0.1200
平均: mean	1.000	0.6055	0.7993	0.8233	0.3236	0.5454
标准偏差 SD	0.09996	0.04279	0.04959	0.06664	0.1005	0.06002
标准误差的平均值	0.05771	0.02471	0.02863	0.03848	0.05801	0.03465

Fig 6

A

组 A	组 B	组 C
Con	NC	siUCP2
0.950742278	0.879999948	0.368179408
0.999695843	0.921643904	0.311977624
1.049561879	1.039502657	0.313863980

描述性统计	A	B	C
	Con	NC	siUCP2
值的数目	3	3	3
最小	0.9507	0.8800	0.3120
最大值	1.050	1.040	0.3682
范围	0.09882	0.1595	0.05620
平均: mean	1.000	0.9470	0.3313
标准偏差 SD	0.04941	0.08273	0.03192
标准误差的平均值	0.02853	0.04776	0.01843

组 A	组 B	组 C
Con	NC	siUCP2
1	0.986232704	0.314253344
1	1.057018041	0.314253344
1	1.049716684	0.309926925

描述性统计	A	B	C
	Con	NC	siUCP2
值的数目	3	3	3
最小	1.000	0.9862	0.3099
最大值	1.000	1.057	0.3143
范围	0.000	0.07079	0.004326
平均: mean	1.000	1.031	0.3128
标准偏差 SD	0.000	0.03893	0.002498
标准误差的平均值	0.000	0.02248	0.001442

B

组 A	组 B	组 C	组 D	组 E	组 F
Con	DOX	DOX+AP39	DOX+AP39+siUCP2	NC	siUCP2
106.32378670	57.14878061	75.71489713	45.32754977	102.10102730	98.70886030
98.29999349	55.82492175	74.25765529	39.65855481	97.76193915	94.23023222
94.18784172	50.33426115	71.07477103	38.57236613	92.99664663	88.95441195
101.18837810	53.25152365	76.33718762	42.96713946	99.22631873	96.13513528

描述性统计	A	B	C	D	E	F
	Con	DOX	DOX+AP39	DOX+AP39+siUCP2	NC	siUCP2
值的数目	4	4	4	4	4	4
最小	94.19	50.33	71.07	38.57	93.00	88.95
最大值	106.3	57.15	76.34	45.33	102.1	98.71
范围	12.14	6.815	5.262	6.755	9.104	9.754
平均: mean	100.0	54.14	74.35	41.63	98.02	94.51
标准偏差 SD	5.101	3.009	2.349	3.093	3.804	4.132
标准误差的平均值	2.551	1.505	1.174	1.546	1.902	2.066

C

组 A	组 B	组 C	组 D	组 E	组 F
Con	DOX	DOX+AP39	DOX+AP39+siUCP2	NC	siUCP2
1.053103350	2.475266187	2.131254660	2.788365683	0.957728427	1.117859447
1.105879988	2.716991564	2.042665304	2.975638974	0.850625361	1.262031817
1.029395749	2.394132578	2.100216971	2.485905287	1.098382354	1.053689757
1.010044316	2.359576447	2.132930109	2.455621550	0.954628846	1.275309749
0.801576597	2.431034338	2.089829188	2.686079534	0.882626434	0.818121654

描述性统计	A	B	C	D	E	F
	Con	DOX	DOX+AP39	DOX+AP39+siUCP2	NC	siUCP2
值的数目	5	5	5	5	5	5
最小	0.8016	2.360	2.043	2.456	0.8506	0.8181
最大值	1.106	2.717	2.133	2.976	1.098	1.275
范围	0.3043	0.3574	0.09026	0.5200	0.2478	0.4572
平均: mean	1.000	2.475	2.099	2.678	0.9488	1.105
标准偏差 SD	0.1166	0.1417	0.03691	0.2163	0.09553	0.1863
标准误差的平均值	0.05214	0.06339	0.01651	0.09674	0.04272	0.08329

D

SOD

组 A	组 B	组 C	组 D	组 E	组 F
Con	DOX	DOX+AP39	DOX+AP39+siUCP2	NC	siUCP2
0.009182118	0.003919492	0.005859623	0.003209010	0.008941825	0.008806531
0.009071806	0.004200491	0.005800729	0.002847338	0.009040840	0.008804729
0.008899938	0.003639225	0.005843387	0.003231389	0.008874317	0.008796893
0.008919212	0.004177047	0.006019057	0.003451808	0.008972851	0.008429640

描述性统计	A	B	C	D	E	F
	Con	DOX	DOX+AP39	DOX+AP39+siUCP2	NC	siUCP2
值的数目	4	4	4	4	4	4
最小	0.008900	0.003639	0.005801	0.002847	0.008874	0.008430
最大值	0.009182	0.004200	0.006019	0.003452	0.009041	0.008807
范围	0.0002822	0.0005613	0.0002183	0.0006045	0.0001665	0.0003769
平均: mean	0.009018	0.003984	0.005881	0.003185	0.008957	0.008709
标准偏差 SD	0.0001336	0.0002628	9.552e-005	0.0002503	6.915e-005	0.0001866
标准误差的平均值	6.679e-005	0.0001314	4.776e-005	0.0001251	3.458e-005	9.329e-005

GSH-Px

组 A	组 B	组 C	组 D	组 E	组 F
Con	DOX	DOX+AP39	DOX+AP39+siUCP2	NC	siUCP2
0.304791217	0.124577720	0.238975700	0.097096804	0.356804233	0.340271890
0.365692041	0.136937441	0.224309480	0.108996685	0.360482954	0.343230437
0.373120039	0.132083574	0.233769046	0.114028162	0.363334448	0.325858703
0.365120282	0.156238557	0.248097077	0.109677930	0.375947204	0.330011861

描述性统计	A	B	C	D	E	F
	Con	DOX	DOX+AP39	DOX+AP39+siUCP2	NC	siUCP2
值的数目	4	4	4	4	4	4
最小	0.3048	0.1246	0.2243	0.09710	0.3568	0.3259
最大值	0.3731	0.1562	0.2481	0.1140	0.3759	0.3432
范围	0.06833	0.03166	0.02379	0.01693	0.01914	0.01737
平均: mean	0.3522	0.1375	0.2363	0.1074	0.3641	0.3348
标准偏差 SD	0.03180	0.01351	0.009942	0.007253	0.008312	0.008244
标准误差的平均值	0.01590	0.006756	0.004971	0.003626	0.004156	0.004122

MDA

组 A	组 B	组 C	组 D	组 E	组 F
Con	DOX	DOX+AP39	DOX+AP39+siUCP2	NC	siUCP2
0.004274026	0.007750595	0.006432313	0.007636587	0.004601467	0.004449500
0.004099678	0.007515079	0.006238529	0.007793675	0.004085243	0.004538954
0.004584150	0.006945217	0.005693452	0.007839690	0.004403981	0.004286702
0.003899013	0.007020243	0.006033445	0.008467133	0.003730343	0.004392526

描述性统计	A	B	C	D	E	F
	Con	DOX	DOX+AP39	DOX+AP39+siUCP2	NC	siUCP2
值的数目	4	4	4	4	4	4
最小	0.003899	0.006945	0.005693	0.007637	0.003730	0.004287
最大值	0.004584	0.007751	0.006432	0.008467	0.004601	0.004539
范围	0.0006851	0.0008054	0.0007389	0.0008305	0.0008711	0.0002523
平均: mean	0.004214	0.007308	0.006099	0.007934	0.004205	0.004417
标准偏差 SD	0.0002903	0.0003887	0.0003159	0.0003657	0.0003814	0.0001057
标准误差的平均值	0.0001452	0.0001943	0.0001579	0.0001829	0.0001907	5.284e-005

NADPH

组 A	组 B	组 C	组 D	组 E	组 F
Con	DOX	DOX+AP39	DOX+AP39+siUCP2	NC	siUCP2
0.0004252315	0.002654794	0.001843023	0.002594366	0.000435122	0.000482245
0.0004595194	0.002652651	0.001815800	0.002745813	0.000486695	0.000485400
0.0004343922	0.002729493	0.001742279	0.002740161	0.000484152	0.000541966
0.0004456252	0.002494917	0.001819968	0.002767148	0.000466561	0.000546228

描述性统计	A	B	C	D	E	F
	Con	DOX	DOX+AP39	DOX+AP39+siUCP2	NC	siUCP2
值的数目	4	4	4	4	4	4
最小	0.0004252	0.002495	0.001742	0.002594	0.0004351	0.0004822
最大值	0.0004595	0.002729	0.001843	0.002767	0.0004867	0.0005462
范围	3.429e-005	0.0002346	0.0001007	0.0001728	5.157e-005	6.398e-005
平均: mean	0.0004412	0.002633	0.001805	0.002712	0.0004681	0.0005140
标准偏差 SD	1.479e-005	9.872e-005	4.367e-005	7.919e-005	2.376e-005	3.487e-005
标准误差的平均值	7.397e-006	4.936e-005	2.183e-005	3.960e-005	1.188e-005	1.743e-005

E

组 A	组 B	组 C	组 D	组 E	组 F
Con	DOX	DOX+AP39	DOX+AP39+siUCP2	NC	siUCP2
4.56	33.03	20.31	33.07	4.57	4.64
4.87	32.52	20.68	33.54	4.79	4.67
5.10	28.92	20.83	33.95	5.09	5.18

描述性统计	A	B	C	D	E	F
	Con	DOX	DOX+AP39	DOX+AP39+siUCP2	NC	siUCP2
值的数目	3	3	3	3	3	3
最小	4.560	28.92	20.31	33.07	4.570	4.640
最大值	5.100	33.03	20.83	33.95	5.090	5.180
范围	0.5400	4.110	0.5200	0.8800	0.5200	0.5400
平均: mean	4.843	31.49	20.61	33.52	4.817	4.830
标准偏差 SD	0.2710	2.240	0.2676	0.4403	0.2610	0.3035
标准误差的平均值	0.1565	1.293	0.1545	0.2542	0.1507	0.1752

F

组 A	组 B	组 C	组 D	组 E	组 F
Con	DOX	DOX+AP39	DOX+AP39+siUCP2	NC	siUCP2
6	29	15	25	8	4
5	28	18	29	8	7
5	30	20	30	4	4

描述性统计	A	B	C	D	E	F
	Con	DOX	DOX+AP39	DOX+AP39+siUCP2	NC	siUCP2
值的数目	3	3	3	3	3	3
最小	5.000	28.00	15.00	25.00	4.000	4.000
最大值	6.000	30.00	20.00	30.00	8.000	7.000
范围	1.000	2.000	5.000	5.000	4.000	3.000
平均: mean	5.333	29.00	17.67	28.00	6.667	5.000
标准偏差 SD	0.5774	1.000	2.517	2.646	2.309	1.732
标准误差的平均值	0.3333	0.5774	1.453	1.528	1.333	1.000

G

Bax

组 A	组 B	组 C	组 D	组 E	组 F
Con	DOX	DOX+AP39	DOX+AP39+siUCP2	NC	siUCP2
1.142556626	1.742697100	1.346991602	1.571641602	0.963802528	1.039686938
0.998608937	1.375682172	1.224971256	1.665825742	1.158940145	1.325418393
1.041953438	1.761882209	1.451549388	1.904063452	1.354120343	1.390311822
0.816880999	1.668585640	1.363150822	1.837235679	0.868051009	1.054981969

描述性统计	A	B	C	D	E	F
	Con	DOX	DOX+AP39	DOX+AP39+siUCP2	NC	siUCP2
值的数目	4	4	4	4	4	4
最小	0.8169	1.376	1.225	1.572	0.8681	1.040
最大值	1.143	1.762	1.452	1.904	1.354	1.390
范围	0.3257	0.3862	0.2266	0.3324	0.4861	0.3506
平均: mean	1.000	1.637	1.347	1.745	1.086	1.203
标准偏差 SD	0.1362	0.1789	0.09324	0.1529	0.2157	0.1813
标准误差的平均值	0.06808	0.08947	0.04662	0.07645	0.1079	0.09067

Bcl-2

组 A	组 B	组 C	组 D	组 E	组 F
Con	DOX	DOX+AP39	DOX+AP39+siUCP2	NC	siUCP2
0.923749415	0.551540216	0.753774549	0.245759728	1.017979715	0.879956904
0.965688010	0.484229149	0.779466145	0.167541085	0.955079917	1.089986215
1.110562575	0.561698050	0.927867628	0.263273566	1.013248309	1.049928124

描述性统计	A	B	C	D	E	F
	Con	DOX	DOX+AP39	DOX+AP39+siUCP2	NC	siUCP2
值的数目	3	3	3	3	3	3
最小	0.9237	0.4842	0.7538	0.1675	0.9551	0.8800
最大值	1.111	0.5617	0.9279	0.2633	1.018	1.090
范围	0.1868	0.07747	0.1741	0.09573	0.06290	0.2100
平均: mean	1.000	0.5325	0.8204	0.2255	0.9954	1.007
标准偏差 SD	0.09802	0.04210	0.09398	0.05097	0.03503	0.1115
标准误差的平均值	0.05659	0.02431	0.05426	0.02943	0.02022	0.06438

Cleaved Caspase3/Caspase3

组 A	组 B	组 C	组 D	组 E	组 F
Con	DOX	DOX+AP39	DOX+AP39+siUCP2	NC	siUCP2
0.732526141	7.882589206	6.164710181	10.49670954	1.239472377	1.165335397
1.076754374	7.423757670	4.910581257	11.49711074	1.121588306	1.600547314
1.190719485	6.098588350	3.630494050	10.39680251	1.486499545	1.751834506

描述性统计	A	B	C	D	E	F
	Con	DOX	DOX+AP39	DOX+AP39+siUCP2	NC	siUCP2
值的数目	3	3	3	3	3	3
最小	0.7325	6.099	3.630	10.40	1.122	1.165
最大值	1.191	7.883	6.165	11.50	1.486	1.752
范围	0.4582	1.784	2.534	1.100	0.3649	0.5865
平均: mean	1.000	7.135	4.902	10.80	1.283	1.506
标准偏差 SD	0.2385	0.9264	1.267	0.6085	0.1862	0.3045
标准误差的平均值	0.1377	0.5349	0.7316	0.3513	0.1075	0.1758

H

组 A	组 B	组 C	组 D	组 E	组 F
Con	DOX	DOX+AP39	DOX+AP39+siUCP2	NC	siUCP2
0.163614496	7.851646172	2.709296882	10.045497630	0.133020736	0.143550131
0.145316361	7.117533719	2.772682490	9.152889076	0.085930123	0.110643119
0.157014157	6.869391635	2.586979167	10.462821730	0.181902849	0.119155824
0.160803791	7.445937018	2.523288217	8.242680657	0.163084792	0.140834594

描述性统计	A	B	C	D	E	F
	Con	DOX	DOX+AP39	DOX+AP39+siUCP2	NC	siUCP2
值的数目	4	4	4	4	4	4
最小	0.1453	6.869	2.523	8.243	0.08593	0.1106
最大值	0.1636	7.852	2.773	10.46	0.1819	0.1436
范围	0.01830	0.9823	0.2494	2.220	0.09597	0.03291
平均: mean	0.1567	7.321	2.648	9.476	0.1410	0.1285
标准偏差 SD	0.008049	0.4253	0.1134	0.9872	0.04186	0.01617
标准误差的平均值	0.004024	0.2126	0.05670	0.4936	0.02093	0.008087

I

组 A	组 B	组 C	组 D	组 E	组 F
Con	DOX	DOX+AP39	DOX+AP39+siUCP2	NC	siUCP2
179.1160654	53.11235818	97.63633158	48.93649879	174.0102090	173.77733040
182.0826157	57.48994588	95.19142253	51.14385417	177.4412945	170.36681470
176.2272106	60.88880463	100.39241090	52.36523034	179.9499597	184.01146870
175.3372455	56.64764581	102.78397650	47.82467587	182.0981804	174.76266830

描述性统计	A	B	C	D	E	F
	Con	DOX	DOX+AP39	DOX+AP39+siUCP2	NC	siUCP2
值的数目	4	4	4	4	4	4
最小	175.3	53.11	95.19	47.82	174.0	170.4
最大值	182.1	60.89	102.8	52.37	182.1	184.0
范围	6.745	7.776	7.593	4.541	8.088	13.64
平均: mean	178.2	57.03	99.00	50.07	178.4	175.7
标准偏差 SD	3.055	3.194	3.298	2.061	3.477	5.834
标准误差的平均值	1.528	1.597	1.649	1.031	1.738	2.917

J

组 A	组 B	组 C	组 D	组 E	组 F
Con	DOX	DOX+AP39	DOX+AP39+siUCP2	NC	siUCP2
0.770179746	0.211503904	0.469797834	0.364169292	0.786771523	0.885708867
1.081631080	0.180444043	0.441329971	0.393515138	1.028296017	1.023598721
1.148189175	0.319857334	0.515548457	0.614892551	1.030056732	0.969139273

描述性统计	A	B	C	D	E	F
	Con	DOX	DOX+AP39	DOX+AP39+siUCP2	NC	siUCP2
值的数目	3	3	3	3	3	3
最小	0.7702	0.1804	0.4413	0.3642	0.7868	0.8857
最大值	1.148	0.3199	0.5155	0.6149	1.030	1.024
范围	0.3780	0.1394	0.07422	0.2507	0.2433	0.1379
平均: mean	1.000	0.2373	0.4756	0.4575	0.9484	0.9595
标准偏差 SD	0.2018	0.07319	0.03744	0.1371	0.1400	0.06945
标准误差的平均值	0.1165	0.04226	0.02162	0.07914	0.08080	0.04010

Fig 7

A

组 A Con									
A:Y1	A:Y2	A:Y3	A:Y4	A:Y5	A:Y6	A:Y7	A:Y8	A:Y9	A:Y10
306.30	316.80	308.30	302.30	313.10	316.30	318.10	316.80	315.20	306.70
325.90	333.10	319.60	315.30	320.50	345.10	345.20	347.20	326.50	311.90
340.70	352.10	332.80	342.40	330.10	351.30	363.00	370.10	340.60	329.80
345.80	356.80	356.20	350.40	348.80	368.60	380.90	382.30	349.60	339.50
362.30	359.40	370.60	372.60	362.30	388.30	396.70	405.10	358.80	356.00
384.20	361.40	375.90	384.20	374.20	397.70	414.30	424.80	360.90	366.70
390.60	370.20	387.10	390.10	387.40	420.90	426.60	422.60	364.20	368.40
402.80	383.20	395.20	402.30	401.90	431.50	445.40	412.30	378.50	396.30

组 B DOX									
B:Y1	B:Y2	B:Y3	B:Y4	B:Y5	B:Y6	B:Y7	B:Y8	B:Y9	B:Y10
307.9	312.4	312.2	310.2	303.6	314.4	317.2	319.7	300.7	312.9
301.2	308.9	313.6	308.1	288.5	307.6	315.7	310.0	298.2	313.7
290.5	303.5	317.2	304.8	272.5	305.5	311.4	302.8	292.5	314.5
279.6	289.4	295.6	286.8	270.3	293.5	295.8	300.9	276.7	310.3
260.5	274.3	279.3	270.4	269.1	277.4	270.6	297.1	270.3	309.8
266.4	270.5	262.3	266.6	250.5	251.2	264.6	289.3	261.5	308.7
251.8	248.6	234.9	240.4	227.9	232.8	238.5	255.3	244.5	260.2
245.2	216.7	205.9	196.7	199.2	195.1	201.9	219.1	218.5	223.5

组 C AP39									
C:Y1	C:Y2	C:Y3	C:Y4	C:Y5	C:Y6	C:Y7	C:Y8	C:Y9	C:Y10
313.6	313.9	313.9	316.4	317.3	308.9	325.7	303.2	307.5	315.3
325.9	357.2	330.6	338.5	335.9	322.5	345.4	342.0	333.5	329.5
350.5	379.3	356.4	342.7	345.4	334.5	370.3	366.7	346.9	345.6
352.8	382.5	368.2	360.9	348.4	340.1	378.3	368.9	352.8	358.2
360.3	400.1	396.6	385.4	359.7	355.9	400.2	370.3	360.7	371.6
367.8	406.9	406.5	396.1	365.2	376.4	409.7	375.2	365.3	395.3
369.2	411.9	409.7	404.1	389.2	382.6	416.8	391.5	377.9	405.6
361.4	429.7	418.9	406.8	380.8	394.3	430.5	402.6	395.4	403.5

组 D DOX+AP39								
D:Y2	D:Y3	D:Y4	D:Y5	D:Y6	D:Y7	D:Y8	D:Y9	D:Y10
317.4	314.3	308.6	303.3	314.6	305.3	315.2	318.5	305.4
310.6	316.9	300.2	289.0	300.7	298.4	296.8	301.5	313.9
296.5	298.5	296.6	262.6	277.4	273.4	279.8	294.6	324.0
290.7	295.7	274.3	265.4	280.2	282.3	259.4	295.7	328.8
275.9	285.6	256.5	268.3	275.3	275.0	247.9	289.1	338.9
265.3	278.7	246.4	270.2	268.3	273.5	242.3	283.2	335.2
254.8	279.9	240.7	266.8	266.0	271.6	238.0	270.2	302.6
241.5	279.5	236.2	258.3	265.2	269.5	230.9	266.3	295.4

组 E DOX+AP39+CC									
E:Y1	E:Y2	E:Y3	E:Y4	E:Y5	E:Y6	E:Y7	E:Y8	E:Y9	E:Y10
300.2	313.7	302.4	302.2	317.6	311.9	310.3	300.7	305.8	309.5
295.2	307.0	292.5	301.6	284.3	303.8	295.7	294.9	302.6	300.5
285.3	290.2	288.5	292.4	270.5	298.1	272.3	290.7	285.6	294.8
270.4	278.2	280.9	277.3	258.3	292.0	270.9	283.2	277.5	274.3
263.5	270.5	263.7	259.1	240.9	283.5	263.3	275.6	260.6	270.2
260.3	265.2	258.9	240.5	246.8	277.2	257.4	269.3	252.9	268.5
238.3	244.0	227.6	228.5	239.3	241.3	251.5	237.5	229.7	246.9
225.3	236.5	207.6	219.5	228.4	219.6	231.5	229.7	205.3	231.4

组 F DOX+AP39+Genipin									
F:Y1	F:Y2	F:Y3	F:Y4	F:Y5	F:Y6	F:Y7	F:Y8	F:Y9	F:Y10
310.7	299.9	310.5	302.4	310.3	314.5	315.6	311.3	306.2	308.1
290.1	289.3	308.4	278.5	288.2	310.6	295.4	305.6	274.3	284.9
273.2	265.7	291.5	267.3	256.9	305.2	288.4	298.5	259.4	270.3
265.5	260.2	277.3	254.1	262.3	283.5	280.5	283.6	251.6	265.3
241.8	241.9	256.1	240.5	260.2	267.2	269.7	264.8	239.8	233.6
245.3	232.9	242.5	230.2	258.7	260.8	261.7	256.3	232.5	223.9
240.2	225.5	239.0	218.7	236.6	255.8	260.3	250.5	212.6	219.7
234.3	208.2	235.6	195.5	202.7	246.7	258.4	249.2	201.5	215.8

描述性统计	A	B	C	D	E	F
	Con	DOX	AP39	DOX+AP39	DOX+AP39+CC	DOX+AP39+Genipin
值的数目	8	8	8	8	8	8
最小	312.0	212.2	313.6	262.1	223.5	224.8
最大值	404.9	311.1	402.4	311.6	307.4	309.0
范围	92.95	98.94	88.82	49.49	83.95	84.16
平均: mean	362.4	276.5	365.7	285.2	269.4	263.0
标准偏差 SD	32.30	34.20	30.59	17.17	28.74	29.02
标准误差的平均値	11.42	12.09	10.81	6.069	10.16	10.26

B

组 A	组 B	组 C	组 D	组 E	组 F
Con	DOX	AP39	DOX+AP39	DOX+AP39+CC	DOX+AP39+Genipin
2.703574975	3.087275693	2.772551190	2.715517241	2.671992898	2.552283397
2.479123173	2.967235810	2.327205027	2.770186335	2.676532770	2.992315082
2.532894737	3.035454104	2.814514204	2.787119857	2.803468208	2.538200340
2.450907283	2.928317234	2.598328417	2.485182049	3.056947608	3.539641944
2.659865638	3.132530120	2.623424370	2.768099110	3.021015762	3.221509620
2.699884125	2.895950794	2.754248034	2.462292609	2.937158470	2.752330766
2.649303996	3.224368499	2.427409988	2.534322820	3.084233261	2.608359133
2.512733447	2.907348243	2.550919026	2.823733218	2.973443622	2.917335474
2.570673712	2.764302059	2.551846232	2.944048066	2.932294204	3.359801489
2.644461267	3.033557047	2.636926890	2.802979012	2.908383751	3.262279889

描述性统计	A	B	C	D	E	F
	Con	DOX	AP39	DOX+AP39	DOX+AP39+CC	DOX+AP39+Genipin
值的数目	10	10	10	10	10	10
最小	2.451	2.764	2.327	2.462	2.672	2.538
最大值	2.704	3.224	2.815	2.944	3.084	3.540
范围	0.2527	0.4601	0.4873	0.4818	0.4122	1.001
平均: mean	2.590	2.998	2.606	2.709	2.907	2.974
标准偏差 SD	0.09285	0.1329	0.1528	0.1606	0.1460	0.3601
标准误差的平均値	0.02936	0.04204	0.04832	0.05078	0.04618	0.1139

C

EF

组 A	组 B	组 C	组 D	组 E	组 F
Con	DOX	AP39	DOX+AP39	DOX+AP39+CC	DOX+AP39+Genipin
95.55	75.57	95.96	83.05	70.54	72.75
94.35	73.59	94.58	84.35	72.08	72.11
93.55	74.22	95.39	84.35	74.78	73.98
95.28	78.61	94.14	83.92	74.78	69.58
96.90	74.76	95.81	82.15	85.82	73.85
94.35	79.52	95.96	86.06	80.93	72.95
94.49	73.68	93.50	85.12	71.17	73.07

描述性统计	A	B	C	D	E	F
	Con	DOX	AP39	DOX+AP39	DOX+AP39+CC	DOX+AP39+Genipin
值的数目	7	7	7	7	7	7
最小	93.55	73.59	93.50	82.15	70.54	69.58
最大值	96.90	79.52	95.96	86.06	85.82	73.98
范围	3.350	5.930	2.460	3.910	15.28	4.400
平均: mean	94.92	75.71	95.05	84.14	75.73	72.61
标准偏差 SD	1.092	2.404	0.9833	1.287	5.653	1.483
标准误差的平均値	0.4128	0.9087	0.3716	0.4863	2.136	0.5604

FS

组 A	组 B	组 C	组 D	组 E	组 F
Con	DOX	AP39	DOX+AP39	DOX+AP39+CC	DOX+AP39+Genipin
66.34	39.08	67.57	46.60	35.24	36.84
63.55	37.62	63.92	48.08	36.36	36.26
61.95	37.93	66.06	48.11	38.54	37.78
65.71	41.67	63.25	47.57	38.46	34.38
70.43	38.37	67.00	45.63	49.32	37.86
63.55	42.47	67.57	50.00	44.44	36.96
63.89	37.50	61.62	49.11	35.90	37.39

描述性统计	A	B	C	D	E	F
	Con	DOX	AP39	DOX+AP39	DOX+AP39+CC	DOX+AP39+Genipin
值的数目	7	7	7	7	7	7
最小	61.95	37.50	61.62	45.63	35.24	34.38
最大值	70.43	42.47	67.57	50.00	49.32	37.86
范围	8.480	4.970	5.950	4.370	14.08	3.480
平均: mean	65.06	39.23	65.28	47.87	39.75	36.78
标准偏差 SD	2.784	2.020	2.360	1.466	5.218	1.198
标准误差的平均值	1.052	0.7636	0.8920	0.5541	1.972	0.4526

E/A

组 A	组 B	组 C	组 D	组 E	组 F
Con	DOX	AP39	DOX+AP39	DOX+AP39+CC	DOX+AP39+Genipin
1.76	0.71	1.31	1.42	0.58	0.63
1.41	0.56	2.14	1.93	0.66	1.27
1.52	0.61	1.51	1.30	1.47	0.71
1.88	0.38	1.63	1.52	1.43	0.43
1.43	0.58	1.55	1.74	0.67	0.43
2.12	0.57	1.63	1.38	0.55	1.33
1.41	0.61	1.45	1.56	0.64	0.54

描述性统计	A	B	C	D	E	F
	Con	DOX	AP39	DOX+AP39	DOX+AP39+CC	DOX+AP39+Genipin
值的数目	7	7	7	7	7	7
最小	1.410	0.3800	1.310	1.300	0.5500	0.4300
最大值	2.120	0.7100	2.140	1.930	1.470	1.330
范围	0.7100	0.3300	0.8300	0.6300	0.9200	0.9000
平均: mean	1.647	0.5743	1.603	1.550	0.8571	0.7629
标准偏差 SD	0.2788	0.09914	0.2616	0.2198	0.4074	0.3809
标准误差的平均值	0.1054	0.03747	0.09887	0.08307	0.1540	0.1440

D

CK-MB

组 A	组 B	组 C	组 D	组 E	组 F
Con	DOX	AP39	DOX+AP39	DOX+AP39+CC	DOX+AP39+Genipin
124.74319530	152.4686391	111.7088757	142.6295858	263.8635503	267.7810651
149.37633140	288.6946746	127.3349112	166.9976331	275.6771598	233.3899408
119.82248520	241.1786982	117.6272189	159.6449704	239.6021302	209.1466272
133.60710060	220.0863905	158.2402367	168.6248521	251.1885207	245.1615385
129.71242600	197.0390533	131.5609467	164.6272189	202.8727811	221.3852071
99.39585799	286.8828402	100.0757396	167.5692308	263.8301775	257.0105325
120.69301780	274.6201183	143.7656805	176.0828402	228.2756213	196.8886391
112.92887570	229.9349112	114.4816568	165.9029586	238.8539645	244.7691124
152.78343200	248.3029586	129.0863905	169.5822485	287.3235503	209.3942012
133.89940830	237.7846154	154.3988166	167.5230769	216.4120710	221.0578698

描述性统计	A	B	C	D	E	F
	Con	DOX	AP39	DOX+AP39	DOX+AP39+CC	DOX+AP39+Genipin
值的数目	10	10	10	10	10	10
最小	99.40	152.5	100.1	142.6	202.9	196.9
最大值	152.8	288.7	158.2	176.1	287.3	267.8
范围	53.39	136.2	58.16	33.45	84.45	70.89
平均: mean	127.7	237.7	128.8	164.9	246.8	230.6
标准偏差 SD	16.04	41.89	18.86	8.844	26.64	22.91
标准误差的平均值	5.072	13.25	5.966	2.797	8.425	7.244

TNNT2

组 A	组 B	组 C	组 D	组 E	组 F
Con	DOX	AP39	DOX+AP39	DOX+AP39+CC	DOX+AP39+Genipin
405.7892308	1993.015385	447.4500000	806.8538462	1730.938462	1911.800000
240.1515385	1841.030769	371.8653846	938.5461538	1896.184615	1819.069231
294.3392308	1894.615385	266.8446154	945.2153846	1964.715385	1558.546154
278.4230769	1625.946154	212.1807692	1150.5615380	1644.184615	1663.015385
353.3276923	1605.300000	309.2053846	900.3538462	1814.815385	2005.123077
198.3915385	1638.923077	174.9192308	779.3000000	1663.661538	1988.176923
234.8476923	1636.638462	193.3592308	924.6538462	1748.076923	1989.153846
360.9746154	1748.230769	346.0123077	860.4846154	1709.184615	1914.392308
183.3469231	1802.900000	264.5630769	1156.4153850	1697.992308	2028.084615
255.9084615	1879.461538	233.9276923	939.6692308	1689.730769	2076.615385

描述性统计	A	B	C	D	E	F
	Con	DOX	AP39	DOX+AP39	DOX+AP39+CC	DOX+AP39+Genipin
值的数目	10	10	10	10	10	10
最小	183.3	1605	174.9	779.3	1644	1559
最大值	405.8	1993	447.5	1156	1965	2077
范围	222.4	387.7	272.5	377.1	320.5	518.1
平均: mean	280.6	1767	282.0	940.2	1756	1895
标准偏差 SD	73.16	136.0	86.49	126.0	104.5	167.9
标准误差的平均值	23.14	43.02	27.35	39.84	33.06	53.10

LDH

组 A	组 B	组 C	组 D	组 E	组 F
Con	DOX	AP39	DOX+AP39	DOX+AP39+CC	DOX+AP39+Genipin
191.0170824	260.3436203	193.2715875	214.1133095	247.0624334	244.8881486
190.5862365	262.1156770	203.8011740	220.4367399	267.8667493	248.6986115
189.0190118	252.0651588	197.7977233	216.6995118	248.1021422	270.3828716
183.3659615	221.4843355	202.9259618	208.4805395	216.4324865	238.2454145
199.8079622	263.0249152	170.3596868	205.3226551	260.5603953	248.6017162
184.0498618	240.4113618	190.9082443	195.9488264	228.2343304	252.9606514
166.7051578	246.1333655	186.3478571	213.0821635	259.1128250	231.7775426
168.8654716	211.5500916	172.0366510	226.0985784	276.0861723	214.9010905
169.5692017	266.1631953	168.2559328	190.3020855	219.3449780	272.6452635
195.1439196	266.3261145	164.6629209	185.7033909	248.2231486	275.3745554

描述性统计	A	B	C	D	E	F
	Con	DOX	AP39	DOX+AP39	DOX+AP39+CC	DOX+AP39+Genipin
值的数目	10	10	10	10	10	10
最小	166.7	211.6	164.7	185.7	216.4	214.9
最大值	199.8	266.3	203.8	226.1	276.1	275.4
范围	33.10	54.78	39.14	40.40	59.65	60.47
平均: mean	183.8	249.0	185.0	207.6	247.1	249.8
标准偏差 SD	11.68	19.27	14.98	13.26	20.18	19.19
标准误差的平均[illegible]	3.694	6.095	4.738	4.193	6.382	6.067

BNP

组 A	组 B	组 C	组 D	组 E	组 F
Con	DOX	AP39	DOX+AP39	DOX+AP39+CC	DOX+AP39+Genipin
360.7166667	1829.733333	937.4666667	1487.8500000	1744.181667	1737.868333
303.2500000	1630.936667	380.8500000	1120.5833330	1782.908333	1650.050000
337.5166667	1873.883333	711.7500000	1233.6633330	1658.383333	1624.963333
784.9000000	1747.891667	753.2633333	966.4466667	1632.008333	1730.978333
763.5333333	1641.463333	321.4666667	1204.3300000	1759.471667	1580.966667
761.2166667	1463.366667	349.3833333	1076.7500000	1668.916667	1733.130000
939.5333333	1520.650000	762.0833333	1256.7000000	1566.250000	1648.088333
905.1666667	1600.283333	797.4166667	1104.8333330	1729.401667	1539.090000
376.2833333	1571.950000	751.7333333	989.0333333	1656.900000	1509.408333
373.4333333	1739.466667	917.0833333	1170.4666670	1773.710000	1693.280000

描述性统计	A	B	C	D	E	F
	Con	DOX	AP39	DOX+AP39	DOX+AP39+CC	DOX+AP39+Genipin
值的数目	10	10	10	10	10	10
最小	660.7	1463	711.8	966.4	1566	1509
最大值	939.5	1874	937.5	1488	1783	1738
范围	278.8	410.5	225.7	521.4	216.7	228.5
平均: mean	820.6	1662	818.2	1161	1697	1645
标准偏差 SD	82.59	133.0	76.35	150.0	71.22	81.82
标准误差的平均[illegible]	26.12	42.05	24.14	47.44	22.52	25.87

G

SOD

组 A	组 B	组 C	组 D	组 E	组 F
Con	DOX	AP39	DOX+AP39	DOX+AP39+CC	DOX+AP39+Genipin
3.678322163	1.387937660	3.143787646	2.203398675	1.366996114	1.182142501
3.111591853	0.820117939	3.880853080	2.543357817	1.264484560	1.000911890
3.652042732	0.749838863	3.347991487	2.119313909	1.279715420	1.195154067
4.034078016	1.251256129	3.948118441	2.593569956	0.951133733	0.857163666
3.162651677	0.942158274	3.246553867	1.491755895	0.982609493	1.112074687
3.074502882	1.037677294	4.034167330	2.067858396	1.148356828	1.078992088
3.159334952	1.100547893	3.409304952	2.191359476	0.916157658	0.805489015
3.035150106	0.788657546	3.637843429	1.080506452	1.060755515	1.498843043
3.508957761	1.060696305	3.454692279	1.513949834	1.025169646	1.121797314
3.539813260	1.106134737	3.169499530	2.101229050	1.281974331	0.745972604

描述性统计	A	B	C	D	E	F
	Con	DOX	AP39	DOX+AP39	DOX+AP39+CC	DOX+AP39+Genipin
值的数目	10	10	10	10	10	10
最小	3.035	0.7498	3.144	1.081	0.9162	0.7460
最大值	4.034	1.388	4.034	2.594	1.367	1.499
范围	0.9989	0.6381	0.8904	1.513	0.4508	0.7529
平均: mean	3.396	1.025	3.527	1.991	1.128	1.060
标准偏差 SD	0.3351	0.2047	0.3294	0.4823	0.1617	0.2213
标准误差的平均[illegible]	0.1060	0.06473	0.1042	0.1525	0.05113	0.06997

GSH-Px

组 A	组 B	组 C	组 D	组 E	组 F
Con	DOX	AP39	DOX+AP39	DOX+AP39+CC	DOX+AP39+Genipin
145.8152685	33.98486467	152.9653459	123.3893363	91.02276048	85.56786226
153.5378311	31.81389199	164.9063344	129.9570277	84.11620892	94.37623060
149.8324862	38.97259257	151.8911975	124.6471271	85.05677744	84.68470195
166.1524679	87.73021173	154.9032524	130.2866259	86.32790231	97.35986088
155.4323022	32.03705744	147.0047923	121.9770463	94.93514229	88.35978583
156.1407627	30.22107353	159.4049666	115.7349609	90.43989544	84.88702798
149.7116814	74.09492237	156.2333824	133.4079081	80.75475434	82.50502621
153.0675469	36.87962799	155.0005829	123.2831431	107.71825100	81.01832113
151.6215625	91.33654947	154.1175024	117.8960328	88.79509818	83.04876744
164.2952842	94.02331780	165.6351552	114.2375566	108.46878960	89.40519021

描述性统计	A	B	C	D	E	F
	Con	DOX	AP39	DOX+AP39	DOX+AP39+CC	DOX+AP39+Genipin
值的数目	10	10	10	10	10	10
最小	145.8	74.09	147.0	114.2	80.75	81.02
最大值	166.2	94.02	165.6	133.4	108.5	97.36
范围	20.34	19.93	18.63	19.17	27.71	16.34
平均: mean	154.6	85.11	156.2	123.5	91.76	87.12
标准偏差 SD	6.383	5.868	5.736	6.387	9.481	5.299
标准误差的平均[illegible]	2.018	1.856	1.814	2.020	2.998	1.676

MDA

组 A	组 B	组 C	组 D	组 E	组 F
Con	DOX	AP39	DOX+AP39	DOX+AP39+CC	DOX+AP39+Genipin
0.451184595	1.342110195	0.238773195	0.768253695	1.415149995	1.009251495
0.387806895	1.283208795	0.365696295	0.648777120	1.461331995	1.775898495
0.391393095	1.297553595	0.334162245	0.772136595	0.958747995	1.090134495
0.280253145	1.042559295	0.486956295	0.632648895	1.255899495	1.600587495
0.326260995	1.102247595	0.412690995	0.763996695	0.960747495	1.276152495
0.394063395	1.496452245	0.374061945	0.484737495	1.052337495	1.197720495
0.389445195	0.884121495	0.424307445	0.721813695	1.603812495	1.668312495
0.326235195	1.201964595	0.377448195	0.632829495	1.116192495	0.879090495
0.246113295	1.263162195	0.404718795	0.641794995	1.274604495	0.786210495
0.215424195	0.895679895	0.181161795	0.567800595	0.761635995	1.055433495

描述性统计	A	B	C	D	E	F
	Con	DOX	AP39	DOX+AP39	DOX+AP39+CC	DOX+AP39+Genipin
值的数目	10	10	10	10	10	10
最小	0.2154	0.8841	0.1812	0.4847	0.7616	0.7862
最大值	0.4512	1.496	0.4870	0.7721	1.604	1.776
范围	0.2358	0.6123	0.3058	0.2874	0.8422	0.9897
平均: mean	0.3408	1.181	0.3600	0.6635	1.186	1.234
标准偏差 SD	0.07523	0.1977	0.09001	0.09426	0.2626	0.3415
标准误差的平均值	0.02379	0.06250	0.02846	0.02981	0.08305	0.1080

NADPH

组 A	组 B	组 C	组 D	组 E	组 F
Con	DOX	AP39	DOX+AP39	DOX+AP39+CC	DOX+AP39+Genipin
4.146118510	15.06427808	4.116976132	9.791839035	19.39587163	19.86603533
2.663095270	15.27371463	3.354741043	10.967377810	19.01637311	14.56859861
3.206309197	18.58662017	2.155888370	12.830547180	15.46100298	18.40632600
3.395669894	22.68649945	3.016559936	9.176999618	15.79387370	19.36478643
3.014358067	20.38554679	4.251290114	11.920398330	20.28568558	13.77592592
3.510685146	19.40066394	3.132093274	9.091256266	17.91284839	19.97742398
4.499453654	21.30722307	3.018502761	11.238984770	17.36626690	22.61578061
3.234415402	15.10183936	4.394929658	10.588138330	13.72023160	14.19816660
2.148376112	14.75355557	3.884096151	10.662483770	15.60736248	15.57498206
3.523896358	18.97971847	2.096049353	9.050845502	17.05800530	20.14709738

描述性统计	A	B	C	D	E	F
	Con	DOX	AP39	DOX+AP39	DOX+AP39+CC	DOX+AP39+Genipin
值的数目	10	10	10	10	10	10
最小	2.148	14.75	2.096	9.051	13.72	13.78
最大值	4.499	22.69	4.395	12.83	20.29	22.62
范围	2.351	7.933	2.299	3.780	6.565	8.840
平均: mean	3.334	18.15	3.342	10.53	17.16	17.85
标准偏差 SD	0.6727	2.918	0.8195	1.273	2.050	3.074
标准误差的平均值	0.2127	0.9226	0.2591	0.4024	0.6483	0.9722

H

p-AMPK/AMPK

组 A	组 B	组 C	组 D
Con	DOX	AP39	DOX+AP39
1.042797992	0.533206950	0.930420736	0.819701062
0.955272719	0.488885360	1.058132790	0.785889071
1.020550449	0.463960885	0.871578728	0.643831701
0.981378840	0.405172339	1.049241789	0.918724810

描述性统计	A	B	C	D
	Con	DOX	AP39	DOX+AP39
值的数目	4	4	4	4
最小	0.9553	0.4052	0.8716	0.6438
最大值	1.043	0.5332	1.058	0.9187
范围	0.08753	0.1280	0.1866	0.2749
平均: mean	1.000	0.4728	0.9773	0.7920
标准偏差 SD	0.03916	0.05341	0.09144	0.1138
标准误差的平均值	0.01958	0.02671	0.04572	0.05688

UCP2

组 A	组 B	组 C	组 D
Con	DOX	AP39	DOX+AP39
0.822966849	0.410369580	0.880657844	0.673042855
1.213663363	0.537145461	0.979687543	0.677313892
0.904982347	0.393380207	0.900379632	0.659221528
1.058387442	0.342245416	0.920516043	0.650091746

描述性统计	A	B	C	D
	Con	DOX	AP39	DOX+AP39
值的数目	4	4	4	4
最小	0.8230	0.3422	0.8807	0.6501
最大值	1.214	0.5371	0.9797	0.6773
范围	0.3907	0.1949	0.09903	0.02722
平均: mean	1.000	0.4208	0.9203	0.6649
标准偏差 SD	0.1727	0.08280	0.04280	0.01254
标准误差的平均值	0.08633	0.04140	0.02140	0.006271

Bax

组 A	组 B	组 C	组 D	组 E
Con	DOX	AP39	DOX+AP39	DOX+AP39+CC
1.093096991	1.828317743	1.074950019	1.086106278	1.311438707
0.985575534	1.865676572	1.018510138	1.173012930	1.418451372
0.921327475	1.648436360	1.016937590	1.096779613	1.411733390

描述性统计	A<br>Con	B<br>DOX	C<br>AP39	D<br>DOX+AP39	E<br>DOX+AP39+CC
值的数目	3	3	3	3	3
最小	0.9213	1.648	1.017	1.086	1.311
最大值	1.093	1.866	1.075	1.173	1.418
范围	0.1718	0.2172	0.05801	0.08691	0.1070
平均: mean	1.000	1.781	1.037	1.119	1.381
标准偏差 SD	0.08679	0.1162	0.03305	0.04740	0.05994
标准误差的平均值	0.05011	0.06706	0.01908	0.02736	0.03461

Bcl-2

组 A	组 B	组 C	组 D	组 E
Con	DOX	AP39	DOX+AP39	DOX+AP39+CC
1.122564812	0.387561824	1.019863545	0.886397010	0.607022940
1.045846831	0.397841051	1.115832013	0.885163716	0.599556617
0.998957747	0.404739778	0.950085917	0.909413604	0.618999490
0.832630611	0.243213503	0.981689405	0.936792384	0.671500074

描述性统计	A<br>Con	B<br>DOX	C<br>AP39	D<br>DOX+AP39	E<br>DOX+AP39+CC
值的数目	4	4	4	4	4
最小	0.8326	0.2432	0.9501	0.8852	0.5996
最大值	1.123	0.4047	1.116	0.9368	0.6715
范围	0.2899	0.1615	0.1657	0.05163	0.07194
平均: mean	1.000	0.3583	1.017	0.9044	0.6243
标准偏差 SD	0.1227	0.07707	0.07188	0.02428	0.03249
标准误差的平均值	0.06133	0.03854	0.03594	0.01214	0.01624

Cleaved Caspase3/Caspase3

组 A	组 B	组 C	组 D	组 E
Con	DOX	AP39	DOX+AP39	DOX+AP39+CC
0.455923569	17.981940300	0.499225833	9.663087128	16.307158250
0.466180772	19.401560610	0.372472568	11.264512450	19.120195860
0.742240609	17.136090440	0.524052344	11.993632360	13.697903130
2.335655050	21.093669730	1.829021440	4.759255976	11.688265700

描述性统计	A<br>Con	B<br>DOX	C<br>AP39	D<br>DOX+AP39	E<br>DOX+AP39+CC
值的数目	4	4	4	4	4
最小	0.4559	17.14	0.3725	4.759	11.69
最大值	2.336	21.09	1.829	11.99	19.12
范围	1.880	3.958	1.457	7.234	7.432
平均: mean	1.000	18.90	0.8062	9.420	15.20
标准偏差 SD	0.9003	1.734	0.6851	3.256	3.224
标准误差的平均值	0.4501	0.8669	0.3426	1.628	1.612

Bax

组 A	组 B	组 C	组 D	组 E
Con	DOX	AP39	DOX+AP39	DOX+AP39+Genipin
1.077753344	2.030310783	1.159942622	1.463518105	1.726870052
0.963562710	1.763272434	1.070417885	1.400009965	1.578629702
0.958683946	2.001435803	1.286185456	1.541592023	1.770433599

描述性统计	A<br>Con	B<br>DOX	C<br>AP39	D<br>DOX+AP39	E<br>DOX+AP39+Genipin
值的数目	3	3	3	3	3
最小	0.9587	1.763	1.070	1.400	1.579
最大值	1.078	2.030	1.286	1.542	1.770
范围	0.1191	0.2670	0.2158	0.1416	0.1918
平均: mean	1.000	1.932	1.172	1.468	1.692
标准偏差 SD	0.06738	0.1466	0.1084	0.07092	0.1005
标准误差的平均值	0.03890	0.08461	0.06259	0.04094	0.05805

Bcl-2

组 A	组 B	组 C	组 D	组 E
Con	DOX	AP39	DOX+AP39	DOX+AP39+Genipin
1.098238286	0.355058102	0.867747347	0.618365378	0.393466489
1.039404182	0.291569481	0.911438564	0.736948211	0.400537703
0.923321490	0.248868179	0.923427602	0.765906652	0.555690514
0.939036042	0.274415146	1.010340952	0.782196352	0.472896560

描述性统计	A<br>Con	B<br>DOX	C<br>AP39	D<br>DOX+AP39	E<br>DOX+AP39+Genipin
值的数目	4	4	4	4	4
最小	0.9233	0.2489	0.8677	0.6184	0.3935
最大值	1.098	0.3551	1.010	0.7822	0.5557
范围	0.1749	0.1062	0.1426	0.1638	0.1622
平均: mean	1.000	0.2925	0.9282	0.7259	0.4556
标准偏差 SD	0.08327	0.04526	0.05974	0.07406	0.07574
标准误差的平均值	0.04163	0.02263	0.02987	0.03703	0.03787

Cleaved Caspase3/Caspase3

组 A	组 B	组 C	组 D	组 E
Con	DOX	AP39	DOX+AP39	DOX+AP39+Genipin
2.054983617	9.305240583	1.033263397	4.018325343	7.884238236
0.794255929	8.879411283	0.642225906	7.192054320	9.033052072
0.320925088	8.699943860	0.337298862	6.034048505	8.363199396
0.829835366	9.623976642	0.921512059	7.422409757	9.729698621

描述性统计	A	B	C	D	E
	Con	DOX	AP39	DOX+AP39	DOX+AP39+Genipin
值的数目	4	4	4	4	4
最小	0.3209	8.700	0.3373	4.018	7.884
最大值	2.055	9.624	1.033	7.422	9.730
范围	1.734	0.9240	0.6960	3.404	1.845
平均: (mean)	1.000	9.127	0.7336	6.167	8.753
标准偏差 (SD)	0.7406	0.4173	0.3112	1.556	0.8040
标准误差的平均值	0.3703	0.2087	0.1556	0.7779	0.4020
