# Supplementary material for: AP39, a novel mitochondria-targeted hydrogen sulfide donor ameliorates doxorubicin-induced cardiotoxicity by regulating the AMPK/UCP2 pathway
Source: PLoS One. 2024 Apr 3;19(4):e0300261. doi: 10.1371/journal.pone.0300261 (PMC10990198; doi:10.1371/journal.pone.0300261)
Supplement: Attachment — Submitted filename: PONE-D-23-34322.pdf [file pone.0300261.s004.pdf]

PLOS ONE

AP39 ameliorates doxorubicin-induced cardiotoxicity by regulating the AMPK/UCP2 pathway

--Manuscript Draft--

**Manuscript Number:**	PONE-D-23-34322
**Article Type:**	Research Article
**Full Title:**	AP39 ameliorates doxorubicin-induced cardiotoxicity by regulating the AMPK/UCP2 pathway
**Short Title:**	AP39 ameliorates doxorubicin-induced cardiotoxicity
**Corresponding Author:**	Bin Liu, M.D.<br>The Second Hospital of Jilin University<br>Changchun, Jilin CHINA
**Keywords:**	AP39; Doxorubicin; Cardiotoxicity; AMPK; UCP2
**Abstract:**	Doxorubicin (DOX) is a broad-spectrum, highly effective antitumor agent, however, its cardiotoxicity has greatly limited its use.Hydrogen sulfide (H2S) is an endogenous gaseous transmitter that exerts cardioprotective effects via the regulation of oxidative stress and apoptosis and maintenance of mitochondrial function, among other mechanisms.AP39 is a novel mitochondria-targeted H2S donor that, at appropriate concentrations, attenuates intracellular oxidative stress damage, maintains mitochondrial function, and ameliorates cardiomyocyte injury.In this study, DOX-induced cardiotoxicity models were established with H9c2 cells and Sprague–Dawley rats to evaluate the protective effect of AP39 and its mechanisms of action.Both in vivo and in vitro experiments showed that DOX induces oxidative stress injury, apoptosis, and mitochondrial damage in cardiomyocytes and decreases the expression of p-AMPK/AMPK and UCP2.All DOX-induced changes were attenuated by AP39 treatment. Furthermore,the protective effect of AP39 was significantly attenuated by the inhibition of AMPK and UCP2. The results suggest that AP39 ameliorates DOX-induced cardiotoxicity by regulating the expression of AMPK/UCP2.
**Order of Authors:**	Bin Zhang
	Yangxue Li
	Ning Liu
	Bin Liu, M.D.
**Additional Information:**	
**Question**	**Response**
**Financial Disclosure**<br><br>Enter a financial disclosure statement that describes the sources of funding for the work included in this submission. Review the submission guidelines for detailed requirements. View published research articles from *PLOS ONE* for specific examples.<br><br>This statement is required for submission and **will appear in the published article** if the submission is accepted. Please make sure it is accurate.	Yes

Powered by Editorial Manager® and ProduXion Manager® from Aries Systems Corporation

**Funded studies**<br>Enter a statement with the following details:<br>• Initials of the authors who received each award<br>• Grant numbers awarded to each author<br>• The full name of each funder<br>• URL of each funder website<br>• Did the sponsors or funders play any role in the study design, data collection and analysis, decision to publish, or preparation of the manuscript?<br><br>Did you receive funding for this work?	
Please add funding details.<br>as follow-up to "**Financial Disclosure**<br><br>Enter a financial disclosure statement that describes the sources of funding for the work included in this submission. Review the submission guidelines for detailed requirements. View published research articles from *PLOS ONE* for specific examples.<br><br>This statement is required for submission and **will appear in the published article** if the submission is accepted. Please make sure it is accurate.<br><br>**Funded studies**<br>Enter a statement with the following details:<br>• Initials of the authors who received each award<br>• Grant numbers awarded to each author<br>• The full name of each funder<br>• URL of each funder website<br>• Did the sponsors or funders play any role in the study design, data collection and analysis, decision to publish, or preparation of the manuscript?<br><br>Did you receive funding for this work?"	This work was supported by Jilin Province Science and Technology Department (20220303002SF), Jilin Provincial Development and Reform Commission (2022C003), Jilin Province Science and Technology Department (20190905002SF).
Please select the country of your main research funder (please select carefully as in some cases this is used in fee calculation).	CHINA - CN

Powered by Editorial Manager® and ProduXion Manager® from Aries Systems Corporation

as follow-up to "**Financial Disclosure**<br><br>Enter a financial disclosure statement that describes the sources of funding for the work included in this submission. Review the submission guidelines for detailed requirements. View published research articles from *PLOS ONE* for specific examples.<br><br>This statement is required for submission and **will appear in the published article** if the submission is accepted. Please make sure it is accurate.<br><br>**Funded studies**<br>Enter a statement with the following details:<br>• Initials of the authors who received each award<br>• Grant numbers awarded to each author<br>• The full name of each funder<br>• URL of each funder website<br>• Did the sponsors or funders play any role in the study design, data collection and analysis, decision to publish, or preparation of the manuscript?<br><br>Did you receive funding for this work?"	
**Competing Interests**<br><br>Use the instructions below to enter a competing interest statement for this submission. On behalf of all authors, disclose any competing interests that could be perceived to bias this work—acknowledging all financial support and any other relevant financial or non-financial competing interests.<br><br>This statement is required for submission and **will appear in the published article** if the submission is accepted. Please make sure it is accurate and that any funding sources listed in your Funding Information later in the submission form are also declared in your Financial Disclosure statement.	The authors have declared that no competing interests exist.

View published research articles from *PLOS ONE* for specific examples.<br><br>**NO authors have competing interests**<br><br>Enter: *The authors have declared that no competing interests exist.*<br><br>**Authors with competing interests**<br><br>Enter competing interest details beginning with this statement:<br><br>*I have read the journal's policy and the authors of this manuscript have the following competing interests: [insert competing interests here]*<br><br>* typeset	
**Ethics Statement**<br><br>Enter an ethics statement for this submission. This statement is required if the study involved:<br><br>• Human participants<br>• Human specimens or tissue<br>• Vertebrate animals or cephalopods<br>• Vertebrate embryos or tissues<br>• Field research<br><br>Write "N/A" if the submission does not require an ethics statement.<br><br>General guidance is provided below. Consult the submission guidelines for detailed instructions. **Make sure that all information entered here is included in the Methods section of the manuscript.**	The study was approved by the Institutional Committee for the Protection and Utilization of Animals of Jilin University(2023 No. 463).Rats were anesthetized with isoflurane inhalation anesthesia and euthanasia by CO2 inhalation.

Powered by Editorial Manager® and ProduXion Manager® from Aries Systems Corporation

**Format for specific study types**<br><br>**Human Subject Research (involving human participants and/or tissue)**<br>• Give the name of the institutional review board or ethics committee that approved the study<br>• Include the approval number and/or a statement indicating approval of this research<br>• Indicate the form of consent obtained (written/oral) or the reason that consent was not obtained (e.g. the data were analyzed anonymously)<br><br>**Animal Research (involving vertebrate animals, embryos or tissues)**<br>• Provide the name of the Institutional Animal Care and Use Committee (IACUC) or other relevant ethics board that reviewed the study protocol, and indicate whether they approved this research or granted a formal waiver of ethical approval<br>• Include an approval number if one was obtained<br>• If the study involved *non-human primates*, add *additional details* about animal welfare and steps taken to ameliorate suffering<br>• If anesthesia, euthanasia, or any kind of animal sacrifice is part of the study, include briefly which substances and/or methods were applied<br><br>**Field Research**<br><br>Include the following details if this study involves the collection of plant, animal, or other materials from a natural setting:<br>• Field permit number<br>• Name of the institution or relevant body that granted permission	
**Data Availability**<br><br>Authors are required to make all data underlying the findings described fully available, without restriction, and from the time of publication. PLOS allows rare exceptions to address legal and ethical concerns. See the PLOS Data Policy and FAQ for detailed information.	Yes - all data are fully available without restriction

A Data Availability Statement describing where the data can be found is required at submission. Your answers to this question constitute the Data Availability Statement and **will be published in the article**, if accepted.<br><br>**Important:** Stating 'data available on request from the author' is not sufficient. If your data are only available upon request, select 'No' for the first question and explain your exceptional situation in the text box.<br><br>Do the authors confirm that all data underlying the findings described in their manuscript are fully available without restriction?	
**Describe where the data may be found in full sentences. If you are copying our sample text, replace any instances of XXX with the appropriate details.**<br><br>• If the data are **held or will be held in a public repository**, include URLs, accession numbers or DOIs. If this information will only be available after acceptance, indicate this by ticking the box below. For example: *All XXX files are available from the XXX database (accession number(s) XXX, XXX.)*.<br>• If the data are all contained **within the manuscript and/or Supporting Information files**, enter the following: *All relevant data are within the manuscript and its Supporting Information files.*<br>• If neither of these applies but you are able to provide **details of access elsewhere**, with or without limitations, please do so. For example:<br><br>*Data cannot be shared publicly because of [XXX]. Data are available from the XXX Institutional Data Access / Ethics Committee (contact via XXX) for researchers who meet the criteria for access to confidential data.*<br><br>*The data underlying the results presented in the study are available from (include the name of the third party*	All relevant data are within the manuscript and its Supporting Information files.

and contact information or URL).<br>• This text is appropriate if the data are owned by a third party and authors do not have permission to share the data.<br><br>* typeset	
Additional data availability information:	

Powered by Editorial Manager® and ProduXion Manager® from Aries Systems Corporation

Manuscript

Click here to access/download;Manuscript;AP39 ameliorates doxorubicin induced cardiotoxicity.docx

AP39 ameliorates doxorubicin-induced cardiotoxicity by regulating the AMPK/UCP2 pathway

First author:Bin Zhang

Corresponding author:Bin Liu

Authors:Bin Zhang[a],Yangxue Li[a],Ning Liu[a],Bin Liu[a,*]

a The Second Hospital of Jilin University,Changchun, Jilin,130000, China.

*Corresponding author at:No. 4026 Yatai Street, Nanguan District, Changchun City, Jilin Province, China.The Second Hospital of Jilin University.E-mail address:liubin3333@vip.sina.com

Abstract

Doxorubicin (DOX) is a broad-spectrum, highly effective antitumor agent, however, its cardiotoxicity has greatly limited its use.Hydrogen sulfide (H_2S) is an endogenous gaseous transmitter that exerts cardioprotective effects via the regulation of oxidative stress and apoptosis and maintenance of mitochondrial function, among other mechanisms.AP39 is a novel mitochondria-targeted H_2S donor that, at appropriate concentrations, attenuates intracellular oxidative stress damage, maintains mitochondrial function, and ameliorates cardiomyocyte injury.In this study, DOX-induced cardiotoxicity models were established with H9c2 cells and Sprague–Dawley rats to evaluate the protective effect of AP39 and its mechanisms of action.Both in vivo and in vitro experiments showed that DOX induces oxidative stress injury, apoptosis, and mitochondrial damage in cardiomyocytes and decreases the expression of p-AMPK/AMPK and UCP2.All DOX-induced changes were attenuated by AP39 treatment. Furthermore,the protective effect of AP39 was significantly

attenuated by the inhibition of AMPK and UCP2. The results suggest that AP39 ameliorates DOX-induced cardiotoxicity by regulating the expression of AMPK/UCP2.

Key words:AP39,Doxorubicin,Cardiotoxicity,AMPK,UCP2

1 Introduction

Doxorubicin (DOX), a broad-spectrum anthracycline antineoplastic drug, is widely used for the treatment of leukemia, breast cancer, ovarian cancer, lymphoma, and osteosarcoma (1).However, its severe dose-dependent cardiotoxicity (2)affects the quality of life of patients with cancer and can even shorten life expectancy.There is evidence that DOX exerts cardiotoxicity via oxidative stress, apoptosis, inflammation, and fibrosis.Furthermore,due to its cationic nature,DOX readily binds to mitochondrial intramembranous membranes and forms an irreversible complex with cardiac phospholipid proteins, leading to cardiotoxicity by inducing mitochondrial damage in cardiomyocytes (3, 4).The only drug currently approved by the FDA for the treatment of DOX cardiotoxicity is dexrazoxane, which still has various side effects, including myelotoxicity in patients with soft-tissue sarcoma (5). Therefore, there is an urgent need to identify safe and effective drugs to improve DOX cardiotoxicity.

DOX-induced cardiotoxicity is related to adenosine

monophosphate-activated protein kinase (AMPK) (6). AMPK is an important regulator of cellular energy homeostasis and mitochondrial homeostasis. The activation of AMPK modulates cellular metabolism, autophagy, apoptosis, and fibrosis (7). Uncoupling protein 2 (UCP2) is located within the inner mitochondrial membrane and affects mitochondrial function and metabolism through oxidative phosphorylation uncoupling. AMPK attenuates oxidative stress damage, reduces apoptosis (8), attenuates mitochondrial damage (9), and attenuates inflammatory responses (10) by upregulating UCP2.

Hydrogen sulfide (H_2S), a gaseous signaling molecule, exerts a potent protective effect on the cardiovascular system by regulating oxidative stress, apoptosis, autophagy, inflammation, mitochondrial function, neovascularization, and fibrosis at reasonable concentrations and has been shown to function via AMPK(11, 12).AP39 is a novel mitochondria-targeted H_2S donor that attenuates intracellular oxidative stress at appropriate concentrations while maintaining cell viability, mitochondrial respiration, and mitochondrial DNA integrity (13, 14).AP39 prevents myocardial ischemia-reperfusion injury independently of the cytoplasmic RISK pathway (15),it also inhibits mitochondrial autophagy, antagonizes cardiomyocyte iron death, and ameliorates myocardial fibrosis in rats with myocardial infarction via the PINK1/Parkin pathway (16).

Based on its effects on oxidative stress, apoptosis, and mitochondrial processes, we hypothesized that the exogenous H_2S donor AP39 may

attenuate DOX-induced cardiotoxicity. The aim of this study was to assess whether AP39 exerts a protective effect against DOX-induced cardiotoxicity and to investigate its mechanism of action,including its effects on the mitochondrial pathway and AMPK/UCP2.

2. Materials and methods

2.1 Reagents and antibodies

DOX (S1208) was purchased from Selleck (Houston, TX, USA), AP39(HY-126124) was purchased from MCE , Compound C(CC; 171260) and genipin (G4796)were purchased from Sigma–Aldrich (St. Louis, MO, USA).Primary antibodies for the following proteins were purchased from Cell Signaling Technology (Danvers, MA, USA):Caspase-3(9662, 1:1000),Cleaved Caspase-3 (9664, 1:1000),AMPKα (5831, 1:1000), p-AMPKα (Thr172) (50081, 1:1000), UCP2 (89326, 1:1000).Primary antibodies for Bax(A0207, 1:1000) and Bcl-2 (A19693, 1:1000) were purchased from ABclonal.Small interfering RNA against UCP2 (siUCP2) and its negative control (NC) were synthesized by IBSBIO.Lipofectamine 2000 was purchased from Invitrogen(Waltham, MA, USA).Annexin V-FITC and SYTOX Red were purchased from Thermo Fisher Scientific(Waltham, MA, USA).The Cell Counting Kit-8(CCK-8) and BCA Protein Assay Kit were purchased from Bioss.

Additionally,2′,7′-dichlorofluorescein diacetate (DCFH-DA) was purchased from Bestbio.The Mitochondrial Membrane Potential Assay Kit with JC-1 and

H2S Content Assay Kit were purchased from Solarbio (Beijing, China).The ATP assay kit was purchased from Nanjing Jiancheng Bioengineering Institute.The Superoxide Dismutase (SOD) Activity Assay Kit,Glutathione Peroxidase (GPX) Activity Assay Kit,Malondialdehyde (MDA) Content Assay Kit,Coenzyme II NADP (H) Content Assay Kit, and Lactate Dehydrogenase (LDH) Activity Assay Kit were all purchased from Beijing Boxbio Science & Technology Co., Ltd. (Beijing, China). The Rat Troponin T Type 2, Cardiac (TNNT2) ELISA Kit, Rat Creatine Kinase MB Isoenzyme (CKMB) ELISA Kit, and Rat Brain Natriuretic Peptide (BNP) ELISA Kit were all purchased from Jianglai Biology (Shanghai, China).

2.2 Animals and treatment

The study was conducted according to the guidelines of the Declaration of Helsinki, and approved by the Institutional Committee for the Protection and Utilization of Animals of Jilin University(2023 No. 463).

Male 8 to 10-week-old SPF Sprague–Dawley rats, weighing 300–320 g, were purchased from Yeast Laboratory Animal Technology.The rats were housed at the Animal Center of Jilin University, with the room temperature controlled at 21 ± 1°C, humidity controlled at 50-60%, 12h day/night cycle, free access to water and food, and acclimatization for 1 week. They were then randomly assigned to groups (10 rats per group) and administered treatments according to different protocols.The specific dosing regimens were as follows: (1) Con (equal amounts of 0.9% NaCl + DMSO); (2) DOX(5mg/kg once a

week,ip, cumulative dose 15mg/kg); (3) AP39(50nmol/kg every other day,ip); (4) DOX + AP39; (5) DOX + AP39 + CC(20mg/kg/d for 1 week,ip); (6) DOX + AP39 + Genipin(20mg/kg/d for 3 days,ip).The above doses were based on previous study reports (17,18,19,20) and experimental data.

Weighing was performed every 3 days during the experiment, and the rats' mental status, activity status, and any pain or discomfort were also paid attention to and recorded. The duration of this experiment was 21 days, and no rats died before euthanasia. 21 days later, cardiac ultrasound was performed after isoflurane anesthesia was given, and then euthanasia was given by CO_2 inhalation method (a total of 60 rats). The above experiments were supervised and directed by the Institutional Committee for the Protection and Utilization of Animals of Jilin University.

2.3 Cell culture and treatments

The rat H9c2 cell line was purchased from Beijing Zhongke QC Biotechnology Co. (Beijing, China). DMEM supplemented with 10% fetal bovine serum and 1% penicillin and streptomycin was used for cell culture in an incubator at 37°C and a CO_2 concentration of 5%.Different drugs were given to stimulate the cells for 24h according to the experimental protocol including DOX (1 μmol/L), AP39 (100 nmol/L), and CC (10 μmol/L) (21). To reduce UCP2 expression in vitro, cells were transfected with siUCP2 (50 nmol/L) using the transfection reagent Lipofectamine2000 for 48h, and the effectiveness of transfection was evaluated by qPCR and western blotting.

2.4 Cell activity assay

H9c2 cells were inoculated in 96-well plates (4×10^3/well) and incubated with different concentrations of DOX (0, 0.5, 1, and 2 μmol/L) and AP39 (0, 30, 50, 100, 300, and 500 nmol/L), with a final volume of 100μL in each well.After 24 h, 10 μL of CCK-8 reagent was added to each well, the cells were incubated in the cell incubator for 60 min, and absorbance was measured at 450 nm.

2.5 Detection of ROS

H9c2 cells were inoculated in 6-well plates (5×10^4/well), and different stimuli were applied when cells reached approximately 70% confluence. Cells were incubated for 24h in a cell culture incubator. The DCFH-DA probe was diluted with serum-free DMEM at a ratio of 1:1000 and added to the 6-well plates at 1 mL/well, followed by incubation 37°C in the dark for 20 min. Cells were washed gently with phosphate-buffered saline and images were obtained under a fluorescence microscope. The average fluorescence intensity was evaluated using ImageJ.

2.6 Flow cytometry

H9c2 cells were resuspended under different conditions and diluted with 1× Binding Buffer to a concentration of 1×10^6 cells/mL. Then, 100 μL of the cell suspension was used for flow cytometry; briefly, 5 μL of Annexin V-FITC and 5 μL of SYTOX Red were added, samples were incubated at room temperature (25°C) in the dark for 15 min, 400 μL of 1× Binding Buffer was added, and samples were assayed immediately using the flow

cytometer(Cytoflex,Beckman).

2.7 Western blotting

Total protein was extracted from cell samples and cardiac tissues using RIPA buffer, and the protein concentration was determined using a BCA Kit. Equal concentrations of protein samples were separated by 10% SDS-PAGE and then transferred to PVDF membranes, which were blocked with 5% skim milk powder at room temperature for 60 min. The primary antibody was incubated overnight at 4°C, followed by incubation with the secondary antibody at room temperature for 1 h. Chemiluminescent color development was performed by adding the developing solution.

2.8 Mitochondrial membrane potential assay

Mitochondrial membrane potential was assayed using the JC-1 probe according to the manufacturer's instructions. When the mitochondrial membrane potential was high, JC-1 aggregated in the mitochondrial matrix and formed a polymer, producing red fluorescence; when the mitochondrial membrane potential was low, JC-1 did not aggregate in the mitochondrial matrix, and the monomers produced green fluorescence. Images were obtained using a fluorescence microscope, and the fluorescence intensity was analyzed using ImageJ. The ratio of red to green fluorescence was used to measure the change in mitochondrial membrane potential.

2.9 Quantitative real-time PCR

Total RNA was extracted with TransZol, and reverse transcription and

qRT-PCR were performed according to the instructions provided with the relevant kits. *GAPDH* was selected as the internal reference gene, and the relative expression was calculated by the $2^{-\Delta\Delta CT}$ method.

2.10 Oxidative stress and ATP assays

According to the manufacturer's instructions, oxidative stress levels were measured using SOD, GSH-Px, MDA and NADPH kits, and cellular ATP levels were measured using ATP kits.Absorbance values were measured at different wavelengths using an enzyme meter and analyzed according to the standard curves and corresponding formulas.

2.11 ELISA

Cardiomyocyte injury was assessed using ELISA kits for TNNT2, CK-MB, and BNP in rat serum according to the manufacturer's instructions.

2.12 Transmission electron microscopy

Different groups of rat myocardial specimens and different drug-stimulated H9c2 cells were fixed with 2.5% glutaraldehyde phosphate and stained with1% phosphotungstic acid. The mitochondrial ultrastructure was observed and analyzed by using a JEM-1400 microscope at a magnification of 5000×, 8000×, and 25000×.

2.13 HE and Masson staining

Rat myocardial tissues were fixed with 4% paraformaldehyde, embedded in paraffin, and cut into 3-μm-thick wax slices. The sections were stained with hematoxylin and eosin (HE), Masson Lichtenstein acidic reagent, and toluidine

blue and observed under a light microscope.

2.14 Statistical analyses

All statistical analyses were performed using GraphPad Prism 9.0. Datas are expressed as the mean ± standard deviation (SD). Comparisons between two groups were performed using Student's *t*-test, comparisons among multiple groups were performed using one-way ANOVA and post hoc Tukey's tests. Values of $p < 0.05$ were statistically significant . All data used in statistical analyses were obtained from three or more independent repeated experiments.

3 Results

3.1 DOX induces H9c2 cell damage

H9c2 cells were stimulated with various concentrations of DOX (0, 0.5, 1, or 2 μmol/L) for 24 h for CCK-8 detection.Exposure to 1 μmol/L DOX for 24 h decreased H9c2 cell viability by approximately 50% (compared with that in the control group), and the DOX-induced decrease in cell viability was dose-dependent. We stimulated H9c2 cells with 1 μmol/L DOX for different durations (0, 6, 12, 24, and 48 h). A CCK-8 assay showed that cell viability decreased by about 50% at 24 h. Therefore, we stimulated H9c2 cells with 1 μmol/L DOX for 24 h for subsequent experiments (Fig. 1 A, B).

Free radical production is the main cause of cardiomyocyte damage by DOX, and cardiotoxicity occurs progressively with ROS production and lipid

peroxidation (22). As determined using the DCFH-DA probe, DOX increased ROS levels in cardiomyocytes (Fig. 1 C), resulting in decreased SOD and GSH-Px activity and increased MDA and NADPH levels (Fig. 1 D), suggesting that DOX causes oxidative stress injury in cardiomyocytes. Flow cytometry revealed that the apoptosis rate was significantly higher($p < 0.01$) in the DOX group than in the Con group (Fig. 1 E), suggesting that DOX caused apoptosis in H9c2 cells.

Figure1.DOX induces H9c2 cell damage.

3.2 DOX induces mitochondrial damage in H9c2 cells

Previous studies have shown that DOX can lead to cardiomyocyte apoptosis via endogenous pathways (23), particularly the mitochondrial pathway. Furthermore, DOX can lead to mitochondrial damage (4). In this study, DOX increased the expression levels of the apoptosis-related protein Bax, decreased expression levels of Bcl-2, and increased expression levels of Cleaved Caspase-3/Caspase-3 (Fig. 2 A), indicating that DOX promotes apoptosis in cardiomyocytes and its mechanism of action involves mitochondria. We further evaluated mitochondrial membrane potential and ATP levels, revealing that DOX could lead to a decrease in mitochondrial membrane potential and ATP levels in cardiomyocytes (Fig. 2 B, C), while mitochondrial damage (mitochondrial structural disorganization, fragmentation, and cristae rupture) was observed by transmission electron microscopy (Fig. 2

D).

We performed AMPK and UCP2 assays. Western blotting showed that DOX resulted in decreased levels of p-AMPK/AMPK and UCP2 in cardiomyocytes (Fig. 2 E), suggesting that the damage to cardiomyocytes caused by DOX may be related to AMPK/UCP2.

Figure2.DOX induces mitochondrial damage in H9c2 cells.

3.3 AP39 ameliorates DOX-induced myocardial injury

AP39 has a concentration-dependent effect on mitochondrial activity. At low concentrations (30–100 nmol/L), AP39 stimulates mitochondrial electron transport and cellular bioenergetic functions, and at high concentrations (300 nmol/L), it has an inhibitory effect on mitochondrial activity (14). Therefore, we first stimulated H9c2 cells with different concentrations of AP39 (0, 30, 50, 100, 300, and 500 nmol/L) for 24 h and performed CCK-8 assays. The results were in accordance with those of previous reports indicating that AP39 at lower concentrations (30–100 nmol/L) does not significantly reduce cell viability. A decrease in cell viability was detected at 300 nmol/L, and a significant decrease in cell viability was detected at 500 nmol/L. Subsequently, we co-stimulated H9c2 cells with 1 μmol/L DOX and different concentrations of AP39 for 24 h. The CCK-8 results showed that the improvement of cell viability was statistically significant at AP39 concentrations of 50 nmol/L and 100 nmol/L, and the improvement was particularly obvious at an AP39

concentration of 100 nmol/L. In summary, we chose 100 nmol/L AP39 for subsequent experiments (Fig. 3 A–C).

We measured the intracellular H_2S content under different conditions, demonstrating that DOX stimulation decreases the H_2S content in H9c2 cells, and this decrease was attenuated by the exogenous administration of AP39 (Fig. 3 D). In addition, AP39 significantly ameliorated DOX-induced oxidative stress injury in H9c2 cells, with a significant decrease in intracellular ROS levels (Fig. 3 E), improvements in SOD and GSH-Px activity, and decreases in MDA and NADPH levels after co-treatment with AP39 compared with corresponding levels in the DOX group (Fig. 3 F). AP39 ameliorated DOX-induced cardiomyocyte apoptosis, which was significantly lower in the DOX+AP39 group than in the DOX group (Fig. 3 G).

Figure3.AP39 ameliorates DOX-induced myocardial injury.

3.4 AP39 ameliorates DOX-induced mitochondrial damage

We further investigated the mechanisms by which AP39 exerted protective effects against DOX-induced myocardial injury. Western blotting showed that AP39 decreased the expression of the apoptosis-related proteins Bax and Cleaved Caspase-3/Caspase-3 and increased the expression of Bcl-2 (Fig. 4 A). Additionally, AP39 attenuated the DOX-induced decrease in mitochondrial membrane potential and ATP levels in cardiomyocytes (Fig. 4 B, C). Transmission electron microscopy revealed that mitochondrial damage (i.e.,

the disorganization of mitochondrial structure, fragmentation, and cristae breakage) was attenuated by AP39 (Fig. 4 D).

As determined by western blotting, cardiomyocyte p-AMPK/AMPK and UCP2 levels were elevated after co-treatment with AP39 and DOX than after DOX stimulation alone (Fig.4 E), suggesting that the beneficial effect of AP39 on DOX cardiotoxicity may be related to AMPK/UCP2.

Figure4.AP39 ameliorates DOX-induced mitochondrial damage.

3.5 Inhibition of AMPK expression limits the beneficial effect of AP39 on DOX cardiotoxicity

To verify whether the beneficial effect of AP39 on DOX cardiotoxicity was related to AMPK, we inhibited AMPK using the AMPK inhibitor Compound C (CC) and demonstrated the effectiveness of CC by western blotting (Fig. 5 A). As determined by a CCK-8 assay, CC did not influence cell viability (Fig. 5 B). ROS levels were significantly higher in the DOX+AP39+CC group than in the DOX+AP39 group (Fig. 5 C). SOD and GSH-Px activities were lower and MDA and NADPH levels were higher in the DOX+AP39+CC group than in the DOX+AP39 group (Fig. 5 D), suggesting that the inhibition of AMPK expression limited the beneficial effect of AP39 on DOX-induced oxidative stress injury in cardiomyocytes. The apoptosis rate was higher in the DOX+AP39+CC group than in the DOX+AP39 group (Fig. 5 E). Western blotting showed that the expression levels of Bax and Cleaved

Caspase-3/Caspase-3 were higher and expression levels of Bcl-2 were lower in DOX+AP39+CC group than in the DOX+AP39 group (Fig. 5 F), suggesting that the inhibition of AMPK limited the effect of AP39 on DOX-induced apoptosis in cardiomyocytes. Furthermore, the beneficial effects of AP39 on both mitochondrial membrane potential and ATP levels in cardiomyocytes were weakened by the inhibition of AMPK expression (Fig. 5 G, H). These results suggest that AP39 ameliorates DOX-induced cardiotoxicity by regulating AMPK expression.

We further evaluated the regulatory relationship between AMPK and UCP2. Although the down-regulation of UCP2 by DOX was improved by co-treatment with AP39, the expression level of UCP2 in the DOX+AP39+CC group was still significantly lower than that in the DOX+AP39 group (Fig. 5 I), indicating that the inhibition of AMPK expression suppressed the up-regulation of UCP2 by AP39. These findings suggest that UCP2 may function downstream of AMPK in the regulation of DOX cardiotoxicity by AP39.

Figure5.Inhibition of AMPK expression limits the beneficial effect of AP39 on DOX cardiotoxicity.

3.6 AP39 improves DOX-induced cardiotoxicity by preventing the down-regulation of UCP2

To clarify whether the beneficial effect of AP39 on DOX cardiotoxicity was achieved by modulating the expression of UCP2, we inhibited the expression

of UCP2 using small interfering RNA and confirmed the effectiveness of transfection by qPCR and western blotting (Fig. 6 A). CCK-8 results showed that cell viability did not differ significantly in the NC and siUCP2 groups compared with Con group(Fig. 6 B). Oxidative stress damage, apoptosis, and mitochondrial damage were not significantly improved in the DOX+AP39+siUCP2 group compared with those in the DOX+AP39 group. In particular, ROS levels were high (Fig. 6 C), SOD and GSH-Px levels were low, and MDA and NADPH levels were high (Fig. 6 D). The apoptosis rate remained high, and western blotting showed that Bax and Cleaved Caspase-3/Caspase-3 levels were high and Bcl-2 levels were low (Fig. 6 E, F). Mitochondrial membrane potential and ATP levels remained low (Fig. 6 G, H). The above results suggested that the inhibition of UCP2 inhibited the beneficial effect of AP39 on DOX cardiotoxicity, suggesting that UCP2 mediates the effects of AP39.

We hypothesized that UCP2 acts downstream of AMPK. To verify this, we further evaluated the levels of p-AMPK/AMPK. DOX decreased the expression of p-AMPK/AMPK. AP39 upregulated p-AMPK/AMPK, and the inhibition of UCP2 did not influence the effect of AP39 (Fig. 6 I). We have previously confirmed that the inhibition of AMPK could affect the expression of UCP2. These findings further demonstrated that UCP2 functions downstream of AMPK.

Collectively, these findings demonstrated that AP39 ameliorates

DOX-induced oxidative stress damage, apoptosis, and mitochondrial damage in H9c2 cells by regulating the expression of AMPK/UCP2.

Figure6.AP39 improves DOX-induced cardiotoxicity by preventing the down-regulation of UCP2.

3.7 AP39 attenuates DOX-induced cardiotoxicity in rats by regulating the AMPK/UCP2 pathway

To further validate our experimental results, we conducted in vivo experiments with rats. DOX administration resulted in a significant decrease in body weight and an elevated heart/body weight ratio in rats over those in the control group (Fig. 7 A, B). Cardiac ultrasound showed a significant decrease in EF%, FS%, and E/A, suggesting that there was a significant decline in cardiac function (Fig. 7 C). The levels of TNNT2, CK-MB, LDH, and BNP were significantly increased in abdominal aorta blood after DOX administration (Fig. 7 D), indicating obvious myocardial damage. HE staining of the rat myocardium was observed under an optical microscope; myocardial cells in the DOX group were deformed, broken, and dissolved, with edema, an enlarged myocardial interstitial space, unevenly colored myocardial fibers, and inflammatory cell infiltration. Masson staining showed a disrupted arrangement of cardiomyocytes, obvious increase in blue collagen fibers in the interstitium of the myocardium, and obvious myocardial fibrosis in the DOX group (Fig. 7 E). Mitochondrial swelling, structural disorder, fragmentation, ridge breakage,

and vacuole-like degeneration of cardiomyocytes in the DOX group were observed by transmission electron microscopy (Fig. 7 F). We also tested indexes of serum oxidative stress in rats. SOD and GSH-Px activities were lower and MDA and NADPH levels were higher in the DOX group than in the control group (Fig. 7 G). Western blotting showed that DOX increased the expression of the apoptosis-related protein Bax, decreased the expression of Bcl-2, increased the expression of Cleaved Caspase-3/Caspase-3, and decreased expression levels of p-AMPK/AMPK and UCP2 (Fig. 7H). In the DOX+AP39 group, the toxic effects of DOX were ameliorated to varying degrees, consistent with the results of our in vitro experiments. We also confirmed the mechanism by which AP39 improves DOX cardiotoxicity in vivo by administering the AMPK inhibitor CC and UCP2 inhibitor genipin.Cardiomyocyte injury, oxidative stress injury, mitochondrial injury, and apoptosis, as described above, did not differ significantly in the DOX+AP39+CC and DOX+AP39+Genipin groups, also consistent with results of in vitro experiments. These findings suggest that the beneficial effect of AP39 on DOX-induced cardiotoxicity in rats is achieved by modulating AMPK/UCP2 expression.

Figure7.AP39 attenuates DOX-induced cardiotoxicity in rats by regulating the AMPK/UCP2 pathway.

4 Discussion

DOX is a broad-spectrum and highly effective antitumor drug commonly used in the treatment of different types of tumors. It can significantly improve the survival rate of patients with cancer. However, its severe cardiotoxicity greatly limits its application. Therefore, there is an urgent need to find drugs that can reduce the cardiotoxicity of DOX. In this study, both in vivo and in vitro experiments demonstrated that the exogenous mitochondria-targeted H_2S donor AP39 could attenuate DOX-induced cardiotoxicity by ameliorating oxidative stress, apoptosis, and mitochondrial damage. Mechanistically, we found that AP39 exerts its protective effects by activating the expression of AMPK/UCP2, and inhibitors of AMPK and UCP2 can attenuate or even eliminate the beneficial effect of AP39.These results clearly indicate that AP39 is promising for the prevention or treatment of DOX cardiotoxicity.

Increasing focus on DOX cardiotoxicity has led to extensive research. Studies have shown that DOX decreases levels of SOD, CAT, and GSH-Px and increases levels of MDA in the rat heart, and the amelioration of oxidative stress injury can ameliorate cardiotoxicity (24, 25), consistent with our findings. In our experiments, DOX induce ROS production in H9c2 cardiomyocytes, decreased SOD and GSH-Px activity in cardiomyocytes and rat serum, and increased MDA and NADPH levels, indicating that it induces oxidative stress in cardiomyocytes. DOX can induce cardiomyocyte apoptosis through both endogenous and exogenous pathways (23). For example, DOX can induce apoptosis and pyroptosis via the Akt/mTOR signaling pathway (26), heat shock

proteins (HSP-10, HSP-20, HSP-22, HSP-27, and HSP60), and lipocalin, and it is possible to reduce the cardiotoxicity of DOX by promoting antiapoptotic activity (27, 28), as demonstrated in our experiments. In particular, we found that DOX can significantly increase the apoptosis rate of H9c2 cells, up-regulate Bax and Cleaved Caspase-3/Caspase-3, and down-regulate Bcl-2, suggesting that DOX can induce endogenous apoptosis via the mitochondrial pathway. Compared with other cell types, cardiomyocytes have more mitochondria, and DOX mainly acts on cardiomyocyte mitochondria, interfering with mitochondrial electron transport and leading to the formation of superoxide (O2-) free radicals (29). DOX induces mitochondrial DNA (mtDNA) mutations and defects along with elevated ROS in mitochondria, and these changes have been implicated in the development of cardiomyopathy (30). DOX can also induce excessive opening of mitochondrial permeability transition pore (31) and affect mitochondrial KATP channel activity (32), thus leading to myocardial injury. In our experiments, DOX decreased mitochondrial membrane potential and ATP levels in cardiomyocytes. Mitochondrial structure disorganization, fragmentation, and cristae rupture were observed. These in vivo and in vitro experiments clearly show that DOX causes the structural damage and dysfunction of mitochondria in cardiomyocytes.

Hydrogen sulfide (H_2S), initially described as a toxic gas with a rotten egg odor, is similar in nature to nitric oxide (NO) and carbon monoxide (CO), an endogenous gaseous signaling molecule in mammals. Increasing studies have

shown that it is involved in a variety of pathophysiological processes, such as oxidative stress, inflammation, apoptosis, and angiogenesis; additionally, it plays a protective role in the pathogenesis and progression of cardiovascular diseases (33). H_2S reduces lipid peroxidation by hydrogen peroxide and superoxide scavenging in a model of isoprenaline-induced myocardial injury (34). H_2S-mediated activation of Nrf2-dependent pathways leads to the upregulation of genes involved in endogenous antioxidant defense (35). It protects mitochondrial function by inhibiting respiration, thereby limiting ROS production and reducing mitochondrial uncoupling (36). Furthermore, H_2S significantly prevents high glucose-induced apoptosis in cardiomyocytes by modulating the expression of Bax and Bcl-2 (37). AP39, a novel mitochondria-targeted H_2S donor, can ameliorate high-fat-diet-induced liver injury in young rats by attenuating oxidative stress and mitochondrial damage (38). It can support cellular bioenergetics and prevent Alzheimer's disease by maintaining mitochondrial function in APP/PS1 mice and neurons (18). It can prevent 6-hydroxydopamine-induced mitochondrial dysfunction (39). In this study, both in vivo and in vitro experiments confirmed that exogenous mitochondrial targeting of AP39 ameliorates DOX-induced oxidative stress by decreasing cardiomyocyte ROS levels, elevating SOD and GSH-Px contents, and decreasing MDA and NADPH levels; it improved cardiomyocyte apoptosis by regulating the expression of apoptosis-related proteins, such as Bax, Bcl-2, and Cleaved Caspase-3/ Caspase-3, and improved DOX-induced

mitochondrial injury by elevating mitochondrial membrane potential and ATP levels, consistent with results of previous studies on the mechanisms underlying the myocardial protective effects of H_2S or AP39.

Cardiac tissues have high metabolic energy requirements, and growing evidence suggests that AMPK plays a key role as an energy sensor and a major regulator of metabolism in regulating cell survival in vivo and in vitro (40). In 2005, Tokarska-Schlattner et al. were the first to demonstrate that AMPK inactivation plays an important role in DOX cardiotoxicity (41). Since then, additional studies have shown that AMPK is closely related to multiple molecular mechanisms underlying DOX-induced cardiomyocyte injury. DOX is able to inhibit the expression and phosphorylation of AMPK proteins in the rat heart via DNA damage-induced Akt signaling, which activates a negative feedback loop of mTOR signaling and leads to cardiac remodeling (42). DOX can lead to myocardial fibrosis and cardiomyocyte apoptosis in APN-SE mice by inhibiting AMPK expression (43). Some AMPK activators, such as metformin, statins, resveratrol, and thiazolidinediones, have the potential to prevent DOX cardiotoxicity (44). Located within the mitochondrial membrane, UCP2 acts as an anion carrier and regulates the transmembrane proton electrochemical gradient in many human tissues; it is involved in a number of processes, including mitochondrial membrane potential, ROS production within the mitochondrial membrane, and calcium homeostasis (45). UCP2 is involved in the reduction of ROS production and mitochondrial ROS

scavenging (46) and can protect cardiomyocytes from oxidative stress by inhibiting ROS production (47). UCP2 prevents neuronal apoptosis and attenuates brain dysfunction after stroke and traumatic brain injury (48). UCP2 protects the heart from I/R injury by inducing mitochondrial autophagy (49). Studies on the interaction between AMPK and UCP2 have yielded conflicting results. It has been suggested that UCP2 affects the autophagic process in septic cardiomyopathy via AMPK signaling (50) an regulates cholangiocarcinoma cell plasticity via mitochondrial-AMPK signaling (51). However, there is substantial evidence that AMPK functions upstream of UCP2. For example, in a model of nonalcoholic fatty liver disease, LB100 regulated UCP2 expression by inhibiting AMPK (21). Malvidin alleviates mitochondrial dysfunction and ROS accumulation by activating the AMPK-α/UCP2 axis, thereby preventing inflammation and apoptosis in SAE mice (10). Indole sulfate induces oxidative stress and hypertrophy in cardiomyocytes by inhibiting the AMPK/UCP2 signaling pathway (52). In our experiments, we found that the protective effect of AP39 against DOX cardiotoxicity was mediated by AMPK/UCP2, and the use of AMPK inhibitors affected the expression of UCP2, while the inhibition of UCP2 expression did not have a significant effect on the expression level of AMPK. These findings suggest that AMPK is an upstream signal of UCP2 and regulates the expression of UCP2. The differences in the regulatory relationship between AMPK and UCP2 among studies may be related to differences in disease models, stimuli, and

other factors. The present study clarifies the role of the AMPK regulation of UCP2 in the attenuation of DOX cardiotoxicity by AP39; however, the specific mechanism underlying these regulatory effects is not clear. A downstream pathway of AMPK is Sirt1/PGC-1α, and AMPK activates the NAD+-dependent type III deacetylase Sirt1 by increasing the intracellular NAD+/NADH ratio; Sirt1 activation leads to peroxisome proliferation-activated receptor-γ coactivator 1α (PGC-1α) deacetylation and activity regulation, and Sirt1/PGC-1α may be involved in the regulation of UCP2 (8). Accordingly, the roles of Sirt1/PGC-1α need to be studied further. Despite these limitations, our experimental results provide possible therapeutic strategies for DOX cardiotoxicity and support the beneficial effects of AP39.

5 Conclusions

Taken together, our findings suggest that AP39 ameliorates DOX cardiotoxicity by attenuating oxidative stress, apoptosis, and mitochondrial damage via the modulation of AMPK/UCP2 expression. These findings indicate that AP39 is a promising new therapeutic agent for preventing DOX-induced cardiotoxicity.

CRediT authorship contribution statement

Bin Zhang: Conceptualization,Methodology,Validation,Formal analysis,Writing - Original Draft;Yangxue Li:Formal analysis,Writing - Review & Editing;Ning Liu: Resources,Writing - Review & Editing,Project administration;Bin Liu*:

Resources,Supervision,Project administration,Funding acquisition.

Declaration of Competing Interest

The authors declare no competing interests.

Funding sources

This work was supported by Jilin Province Science and Technology Department (20220303002SF), Jilin Provincial Development and Reform Commission (2022C003), Jilin Province Science and Technology Department (20190905002SF).

【References】

1、Sun J, Wei Q, Zhou Y, Wang JQ, Liu Q, Xu H. A systematic analysis of FDA-approved anticancer drugs. BMC Syst Biol 2017; 11: 87. doi:10.1186/ s12918 -017-0464-7.

2、Omland T, Heck SL, Gulati G. The role of cardioprotection in cancer therapy cardiotoxicity: JACC: cardiooncology state-of-the-art review. JACC CardioOncol 2022;4:19–37. doi:10.1016/j.jaccao. 2022.01.101.

3、Rawat PS, Jaiswal A,Khurana A.Doxorubicin-induced cardiotoxicity: An update on the molecular mechanism and novel therapeutic strategies for effective management. Biomedicine & Pharmacotherapy 2021 Jul;139:111708. doi:10.1016/j.biopha.2021.111708.

4、Jones IC, Dass CR.Doxorubicin-induced cardiotoxicity: causative factors and possible interventions.Journal of Pharmacy and Pharmacology, 2022, 74, 1677–1688.doi:10.1093/jpp/rgac063.

5、Ceruso MS, Napolitano A, Silletta M,Mazzocca A, Valeri S, Improta L, Santini D, Tonini G, Badalamenti G, Vincenzi B. Use of Cardioprotective Dexrazoxane Is Associated with Increased Myelotoxicity in Anthracycline- Treated Soft-Tissue Sarcoma Patients. Chemotherapy 2019; 64: 105–9. doi:10.1159/000501195.

6、Timm KN, Tyler DJ,The Role of AMPK Activation for Cardioprotection in Doxorubicin-Induced Cardiotoxicity.Cardiovascular Drugs and Therapy (2020) 34:255–269.do:10.1007/s10557-020-06941-x.

7、Kim J, Kundu M,Viollet B ,Guan KL,AMPK and mTOR regulate autophagy through direct phosphorylation of Ulk1.Nat Cell Biol. 2011 Feb; 13(2): 132－141. doi: 10.1038/ncb2152.

8、Huang J, Liu W, Doycheva DM,Gamdzyk M, Lu WT, Tang JP, Zhang JH.Ghrelin attenuates oxidative stress and neuronal apoptosis via GHSR-1α/AMPK/Sirt1/PGC-1α/UCP2 pathway in a rat model of neonatal HIE.Free Radic Biol Med. 2019 Sep;141:322-337. doi: 10.1016/j.freeradbiomed.2019.07.001.

9、Liu D, Ma Z, Di SY, Yang Y, Yang JG, Xu LQ, Reiter RJ, Qiao SB, Yuan JS.AMPK/PGC1α activation by melatonin attenuates acute doxorubicin cardiotoxicity via alleviating mitochondrial oxidative damage and apoptosis.Free Radic Biol Med. 2018 Dec;129:59-72. doi:10.1016/ j.freeradbiomed. 2018.08.032.

10、Zhao PP, Li XM, Yang QK, Lu YZ, Wang GL, Yang HT, Dong JQ, Zhang HG.Malvidin alleviates mitochondrial dysfunction and ROS accumulation through activating AMPK-α/UCP2 axis, thereby resisting inflammation and apoptosis in SAE mice.Front Pharmacol 2023 Jan 9;13:1038802. doi: 10.3389/fphar.2022.1038802. eCollection 2022.

11、S. R. Lee, B. Nilius, and J. Han, “Gaseous signaling molecules in cardiovascular function: from mechanisms to clinical translation,” Reviews of Physiology, Biochemistry and Pharmacology,2018;174:81-156. doi:10.1007/112_2017_7.

12、Wang YZ,Ngowi EE,Wang D,Qi HW,Jing MR, Zhang YX, Cai CB, He QL, Khattak S, Khan NH, Jiang QY, Ji X Y, Wu DD . The Potential of Hydrogen Sulfide Donors in Treating Cardiovascular Diseases.Int. J. Mol. Sci. 2021, 22, 2194.doi:10.3390/ijms22042194.

13、Karwi QG,Bornbaum J,Boengler K, Torregrossa R, Whiteman M, Wood ME, Schulz R, Baxter GF .AP39, a mitochondria-targeting hydrogen sulfide (H(2) S) donor, protects against myocardial reperfusion injury independently of salvage kinase signalling[J].Br J Pharmacol,2017, 174(4):287-301.doi:10.1111/bph.13688. Epub 2017 Jan 24.

14、Szczesny B, Módis1K, Yanagi K, Coletta C,Trionnaire SL, Wood ME, Whiteman M, Szabo C. AP39[10-oxo-10-(4-(3-thioxo-3H-1,2-dithiol-5yl) phenoxy)decyl) triphenylphosphonium bromide], a mitochondrially targeted hydrogen sulfide donor, stimulates cellular bioenergetics, exerts cytoprotective effects and protects against the loss of mitochondrial DNA integrity in oxidatively stressed endothelial cells in vitro.Nitric Oxide. 2014 September 15; 41: 120–130. doi:10.1016/j.niox.2014.04.008.

15、Karwi QG, Bornbaum J, Boengler K, Torregrossa R, Whiteman M, Wood M E, Schulz R, Baxter GF.AP39, a mitochondria-targeting hydrogen sulfide (H2 S) donor, protects against myocardial reperfusion injury independently of salvage kinase signalling.Br J Pharmacol . 2017 Feb;174(4):287-301. doi: 10.1111/bph.

13688. Epub 2017 Jan 24.

16、Yang T, Yang Q, Lai Q, Zhao JX, Nie LG, Liu SQ,Yang J, Chu C.AP39 inhibits ferroptosis by inhibiting mitochondrial autophagy through the PINK1/parkin pathway to improve myocardial fibrosis with myocardial infarction.Biomed Pharmacother. 2023 Sep;165:115195. doi: 10.1016/j.biopha.2023. 115195. Epub 2023 Jul 27.

17、Ding M, Shi R, Fu F , Li M, De D, Du YY, Li ZF.Paeonol protects against doxorubicin-induced cardiotoxicity by promoting Mfn2-mediated mitochondrial fusion through activating the PKCε-Stat3 pathway.J Adv Res. 2023 May; 47: 151 – 162. doi: 10.1016/j.jare.2022.07.002.

18、Zhao FL, Fang F, Qiao PF, Yan N, Gao D, Yan Y.AP39, a Mitochondria-Targeted Hydrogen Sulfide Donor, Supports Cellular Bioenergetics and Protects against Alzheimer's Disease by Preserving Mitochondrial Function in APP/PS1 Mice and Neurons.Oxid Med Cell Longev. 2016; 2016: 8360738. doi: 10.1155 /2016/8360738.

19、Yan WJ, Zhang HF, Liu PL, Wang H, Liu JY, Gao C, Liu Y, Lian K, Yang L, Sun L, et al.Impaired mitochondrial biogenesis due to dysfunctional adiponectin-AMPK-PGC-1alpha signaling contributing to increased vulnerability in diabetic heart.Basic Res. Cardiol. 108 (3) (2013) 329.doi：10.1007/s00395-013-0329-1.

20、Nam KN, Choi YS, Jung HJ, Park GH, Park JM, Moon SK , Cho KH, Kang C, Kang I, Oh MS, Lee EH.Genipin inhibits the inflammatory

response of rat brain microglial cells.Int Immunopharmacol. 2010 Apr;10(4):493-9. doi: 0.1016 /j.intimp .2010.01.011. Epub 2010 Feb 1.

21、Chen XY, Cai CZ, Yu ML, Feng ZM, Zhang YW, Liu PH, Zeng H, Yu CH.LB100 ameliorates nonalcoholic fatty liver disease via the AMPK/ Sirt1 pathway. World J. Gastroenterol. 25 (45), 6607–6618. doi:10.3748/wjg.v25. i45.6607.

22、M.S. Horenstein, R.S. Vander Heide, T.J. L’Ecuyer, Molecular basis of anthracycline-induced cardiotoxicity and its prevention, Mol. Genet. Metab. 71 (1–2) (2000) 436–444. doi：10.1006/mgme.2000.3043.

23、Ghigo A, Li N, Hirsch E, New signal transduction paradigms in anthracycline- induced cardiotoxicity, Biochim. Biophys. Acta 1863 (7 Pt B) (2016) 1916–1925. doi:10.1016/j.bbamcr.2016.01.021.

24、Fadillioglu, E, Oztas, E, Erdogan, H, Yagmurca M, Sogut S, Ucar M, Irmak MK. Protective effects of caffeic acid phenethyl ester on doxorubicin-induced cardiotoxicity in rats. Journal of Applied Toxicology: An International Journal, 24, 47–52.doi：10.1002/jat.945.

25、Alkreathy H, Damanhouri Z A, Ahme N, Slevin M, Ali SS, Osman AM. Aged garlic extract protects against doxorubicin-induced cardiotoxicity in rats. Food and Chemical Toxicology, 48, 951–956.doi：10.1016/j.fct.2010.01.005Get rights and content.

26、Yu W, Qin X, Zhang Y, Qiu P, Wang L, Zha WL, Ren J. Curcumin suppresses doxorubicin-induced cardiomyocyte pyroptosis via a

PI3K/Akt/mTOR- dependent manner. Cardiovascular Diagnosis and Therapy, 10, 752–769. doi：10. 21037/ cdt- 19- 707.

27、Shan YX, Liu TJ, Su HF, Samsamshariat A, Mestril R,Wang PH. Hsp10 and Hsp60 modulate Bcl-2 family and mitochondria apoptosis signaling induced by doxorubicin in cardiac muscle cells. Journal of Molecular and Cellular Cardiology, 35, 1135–1143. doi：10. 1016/ s0022- 2828(03) 00229-3.

28、Zhao D, Xue C, Li J, Feng K, Zeng P, Chen YL, Duan YJ, Zhang S, Li XJ, Han JH, Yang XX. Adiponectin agonist ADP355 ameliorates doxorubicin-induced cardiotoxicity by decreasing cardiomyocyte apoptosis and oxidative stress. Biochemical and Biophysical Research Communications, 533, 304–312.doi：10.1016/j.bbrc.2020.09.035.

29、M. Schlame, D. Rua, M.L. Greenberg, The biosynthesis and functional role of cardiolipin, Prog. Lipid Res. 39 (3) (2000) 257–288. do:10.1016/S0163-7827（00）00005-9.

30、Lebrecht D, Setzer B, Ketelsen UP, Haberstroh JR,Walker UA. Time-dependent and tissue-specific accumulation of mtDNA and respiratory chain defects in chronic doxorubicin cardiomyopathy. Circulation, 108, 2423–2429. doi:10.1161/01.CIR.0000093196.59829.DF.

31、Montaigne D, Marechal X, Baccouch R, Modine T, Preau S, Zannis K, Marchetti P, Lancel S, Neviere R. Stabilization of mitochondrial membrane potential prevents doxorubicin -induced cardiotoxicity in isolated rat heart. Toxicology and Applied Pharmacology, 244, 300–307. doi:10.1016

/j.taap.2010.01.006.
32、Beak J, Huang W, Parker JS, Hicks ST, Patterson C, Simpson PC, Ma AQ, Jin J, Jensen BC. An oral selective alpha-1A adrenergic receptor agonist prevents doxorubicin cardiotoxicity. JACC. Basic to translational science, 2, 39–53. doi：10. 1016/j. jacbts. 2016. 10. 006.
33、Wang R.Physiological implications of hydrogen sulfide: a whiff exploration that blossomed.Physiol Rev. 2012 Apr;92(2):791-896. doi: 10.1152/physrev. 00017. 2011.
34、Szabõ C. Hydrogen sulphide and its therapeutic potential. Nature Reviews Drug Discovery. 2007;6(11):917–935. doi: 10.1038/nrd2425.
35、Calvert JW, Coetzee WA, Lefer DJ. Novel insights into hydrogen sulfide-mediated cytoprotection. Antioxidants & Redox Signaling.2010;12(10) : 1203–1217. doi: 10.1089/ars.2009.2882.
36、Elrod JW, Calvert JW, Morrison J, Doeller JE, Kraus DW, Tao L, Jiao XY, Scalia R, Kiss L, Szabo C,et al. Hydrogen sulfide attenuates myocardial ischemia-reperfusion injury by preservation of mitochondrial function. Proceedings of the National Academy of Sciences of the United States of America. 2007;104(39):15560–15565.doi: 10.1073/pnas. 0705891104.
37、Zhou X, Lu X. Hydrogen sulfide inhibits high-glucose-induced apoptosis in neonatal rat cardiomyocytes. Experimental Biology and Medicine. 2013;238(4): 370–374. doi: 10.1177/1535370213477989.

38、Yu Y, Ye SM, Liu DY and Yang LQ.AP39 ameliorates high fat diet-induced liver injury in young rats via alleviation of oxidative stress and mitochondrial impairment.Exp Anim. 2021; 70(4): 553 – 562.doi: 10.1538/expanim.21-0056.

39、Marwah MK, Manhoosh B, Shokr H, Tahan MAA, Stewart R, Iqbal M, Sanchez LD, Abdullah S, Ahmad S, Wang K Q, Rana KS, Sanchez-Aranguren L.Transdermal delivery of mitochondrial-targeted hydrogen sulphide donor, AP39 protects against 6-hydroxydopamine-induced mitochondrial dysfunction.Eur J Pharm Biopharm. 2023 Sep 4;S0939-6411(23)00237-0. doi: 10.1016/j.ejpb. 2023.09.004.

40、Ramamurthy S, Ronnett GV.Developing a head for energy sensing: AMP-activated protein kinase as a multifunctional metabolic sensor in the brain.J Physiol. 2006 Jul 1;574(Pt 1):85-93. doi: 10.1113/jphysiol.2006.110122.

41、Tokarska-Schlattner M, Zaugg M, Da Silva R, Lucchinetti E,Schaub MC, Wallimann T, Schlattner U. Acute toxicity of doxorubicin on isolated perfused heart: Response of kinases regulating energy supply. Am J Physiol - Hear Circ Physiol. 2005;289.doi:10.1152/ajpheart.01057.2004.

42、Gratia S, Kay L, Potenza L, Seffouh A, Novel-Chate V ,Schnebelen C, Sestili P, Schlattner U, Tokarska-Schlattner M. Inhibition of AMPK signalling by doxorubicin: At the crossroads of the cardiac responses to energetic, oxidative, and genotoxic stress. Cardiovasc Res. 2012;95:290 – 9.doi：10.1093/cvr/cvs134.

43、Konishi M, Haraguchi G, Ohigashi H, Ishihara T, Saito K, Nakano Y, Isobe M.Adiponectin protects against doxorubicin-induced cardiomyopathy by anti-apoptotic effects through AMPK up-regulation.Cardiovasc Res. 2011 Feb 1;89(2):309-19. doi: 10.1093/cvr/cvq335. Epub 2010 Oct 25.

44、Timm KN, Tyler DJ.The Role of AMPK Activation for Cardioprotection in Doxorubicin-Induced Cardiotoxicity.Cardiovascular Drugs and Therapy (2020) 34:255 – 269.doi:10.1007/s10557-020-06941-x.

45、Stanzione R,Forte M, Cotugno M, Bianchi F, Marchitti S, Busceti CL, Fornai F, Rubattu S. Uncoupling Protein 2 as a Pathogenic Determinant and Therapeutic Target in Cardiovascular and Metabolic Diseases .Current Neuropharmacology, 2022, 20, 662-674.doi: 10.2174/1570159X19666210421094204.

46、Rubattu S, Stanzione R, V olpe M. Mitochondrial Dysfunction Contributes to Hypertensive Target Organ Damage: Lessons from an Animal Model of Human Disease. Oxidative medicine and cellular longevity 016;2016:1067801. doi: 10.1155/2016/1067801.

47、Teshima Y, Akao M, Jones SP, Marbán E.Uncoupling protein-2 overexpression inhibits mitochondrial death pathway in cardiomyocytes.Circ Res. 2003 Aug 8;93(3):192-200. doi: 10.1161/01.RES.0000085581.60197.4D.

48、Mehta SL, Li PA. Neuroprotective role of mitochondrial uncoupling protein 2 in cerebral stroke. J Cereb Blood Flow Metab. 2009 Jun;29(6):1069-78. doi: 10.1038/jcbfm.2009.4.

49、Wu H,Ye M,Liu D,Yang J, Ding JW, Zhang J, Wang XA, Dong WS, Fan ZX, Yang J.UCP2 protect the heart from myocardial ischemia/reperfusion injury via induction of mitochondrial autophagy. J. Cell. Biochem., 2019, 120(9), 15455-15466. doi：10.1002/jcb.28812.

50、Mao JY, Su LX, Li DK, Zhang HM, Wang XT, Liu DW.The effects of UCP2 on autophagy through the AMPK signaling pathway in septic cardiomyopathy and the underlying mechanism.Ann Transl Med 2021;9(3):259.doi：10.21037/atm-20-4819.

51、Yu JH,Shi L, Shen XG,Zhao YF.UCP2 regulates cholangiocarcinoma cell plasticity via mitochondria-to-AMPK signals.Biochem Pharmacol. 2019 Aug; 166: 174－184.doi: 10.1016/j.bcp.2019.05.017.

52、Yang K, Xu XL, Nie L, Xiao TL, Guan X, He T, Yu YL, Liu L, Huang YJ, Zhang JB, Zhao JH.Indoxyl sulfate induces oxidative stress and hypertrophy in cardiomyocytes by inhibiting the AMPK/UCP2 signaling pathway.Toxicol Lett. 2015 Apr 16;234(2):110-9. doi: 10.1016/j.toxlet. 2015.01.021. Epub 2015 Feb 19.

Figure1 Click here to access/download;Figure;FIG1.TIF.tif

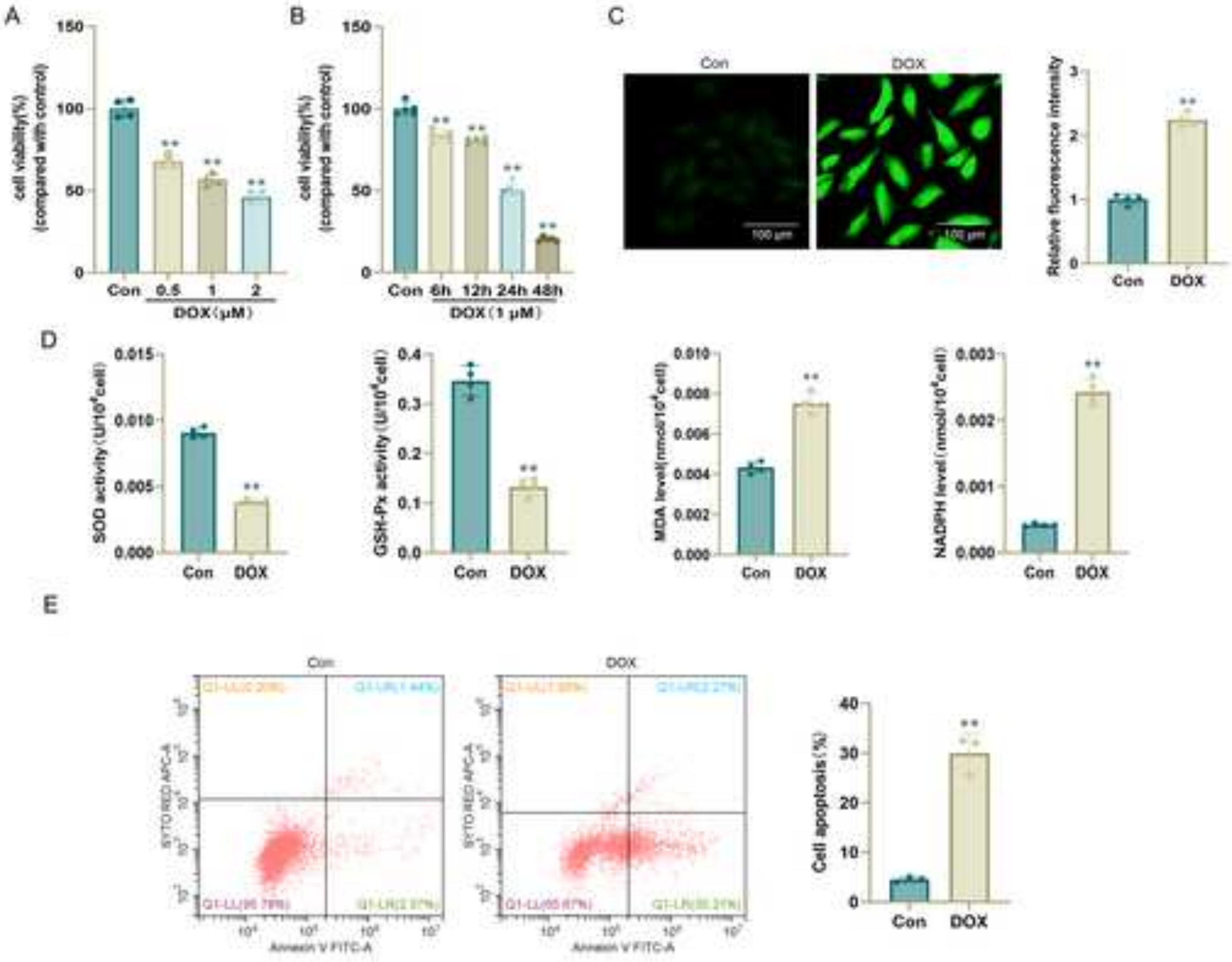

Figure 1 DOX induces H9c2 cell damage. (A) and (B) Cell viability determined by CCK-8 assays after treatment with DOX at different concentrations for 24 h and treatment with 1 μmol/L DOX for different times (n = 4 or 5); (C) Representative DCFH-DA images and statistical results (n = 4); (D) SOD, GSH-Px, MDA, and NADPH levels in H9c2 cells (n = 4); (E) Apoptosis rate measured by flow cytometry (n = 3).Values represent the mean ± SD. *p < 0.05 vs. Con group, **p < 0.01 vs. Con group.

Figure2 Click here to access/download;Figure;FIG2.TIF.tif

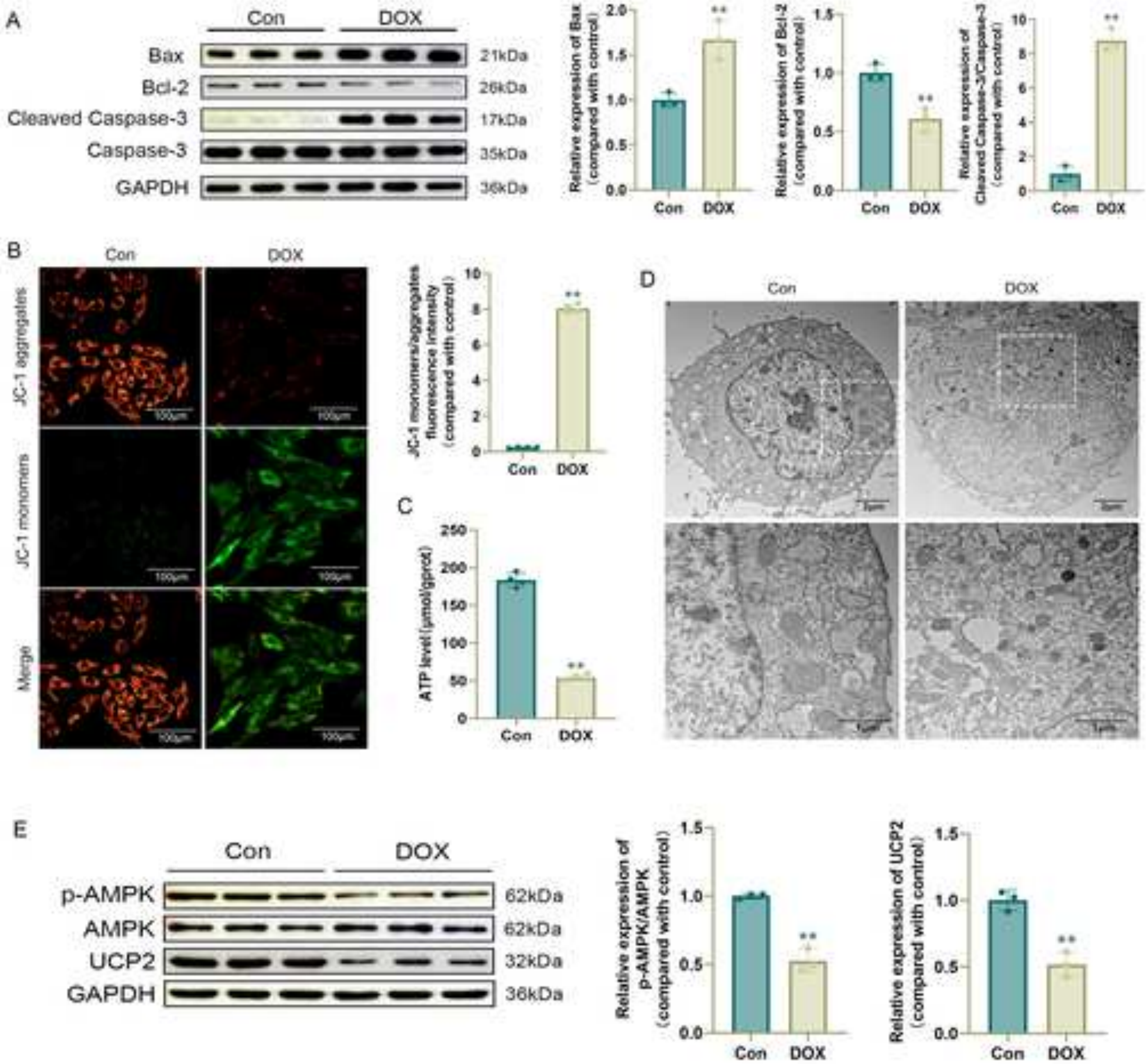

Figure 2 DOX induces mitochondrial damage in H9c2 cells. (A) Western blot detection of apoptosis-related protein levels and statistical results (n = 3); (B) Representative JC-1 images and quantification of fluorescence intensity for JC-1 monomers/aggregates (n = 4); (C) ATP level (n = 4); (D) Representative images of mitochondria in H9c2 cells observed by transmission electron microscopy; (E) Western blot detection of p-AMPK, AMPK, and UCP2 levels and statistical results (n = 3). Values are presented as the mean ± SD. *p < 0.05 vs. Con group, **p < 0.01 vs. Con group.

Figure3 Click here to access/download;Figure;FIG3.TIF.tif

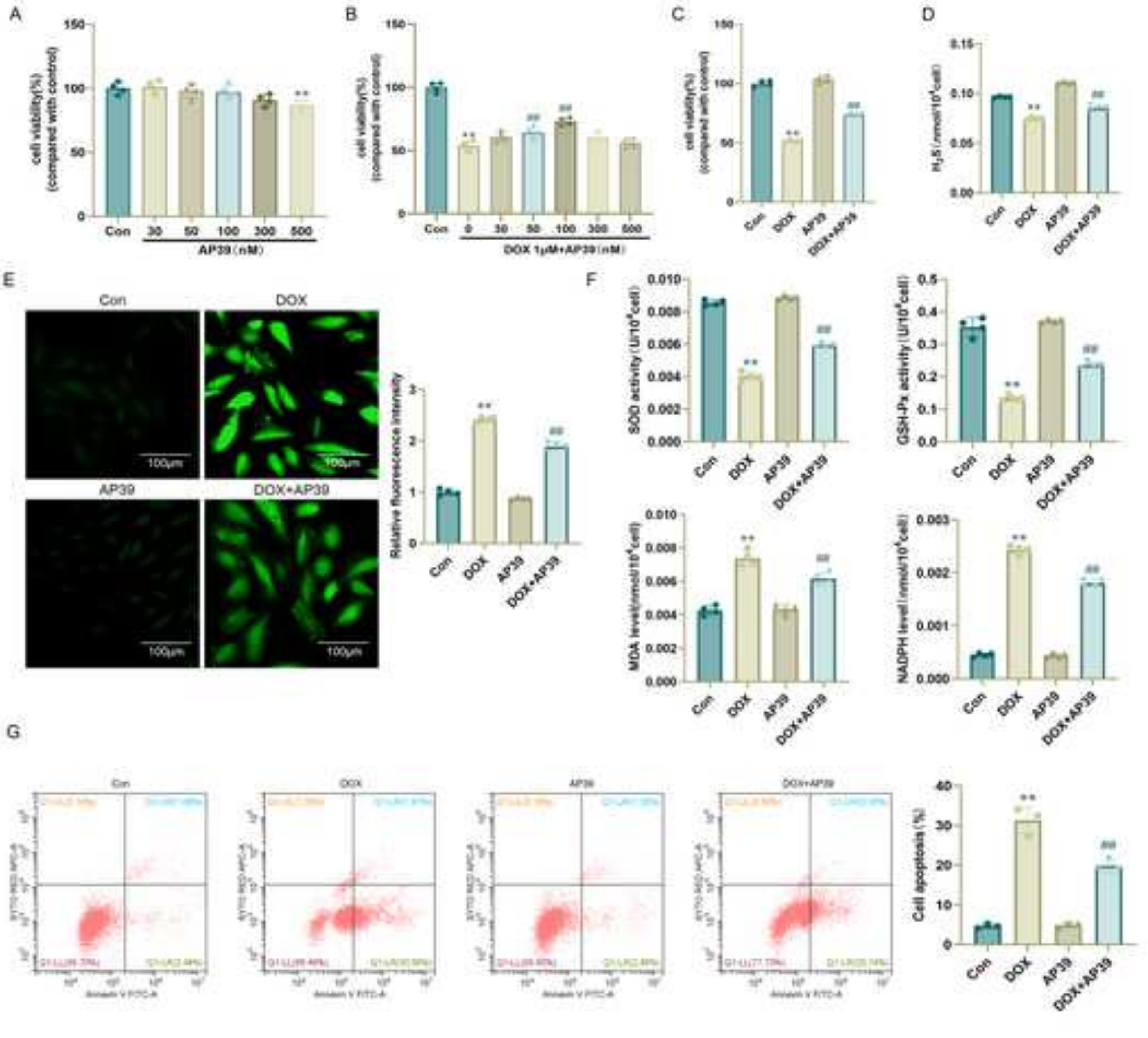

Figure 3 AP39 ameliorates DOX-induced myocardial injury. (A)-(C) Cell viability determined by CCK-8 assays after H9c2 cells were treated with different concentrations of AP39 for 24 h, 1 μmol/L DOX, and different concentrations of AP39 for 24 h (n = 4); (D) H2S contents (n = 4); (E) Representative DCFH-DA images and statistical results (n = 4); (F) SOD, GSH-Px, MDA, and NADPH levels in H9c2 cells (n = 4); (G) Apoptosis rate measured by flow cytometry (n = 3). Values are presented as the mean ± SD. *p < 0.05 vs. Con group, **p < 0.01 vs. Con group. #p < 0.05 vs. DOX group, ##p < 0.01 vs. DOX group.

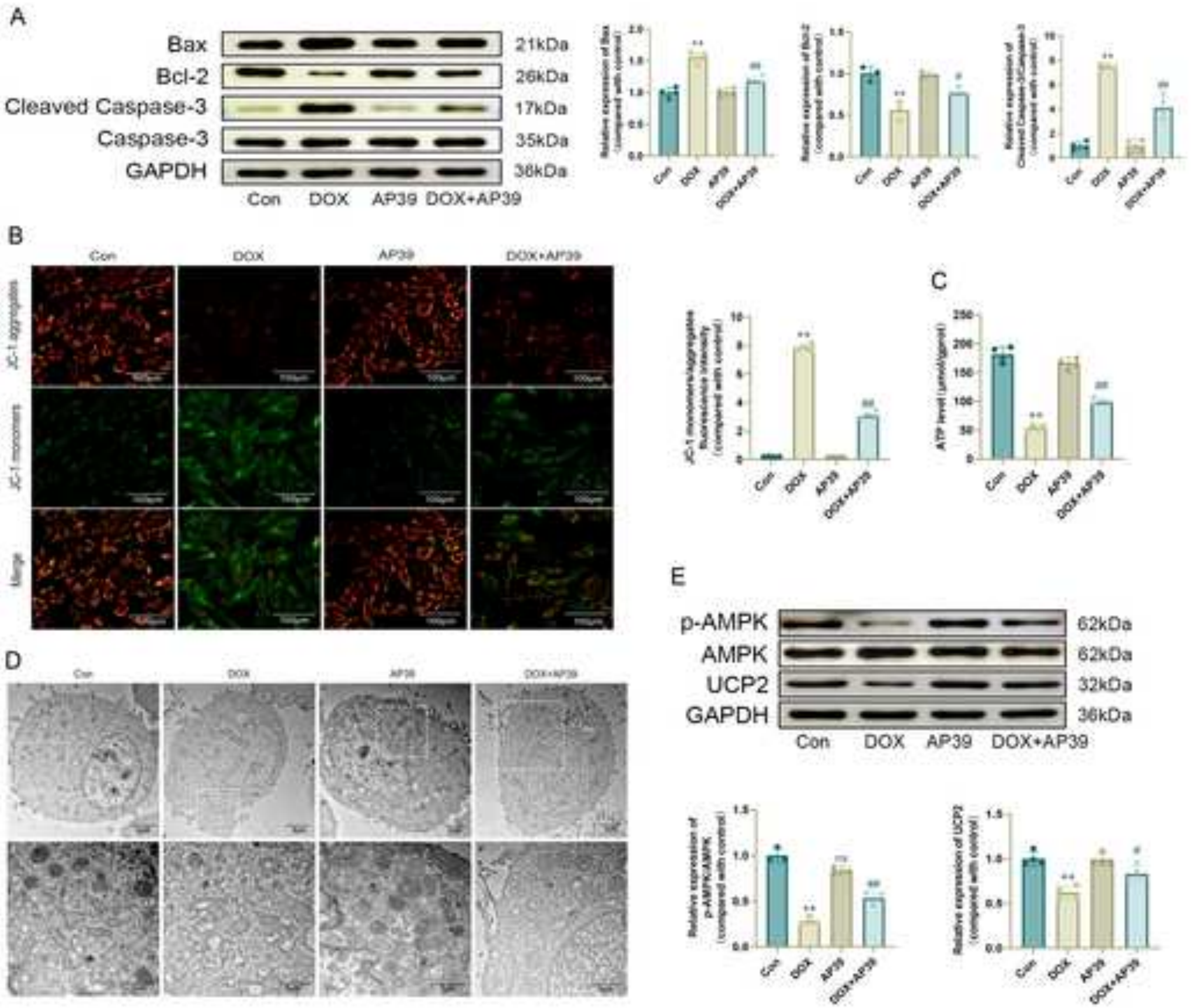

Figure 4 AP39 ameliorates DOX-induced mitochondrial damage. (A) Western blot detection of apoptosis-related protein levels and statistical results (n = 3 or 4); (B) Representative JC-1 images and quantification of fluorescence intensity for JC-1 monomers/aggregates (n = 4); (C) ATP level (n = 4); (D) Representative images of mitochondria in H9c2 cells observed by transmission electron microscopy; (E) Western blot detection of p-AMPK, AMPK, and UCP2 levels and statistical results (n = 3 or 4). Values are presented as the mean ± SD. *p < 0.05 vs. Con group, **p < 0.01 vs. Con group. #p < 0.05 vs. DOX group, ##p < 0.01 vs. DOX group. NS indicates no significant difference vs. Con group.

Figure5 Click here to access/download;Figure;FIG5.TIF.tif

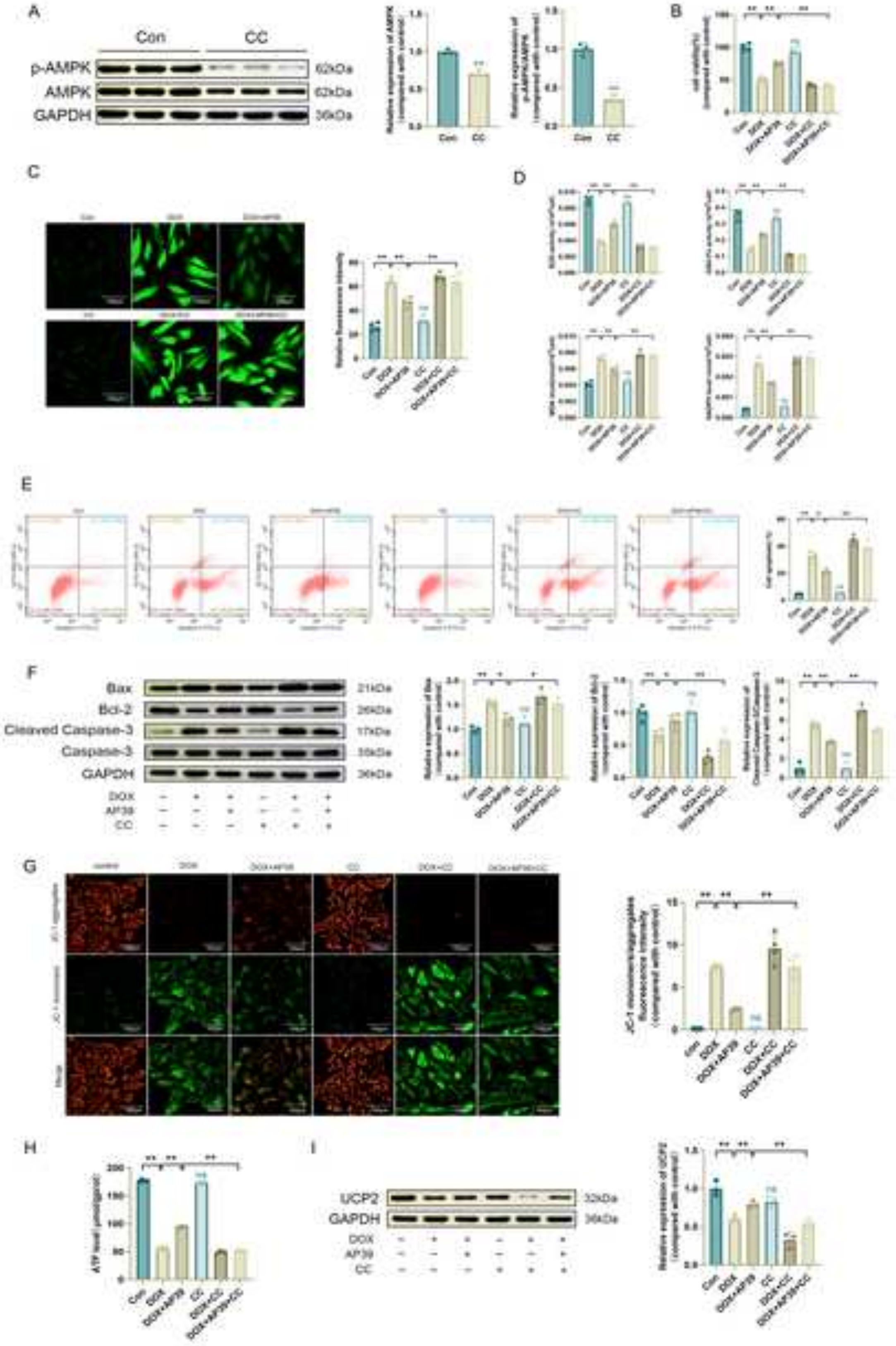

Figure 5 Inhibition of AMPK expression limits the beneficial effect of AP39 on DOX cardiotoxicity. (A) Western blot detection of p-AMPK and AMPK levels and statistical results (n = 3); (B) CCK-8 assay of cell viability (n = 4); (C) Representative DCFH-DA images and statistical results (n = 4); (D) SOD, GSH-Px, MDA, and NADPH levels in H9c2 cells (n = 4); (E) Apoptosis rate measured by flow cytometry (n = 3); (F) Western blot detection of apoptosis-related protein levels and statistical results (n = 3 or 4); (G) Representative JC-1 images and quantification of fluorescence intensity for JC-1 monomers/aggregates (n = 4); (H) ATP level (n = 4); (I) Western blot detection of UCP2 levels and statistical results (n = 3). Values are presented as the mean ± SD. *p < 0.05, **p < 0.01. NS indicates no significant difference vs. Con group.

Figure6

Click here to access/download;Figure;FIG6.TIF.tif

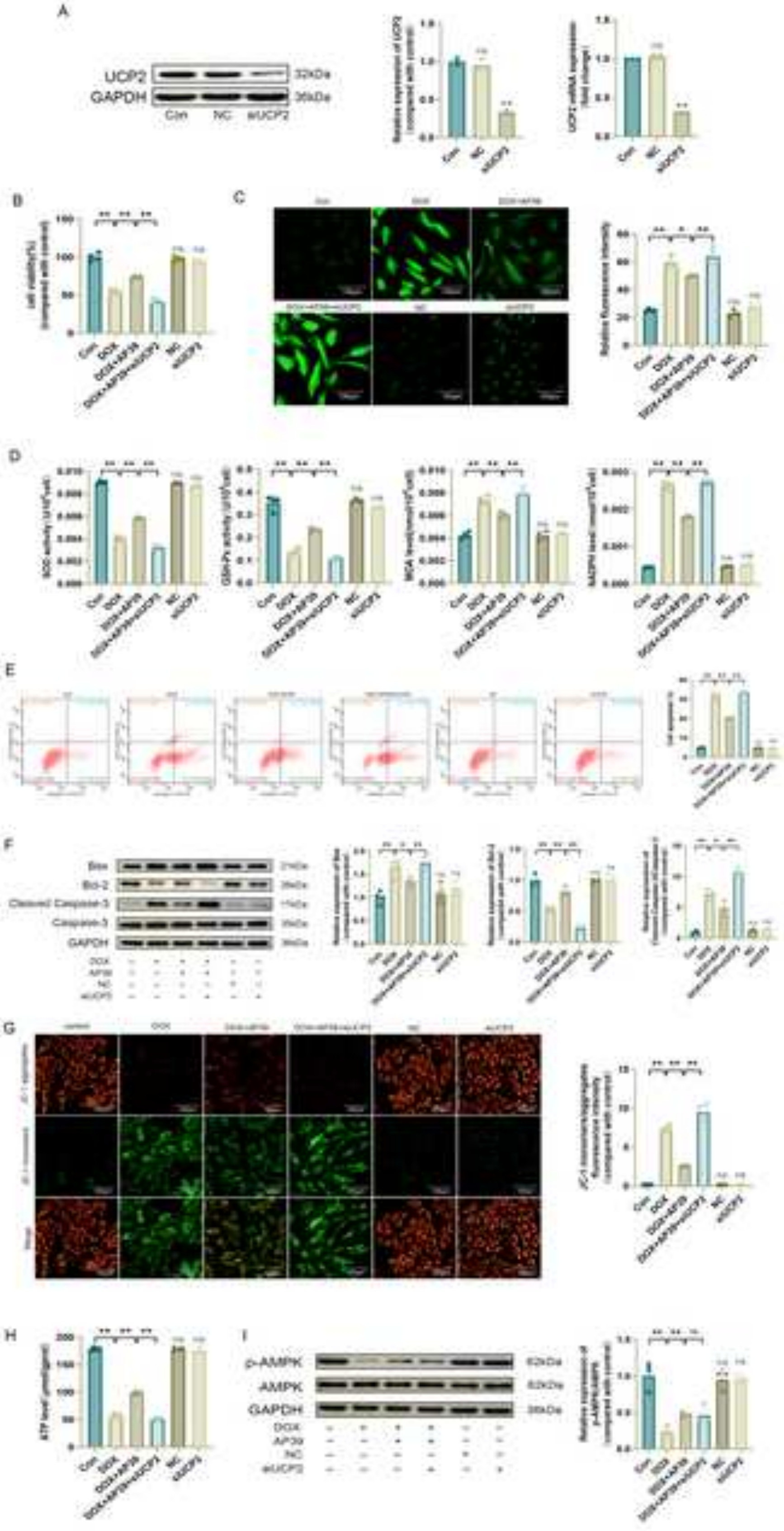

Figure 6 AP39 improves DOX-induced cardiotoxicity by preventing UCP2 down-regulation. (A) Western blot analysis of UCP2 (n = 4) and PCR for UCP2 mRNA levels (n = 3); (B) CCK-8 assay of cell viability (n = 4); (C) Representative DCFH-DA images and statistical results (n = 4); (D) SOD, GSH-Px, MDA, and NADPH levels in H9c2 cells (n = 4); (E) Apoptosis rate measured by flow cytometry (n = 3); (F) Western blot detection of apoptosis-related protein levels and statistical results (n = 3 or 4); (G) Representative JC-1 images and quantification of fluorescence intensity for JC-1 monomers/aggregates (n = 4); (H) ATP levels (n = 4); (I) Western blot detection of p-AMPK and AMPK levels and statistical results (n = 3). Values are presented as the mean ± SD. *p < 0.05, **p < 0.01. NS indicates no significant difference vs. Con group.

Figure7 Click here to access/download;Figure;FIG7.TIF..tif

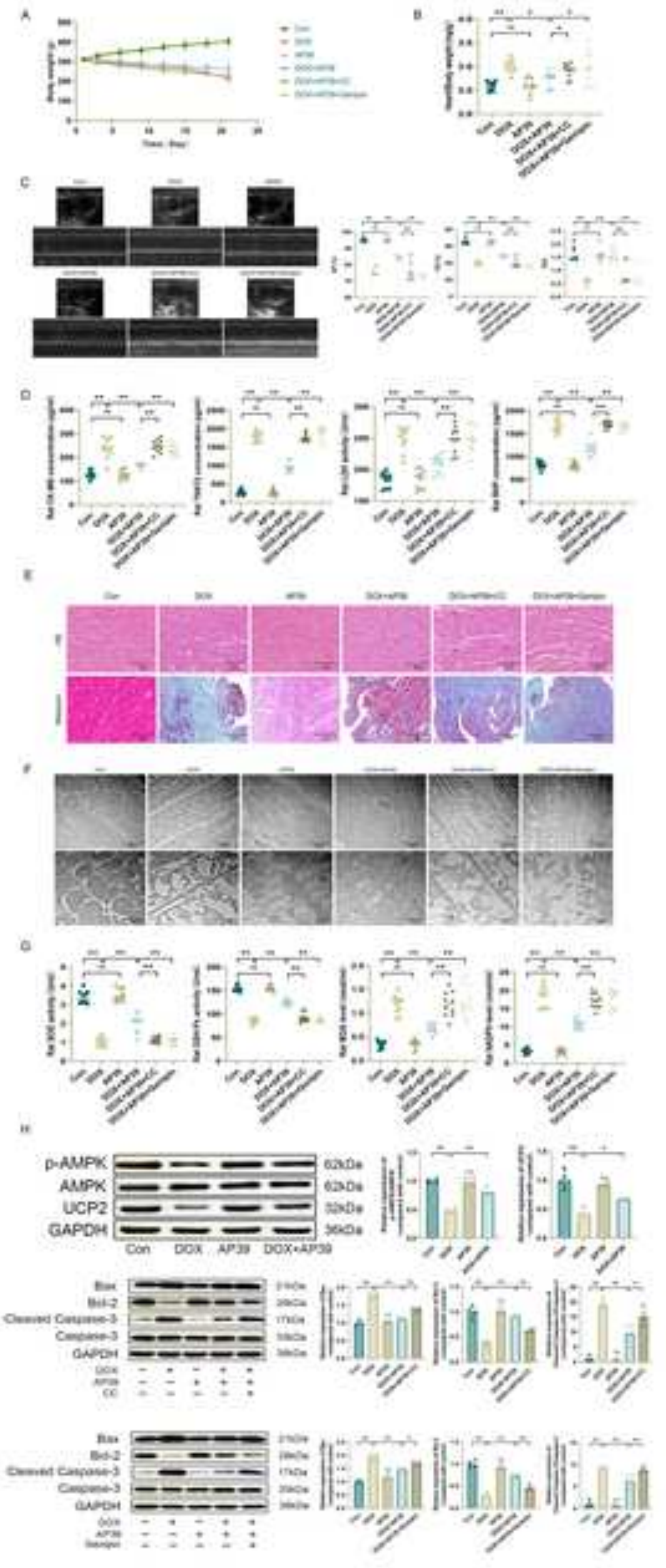

Figure 7 AP39 ameliorates DOX-induced cardiotoxicity in rats by regulating the AMPK/UCP2 pathway. (A) Body weight in different groups of rats (n = 10); (B) Heart/body weight ratio in different groups of rats (n = 10); (C) Representative echocardiographic images and quantitative analysis of EF%, FS%, and E/A (n = 7); (D) Serum TNNT2, CK-MB, LDH, and BNP levels in rats (n = 10); (E) Representative HE and Masson staining images of the rat myocardium; (F) Representative images of mitochondria in rat cardiomyocytes observed by transmission electron microscopy; (G) SOD, GSH-Px, MDA, and NADPH levels in rat serum (n = 10); (H) Western blot detection of apoptosis-related protein, p-AMPK, AMPK and UCP2 levels and statistical results (n = 3 or 4). Values are presented as the mean ± SD; *p < 0.05, **p < 0.01. NS indicates no significant difference vs. Con group.
