# Supplementary material for: AP39, a novel mitochondria-targeted hydrogen sulfide donor ameliorates doxorubicin-induced cardiotoxicity by regulating the AMPK/UCP2 pathway
Source: PLoS One. 2024 Apr 3;19(4):e0300261. doi: 10.1371/journal.pone.0300261 (PMC10990198; doi:10.1371/journal.pone.0300261)
Supplement: Attachment — Submitted filename: comments.docx [file pone.0300261.s006.docx]

I suggest title modification to explain what is AP39?

The suggested title will be as follow:

AP39, a novel mitochondria-targeted hydrogen sulfide donor ameliorates doxorubicin-induced cardiotoxicity by regulating the AMPK/UCP2 pathway

Pathogenesis of DOX cardiotoxicity should be covered in depth in introduction and discussion sections with reference to the possible mechanisms of potential cardioprotective agents. The following references might be helpful

https://doi.org/10.1016/j.biopha.2017.04.033

https://doi.org/10.1007/s00280-017-3413-7

https://doi.org/10.3389/fphar.2019.00635
