# Supplementary material for: AP39, a novel mitochondria-targeted hydrogen sulfide donor ameliorates doxorubicin-induced cardiotoxicity by regulating the AMPK/UCP2 pathway
Source: PLoS One. 2024 Apr 3;19(4):e0300261. doi: 10.1371/journal.pone.0300261 (PMC10990198; doi:10.1371/journal.pone.0300261)
Supplement: Attachment — Submitted filename: Response to Reviewers.docx [file pone.0300261.s007.docx]

**Dear editor and dear reviewers:**

**Thank you for your letter and the reviewers’ comments regarding our manuscript titled “AP39 ameliorates doxorubicin-induced cardiotoxicity by regulating the AMPK/UCP2 pathway” (ID: PONE-D-23-34322R1). The comments were all valuable and very helpful in amending and improving our paper, as well as our research. We have addressed all the comments and have made corrections to the manuscript (highlighted in yellow).We hope that the revised manuscript now meets the requirements of *PLOS ONE*. Our point-by-point responses to all comments are presented below:**

**Reviewer #3:**

***1.I suggest title modification to explain what is AP39?***

**Response:**

Thank you very much for your comment.

We have changed the title to ”AP39, a novel mitochondria-targeted hydrogen sulfide donor ameliorates doxorubicin-induced cardiotoxicity by regulating the AMPK/UCP2 pathway”as you suggested.

***2. Pathogenesis of DOX cardiotoxicity should be covered in depth in introduction and discussion sections with reference to the possible mechanisms of potential cardioprotective agents. The following references might be helpful.
 https://doi.org/10.1016/j.biopha.2017.04.033
 https://doi.org/10.1007/s00280-017-3413-7
 https://doi.org/10.3389/fphar.2019.00635***

**Response:**

Thank you very much for your comment.

Based on your suggestions and regarding the literature you provided, we have further discussed and improved the information concerning the pathogenesis of DOX-induced cardiotoxicity in the Introduction and Discussion sections,referencing the possible mechanisms of potential cardioprotective agents. All specific changes have been highlighted.

**We appreciate your earnest work reviewing our manuscript, which we have revised point-by-point per your suggestions. Once again, thank you very much for your comments and suggestions.**

**Kind Regards,**

**Bin Zhang,Yangxue Li,Ning Liu and Bin Liu**
